# A Formylglycine‐Peptide for the Site‐Directed Identification of Phosphotyrosine‐Mimetic Fragments**

**DOI:** 10.1002/chem.202201282

**Published:** 2022-08-23

**Authors:** Markus Tiemann, Eric Nawrotzky, Peter Schmieder, Leon Wehrhan, Silke Bergemann, Vera Martos, Wei Song, Christoph Arkona, Bettina G. Keller, Jörg Rademann

**Affiliations:** ^1^ Department of Biology, Chemistry, Pharmacy Institute of Pharmacy Freie Universität Berlin Königin-Luise-Strasse 2+4 14195 Berlin Germany; ^2^ Leibniz Institute of Molecular Pharmacology (FMP) Robert-Rössle-Strasse 10 13125 Berlin Germany; ^3^ Department of Biology, Chemistry, Pharmacy Institute of Chemistry and Biochemistry Freie Universität Berlin Arnimallee 22 14195 Berlin Germany

**Keywords:** fragment-based drug discovery, fragment ligation, formylglycine, phosphatase inhibitors, site-directed screening

## Abstract

Discovery of protein‐binding fragments for precisely defined binding sites is an unmet challenge to date. Herein, formylglycine is investigated as a molecular probe for the sensitive detection of fragments binding to a spatially defined protein site . Formylglycine peptide **3** was derived from a phosphotyrosine‐containing peptide substrate of protein tyrosine phosphatase PTP1B by replacing the phosphorylated amino acid with the reactive electrophile. Fragment ligation with formylglycine occurred in situ in aqueous physiological buffer. Structures and kinetics were validated by NMR spectroscopy. Screening and hit validation revealed fluorinated and non‐fluorinated hit fragments being able to replace the native phosphotyrosine residue. The formylglycine probe identified low‐affinity fragments with high spatial resolution as substantiated by molecular modelling. The best fragment hit, 4‐amino‐phenyl‐acetic acid, was converted into a cellularly active, nanomolar inhibitor of the protein tyrosine phosphatase SHP2.

## Graphical abstract

**Site‐directed screening**? Formylglycine (red) is a reactive amino acid that can be utilized in fragment ligation assays. Incorporated into a site‐directing hexapeptide it can effectively probe the active site of PTP1B, yielding new phosphotyrosine mimetics derived from different nucleophiles, such as aryl amines (green).

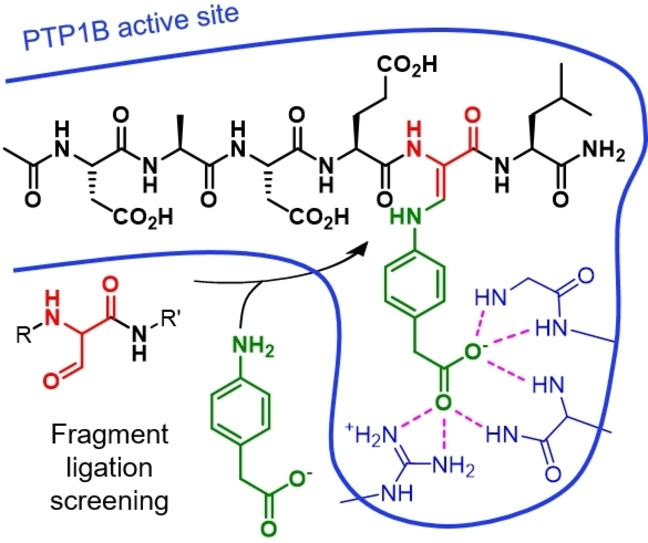

## Introduction

Site‐directed discovery of small protein‐binding fragments with M<250 Da is a challenge due to the low affinities of most fragments and due to the lack of analytical methods that enable the detection of binding fragments at precisely defined positions on the protein surface.^[1]^ NMR spectroscopy,^[2]^ surface plasmon resonance,^[3]^ and X‐ray crystallography,^[4]^ have often been used to identify binding of low‐affinity fragments, however, these methods are principally non‐selective for defined specific binding pockets and for defined orientations of fragments within these pockets that are required for fragment linking or fragment growing.^[5]^ In addition, these biophysical detections methods require very high fragments concentrations in the range or beyond *K_D_* values to enable detection.

Fragment ligation has been introduced as a method to enhance binding of a primary fragment by a covalent, typically protein‐templated reaction with a secondary ligand.^[6]^ The approach has been applied in dynamic ligation screening for the discovery of protein ligands useful as enzyme inhibitors,^[7]^ for substrate optimization^[8]^ and for inhibitors of protein‐protein interactions^[9]^ and a broad range of reversible and irreversible reactions have been employed. Peptides have been used as reversible and as irreversible[^6a^, ^10^] probes for fragment ligation. What is, however, missing, is a broader or even general method that enables to investigate ligands binding in precisely defined amino acid side chain pockets of peptide‐protein‐ and protein‐protein interaction sites.

**Figure 1:**
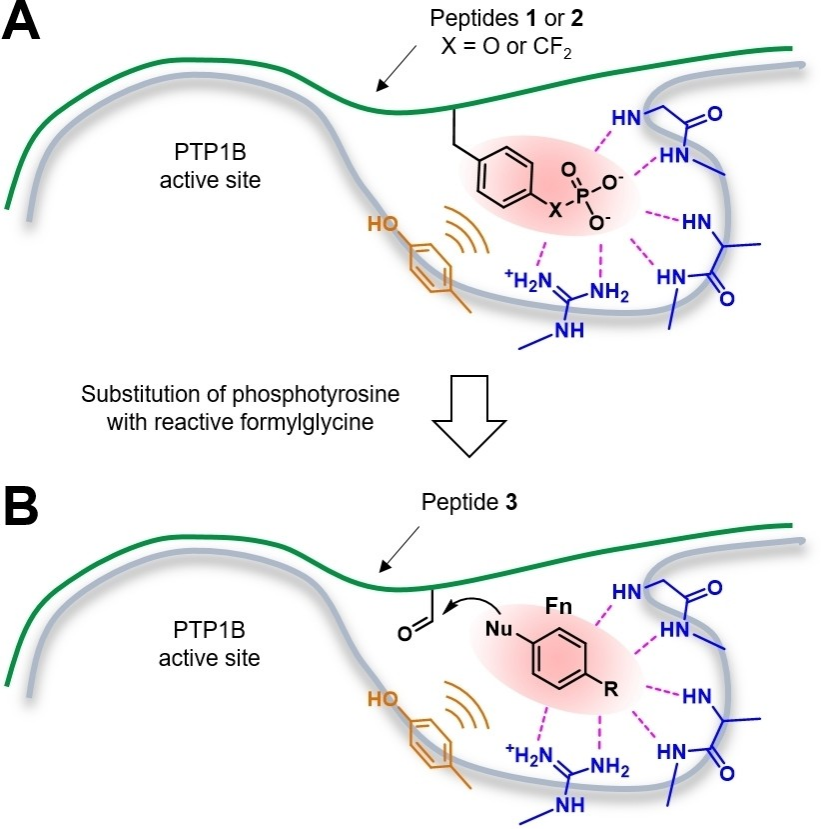
Site‐directed fragment screening using a formylglycine peptide. A) Native phosphotyrosine peptide **1** (X=O) or inhibitor **2** (X=CF_2_), with peptide backbone shown as a green line, bind to the active site of phosphotyrosine phosphatase PTP1B; B) Replacement of the phosphotyrosine by formylglycine furnishes formylglycine peptide **3** (green line) with residual affinity; Ligation with nucleophilic phosphotyrosine mimetic fragment **Fn** provide ligation products with increased affinity.

We reasoned that peptides and proteins carrying a reactive side chain electrophile should enable the site‐specific ligation of nucleophiles and thus might serve as tools for the site‐directed discovery of binding fragments for defined protein pockets (Figure 1). Formylglycine (fG) residues have been found as electrophilic post‐translational modifications in native peptides and proteins.^[11]^ They are generated from cysteine residues of protein sulfatases and are essential for the catalytic activity of these enzymes.^[12]^ The Cys‐containing recognition sequence has been incorporated into other proteins and converted using the formylglycine‐generating enzyme.^[13]^

These findings encouraged us to investigate formylglycine peptides for the site‐directed discovery of protein‐binding fragments. As a model system, we selected the active sites of protein tyrosine phosphatases (PTP) which recognize and hydrolyze phosphotyrosine residues (pTyr, Y*) as substrates.^[14]^ Mimetics of phosphotyrosine such as 4‐phosphono‐difluoromethyl‐phenylalanine (PDFM‐Phe), have been used as starting points for the development of chemical probes targeting both PTP and phosphotyrosine recognition domains such as the Src‐homology domain (SH2).^[15]^ Such probes have been proven successful for the validation of PTP as well as SH2 domains as potential pharmacological targets and may have the potential to be useful for the development of clinical candidates in the future.

To test the hypothesis, we took a natural substrate of the enzyme PTP1B, phosphotyrosine peptide **1** (X=O) or the potent inhibitor **2** derived from it (X=CF_2_) and replaced the phosphotyrosine residue by formylglycine (Figure 1). The obtained formylglycine peptide **3** was then used to identify phosphotyrosine mimetic fragments **F** for two PTP, PTP1B and the catalytic domain of SHP2. Fragments active in the ligation assay were subsequently incorporated into peptides and then into non‐peptidic PTP inhibitors to test the suitability of these biomimetic fragments for the development of PTP probes with some degree of specificity and with activity within cells.

## Results and Discussion

**Scheme 1:**
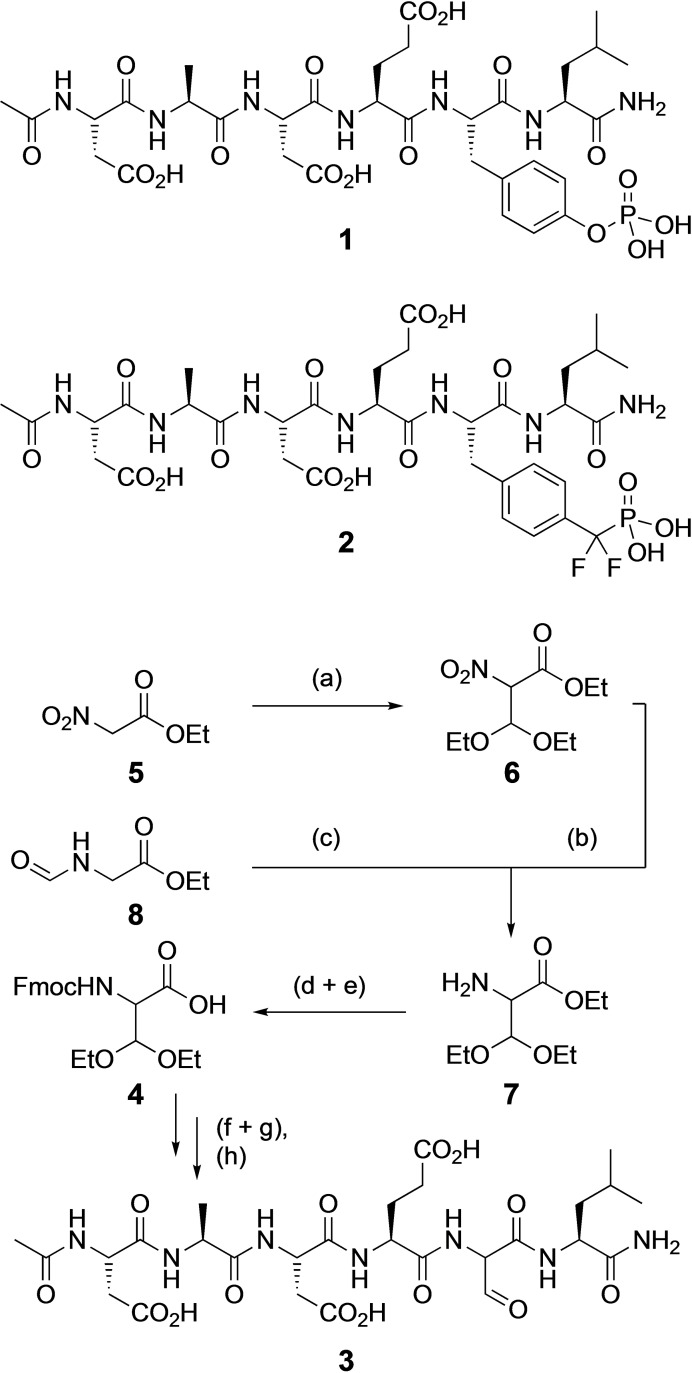
Synthesis of the formylglycine‐bearing hexapeptide **3**. Reaction conditions: a) TiCl_4_, DiPEA, (EtO)_3_CH, −10 °C, DCM, 3 h, 81 %; b) Raney‐nckel, H_2_, EtOH, 12 h, 92 %; c) ethylformiate, tBuOK, Tol, 10 °C, 2 h, HCl (gas), DCM/EtOH, −25 °C, 4 h, K_2_CO_3_, Et_2_O, 47 %; d) LiOH (0.5 M), MeOH, 2 h, e) Fmoc‐OSu, H_2_O/dioxane, 0 °C, 91 % (over 2 steps); peptide synthesis on Rink amide resin: f) 20 % piperidine/DMF; g) Fmoc‐AA‐OH, DIC, HOBT, DMF, repetition of steps f+g n‐times; h) 95 % TFA, 5 % H_2_O, 73 %.

The phospho‐hexapeptide Ac‐Asp‐Ala‐Asp‐Glu‐Tyr*‐Leu‐NH_2_ (Ac‐DADEY*L‐NH_2_) **1** contains the autophosphorylation sequence 988–993 of epidermal growth factor receptor (EGF−R) including the *O*‐phosphorylated tyrosine 992 (Tyr*, Y*) and has been recognized as a substrate of the phosphotyrosine phosphatase PTP1B with a *K_M_* value of 3.6 μM (Scheme 1).^[16]^ Exchange of the phosphotyrosine residue by the established phosphotyrosine mimetic 4‐phosphono‐difluormethyl‐phenylalanine (PDFM−F) furnished a potent inhibitor of PTP1B, Ac‐DADE(PDFM−F)L‐NH_2_ **2** (*K_I_* =1.4 μM, Table 1).^[17]^ Substitution of the phosphotyrosine residue of **1** by 2‐formylglycine resulted in the formylglycine peptide Ac‐DADEfGL‐NH_2_ **3** as our first synthetic target (Scheme 1). For the synthesis of **3**, we prepared the racemic N‐Fmoc‐protected diethylacetal amino acid **4** as a building block. Initially, enantiomerically pure (S)‐**4** was synthesized following a published 7‐step protocol from L‐serine,^[18]^ however, since the peptides obtained after deprotection and cleavage from the resin with acid were identical in the NMR spectra, we decided to use the racemic building block **4** instead and established a facilitated access to **4** in only 3 steps starting from *O*‐ethyl 2‐nitro‐acetate **5**. Treatment of **5** with titanium tetrachloride, *N,N*‐diisopropylethylamine, and triethyl orthoformate afforded the diethyl acetal **6** (conditions a).^[19]^ Hydrogenation of **6** in the presence of Raney nickel furnished ethyl 2‐amino‐3,3‐diethoxypropionate **7**. Alternatively, under conditions b, *N‐*formylglycine ethylester **8** was formylated in C2 position with ethyl formate and tBuOK, followed by acetalization with HCl in EtOH at −25 °C and cleavage of the *N*‐formyl group under basic conditions leading to the same diethylacetal **7**.^[20]^ Saponification of **7** followed by N‐Fmoc protection with Fmoc‐OSu afforded the desired N‐Fmoc‐formylglycine **4** (Fmoc‐fGly, Fmoc‐fG) in a total yield of 68 % over 4 steps (conditions a,b,d,e) or 43 % over 3 steps (conditions c,d,e).

**Table 1:** Inhibition constants of peptide mimetics **1**, **3**, **15**–**18** toward PTP1B and SHP2. Synthesis of peptides **15**–**18** is described in the text.

#	Compound	*K_I_* ^[a]^ [μM] PTP1B	*K_I_* ^[a]^[μM] SHP2
**2**	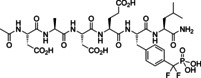	1.4±0.4	120±7
**3**	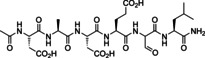	484±30	341±45
**15**	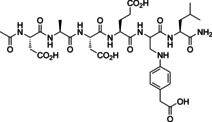	138±21	31±4
**16**	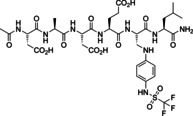	213±21	35±1
**17**	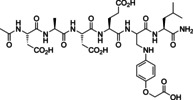	123±16	12±0.5
**18**	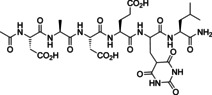	202±21	44±8

[a] Assays were performed in triplicate with DiFMUP as a substrate (see Supporting Information part for raw data). Enzyme concentration was 1.5 nM, substrate concentrations 67 μM (for PTP1B) and 72 μM (for SHP2), respectively, identical with the experimentally determined *K_M_* values of the substrate. *IC_50_* values were converted into the corresponding *K_I_* values applying the Cheng‐Prusoff equation (with [S]=*K_M_* this results in *IC_50_*/2=*K_I_*).

Building block **4** was then used in solid phase peptide synthesis on polystyrene with Rink amide linker by diisopropyl carbodiimide/HOBt activation. Cleavage and complete deprotection was achieved with TFA/H_2_O. (95 : 5 v/v) and the formylglycine peptide **3** was isolated by HPLC in 73 % yield. Peptide **3** was highly soluble in water, buffer, and DMSO, so that we were able to analyze its structure and reactivity in solution (Supporting Information Figures S1–19).

NMR‐spectra of **3** synthesized from (S)‐fGly and rac‐fGly were identical. In DMSO the fGly‐residue appears as a mixture of the aldehyde form **3**, the hydrate form **9**, and the enol form **10**. In H_2_O/D_2_O 9 : 1, exclusively the hydrate form **9** was observed with both diastereomeric Hα at 4.3 ppm and both Hβ at 5.3 ppm. In the DQF‐H,H‐COSY spectrum all backbone NH protons were detectable suggesting that no cyclization with any of the backbone amides occurred and the formyl moiety thus was available for ligation. High resolution Q‐TOF MS analysis further confirmed the predominant hydration of the formylglycine peptide **3**.

**Figure 2:**
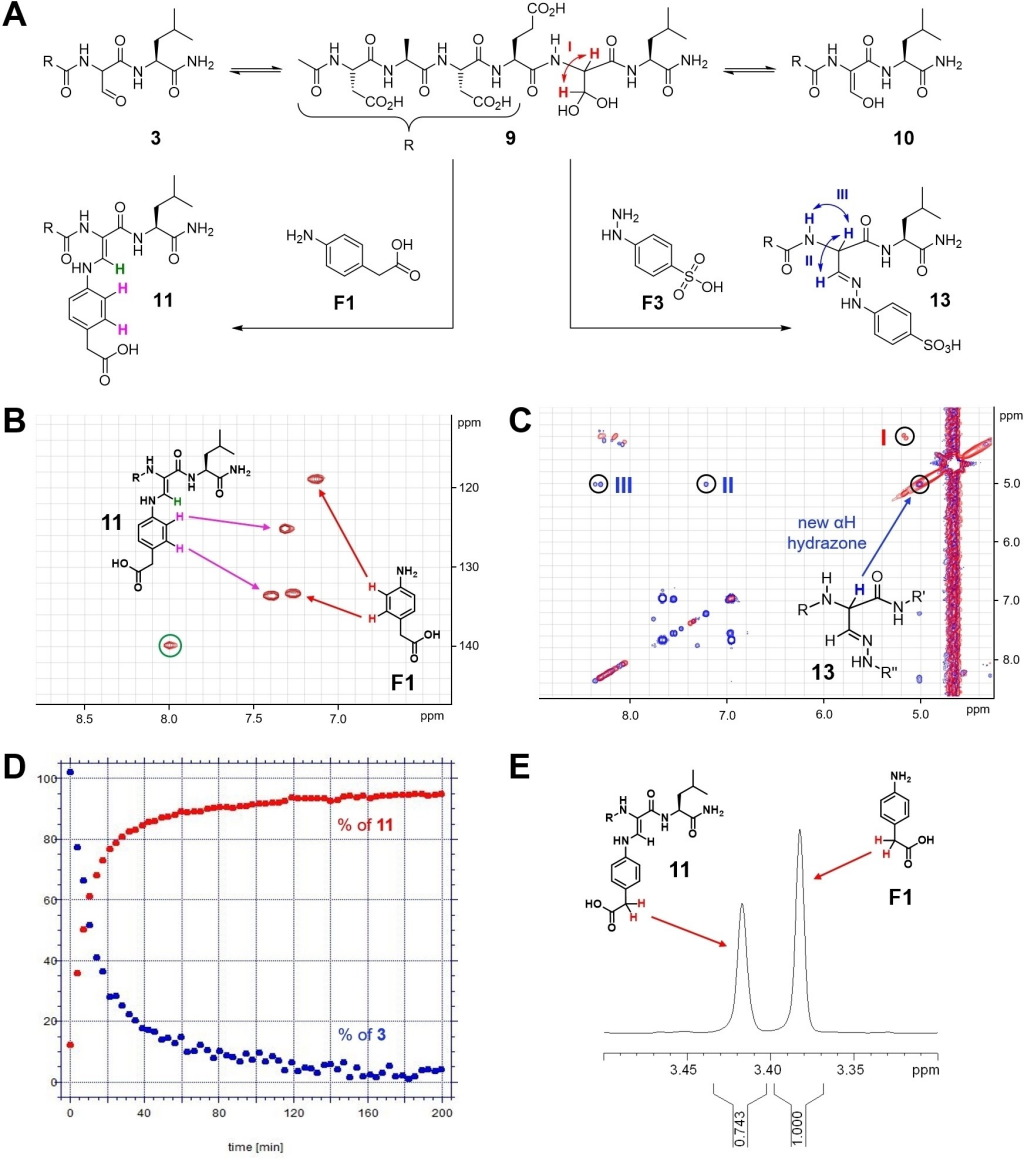
Structures and ligation reactions of formyl‐glycine peptide **3**. A) In water and D_2_O **3** formed exclusively hydrate **9**, in DMSO a mixture of **3**, **9**, and enol **10**. Ligation with arylamine **F1** yielded a single product, Z‐enamine **11**. Ligation with hydrazine **F3** furnished E‐hydrazone **13**. B) Enamine **11** is characterized in the HMQC NMR the cross‐peak (green) between the Cβ of the formylglycine residue and the β‐proton. Z‐configuration was determined by ROESY NMR (Supporting Information Figure S10). C) Hydrazone **13** displayed in H,H‐COSY NMR a retained coupling of Hα with Hβ and with the fGly‐NH. D) Kinetics of the ligation of **3** (5 mM) and **F1** (10 mM) yielding enamine **11** in aqueous buffer (pH 6.5, 50 mM phosphate buffer, 200 mM NaCl) with an average half reaction time of 10.7 min. Quantification of **F1** and **11** via benzylic proton signals in Watergate NMR spectroscopy. E) The ratio of fragment **F1** and enamine **11** was calculated from the integration of the benzyl CH_2_ (red) signals in the ^1^H NMR spectrum.

Ligation reactions of peptide **3** were investigated with several nucleophilic fragments **F** detected in a primary screening experiment (see below) using NMR spectroscopy (Figure 2). Peptide **3** was dissolved with 2 equivalents of 4‐amino‐phenyl‐acetic acid **F1** in H_2_O/D_2_O 9 : 1 resulting in a solution with pH 3 and NMR spectra were recorded. Under these conditions, about 80 % of peptide **3** were converted to the single ligation product, Z‐enamine **11**. In **11**, the Hα of the formylglycine residue disappeared while the Hβ was displayed at 8.0 ppm. ROESY NMR was employed to investigate the stereochemistry of **11** and revealed the formation of a single isomer with Z‐configuration of the double bond and a strong nuclear Overhauser effect between the enamine NH and the adjacent α‐NH of Leu6 (Supporting Information Figure S10). One can suspect that the Z‐configuration of **11** is strongly favored over the E‐configuration due to an H‐bond between the enamine‐NH and the carbonyl residue of Glu4 resulting in a 7‐membered ring (Figure 2A).

The enamine ligation of **3** and **F1** yielding **11** was investigated in aqueous buffer under conditions employed in the enzymatic PTP assay (pH 6.5, 50 mM phosphate buffer, 200 mM NaCl). Formation of the ligation product **11** was followed in Watergate ^1^H NMR for 1000 min by integration of the benzylic CH_2_ group in starting material **F1** and product **11** and indicated product formation with an average half reaction time of 10.7 min (Figure 2D,E).

**Figure 3:**
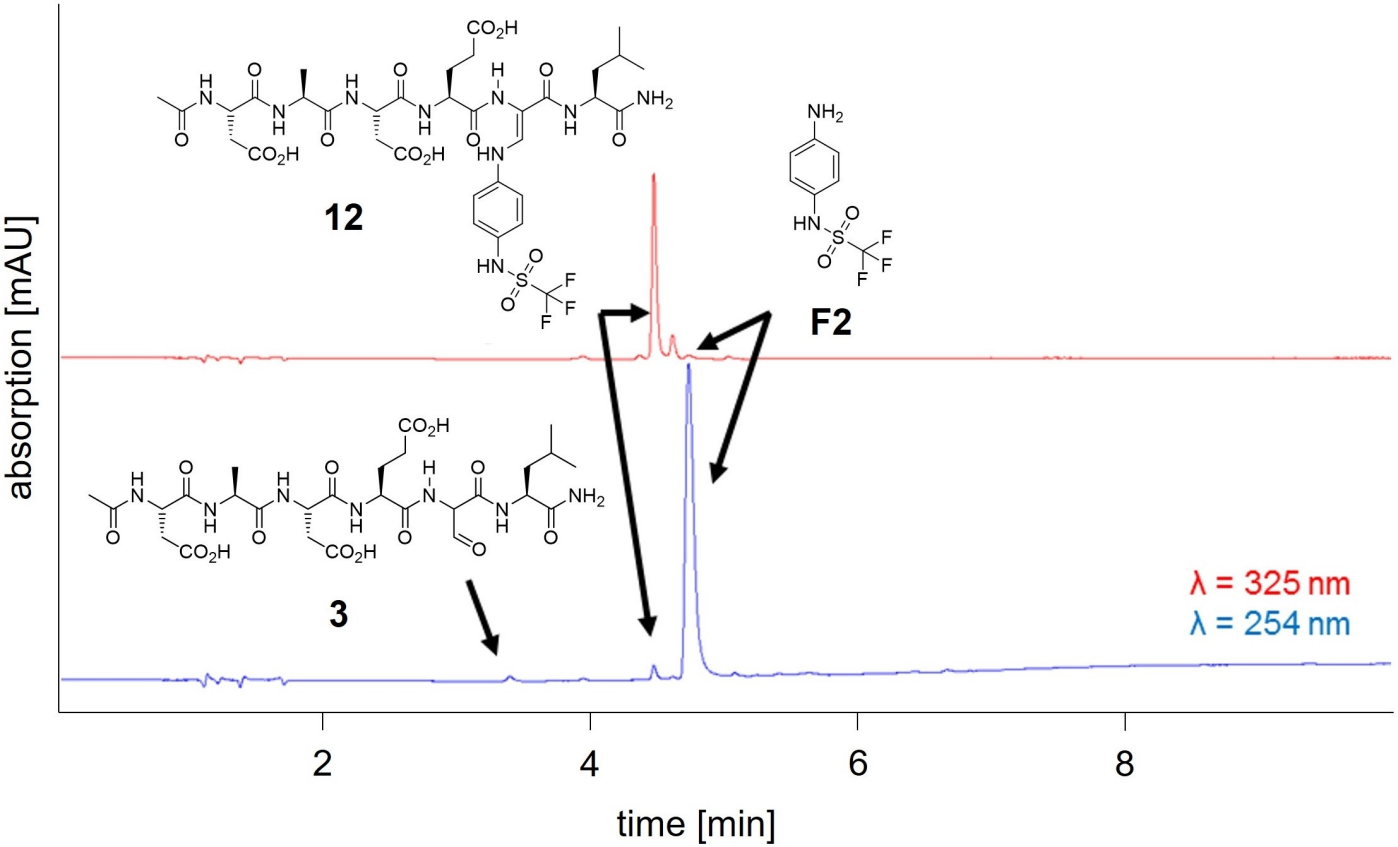
Ligation reaction of formylglycine peptide **3** with N‐(4‐aminophenyl)‐trifluoromethyl sulfonamide **F2** yielding Z‐enamine **12** followed by HPLC‐MS.

Enamines formed from formylglycine peptide **3** through in situ ligation were stable enough to be analyzed by RP‐HPLC‐MS with 0.1 % formic acid at acidic pH 2. For example, the 1 : 2 mixture of **3** and N‐(4‐aminophenyl)‐trifluoromethyl sulfonamide **F2** yielded enamine **12** (Figure 3). In contrast to the reaction with aryl amines, the ligation reaction of **3** with 4‐hydrazo‐phenylsulfonic acid **F3** (1 : 2) formed hydrazone **13** with 100 % conversion of **3** instantaneously (<2 min). **13** was characterized by a shifted Hα of the fGly residue at 5.0 coupling with the Hβ at 7.2 and the fGly‐NH at 8.3 ppm. A single configuration of the hydrazone double bond was formed, presumably the thermodynamically favored E‐isomer. One might suspect as the preferred conformation the one establishing an H‐bonded, 6‐membered ring between the double‐bonded hydrazine‐N1 and the NH of Leu4 (Figure 2C).

Incubation of **3** with an excess of 1,4‐dithio‐threitol (DTT) (1 : 5), a common reducing agent in biochemistry and especially in assays with protein tyrosine phosphatases, yielded a ligation product detectable in HPLC‐MS with a mass corresponding to the hemi‐thioacetal **14** (Supporting Information Figures S20–22). **14** could not be detected in NMR or isolated, which is in full agreement with earlier results on the transient formation of bioactive hemi‐(thio)‐acetals formed by protein‐templated ligation reactions.^[21]^ As a consequence, in ligation experiments with **3** DTT was found to compete with other nucleophiles such as amines or hydrazines. Therefore, it had to be replaced in enzyme assays with a non‐nucleophilic reducing agent. No ligation reaction was observed between formylglycine peptide **3** and tris‐carboxyethyl phosphane (TCEP) which was found to sustain the enzymatic activity of protein tyrosine phosphatases reliably at 50 μM for several hours and therefore was employed in all ligation experiments.

Formylglycine peptide **3** was tested as an inhibitor of two protein tyrosine phosphatases, PTP1B and SHP2, in an enzyme activity assay using DiFMUP (6,8‐di‐fluoro‐4‐methyl‐umbelliferyl phosphate) as a fluorogenic substrate. Peptide **3** bound to and inhibited PTP1B with a *K_I_* value of 484 μM and SHP2 with a *K_I_* of 341 μM (Table 1). Considering the *K_I_* values of peptide **2** for these two proteins, 1.4 μM for PTP1B and 120 μM for SHP2, substitution of the phosphotyrosine mimetic PDFM‐Phe by formylglycine resulted as expected in a significant reduction of inhibitory potency of **3**. At the same time the different affinities of **2** and **3** for PTP1B and SHP2 reflect the structural differences of the two investigated enzymes.

**Figure 4:**
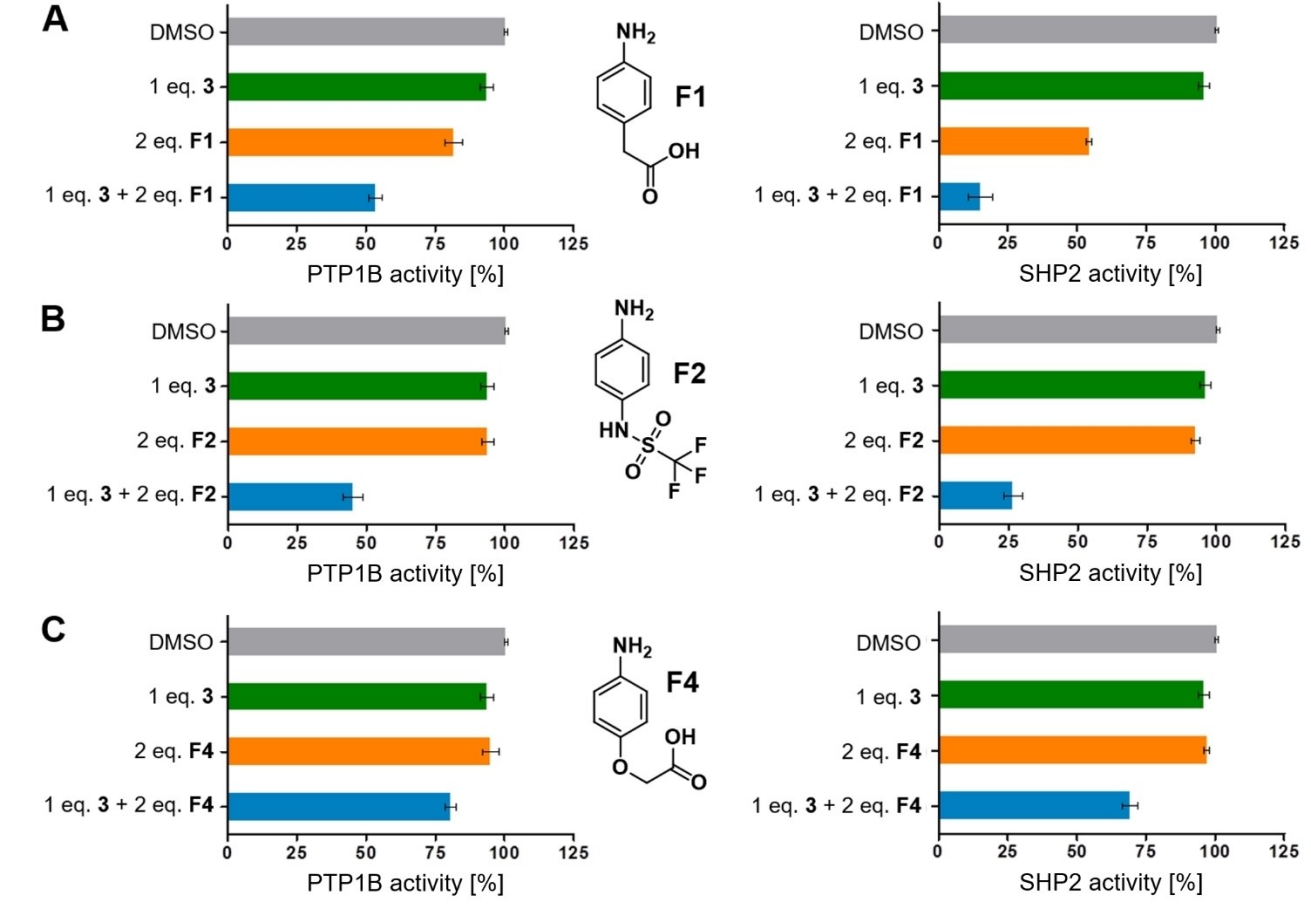
Fragments with over‐additive enhancement of the inhibition of formylglycine peptide **3**. For conditions see experimental section, all experiments were performed in triplicates.

Residual binding of **3** to both proteins and the ability to undergo ligation reactions as described above, encouraged us to conduct fragment ligation screening for phosphotyrosine mimetics using formylglycine peptide **3** as a molecular probe. 95 nucleophilic amine or hydrazine fragments (<250 Da) were selected and tested as inhibitors of PTP1B alone and in combination with peptide **3** (Supporting Information Figures S23–28). Fragments were pre‐selected for library composition based upon representation of potential phosphate‐mimetic substructures including carboxylic acids, sulfonic acids, sulfonamides, and fluorine‐rich functional groups, which were to be investigated for fluorine‐specific interactions with phosphotyrosine binding sites.[^22^, ^23^] Those fragments showing a significant enhancement of inhibition in the combination experiment were considered primary hits and tested in the same fragment ligation experiment with SHP2 (Figure 4).

While the negative control fragment aniline was entirely inactive, 4‐amino‐phenylacetic acid **F1**, 4‐amino‐phenoxyacetic acid **F4**, and N‐(4‐aminophenyl)‐trifluoromethyl sulfonamide **F2** displayed over‐additive inhibition in the ligation experiments, both for PTP1B and for SHP2. In contrast, the corresponding amino fragments with direct attachment of the acidic head group to the benzene ring, such as 4‐amino benzoic acid **F5**, N‐(4‐aminophenyl)‐sulfonic acid **F6**, were inactive. Apparently, the insertion of 1–2 atoms between the aromatic ring and the head group was essential for activity.

Among the fluorine‐containing fragments 4‐amino‐phenyl‐pentafluorosulfate **F7**, 4‐amino‐phenyl‐trifluoromethane **F8** and 4‐piperidinyl‐difluoromethyl phosphonate **F9** were inactive. Ligation of one of the inactive fragments, **F5**, with **3** was investigated in buffer by ^1^H‐ and HMQC NMR showing formation of the enamine ligation product and confirming the inactivity of this enamine in the assay (Supporting Information Figures S17–19). The only fluorine‐rich fragment with pronounced activity in the ligation assay was N‐(4‐aminophenyl)‐trifluoromethyl sulfonamide **F2**.

**Figure 5:**
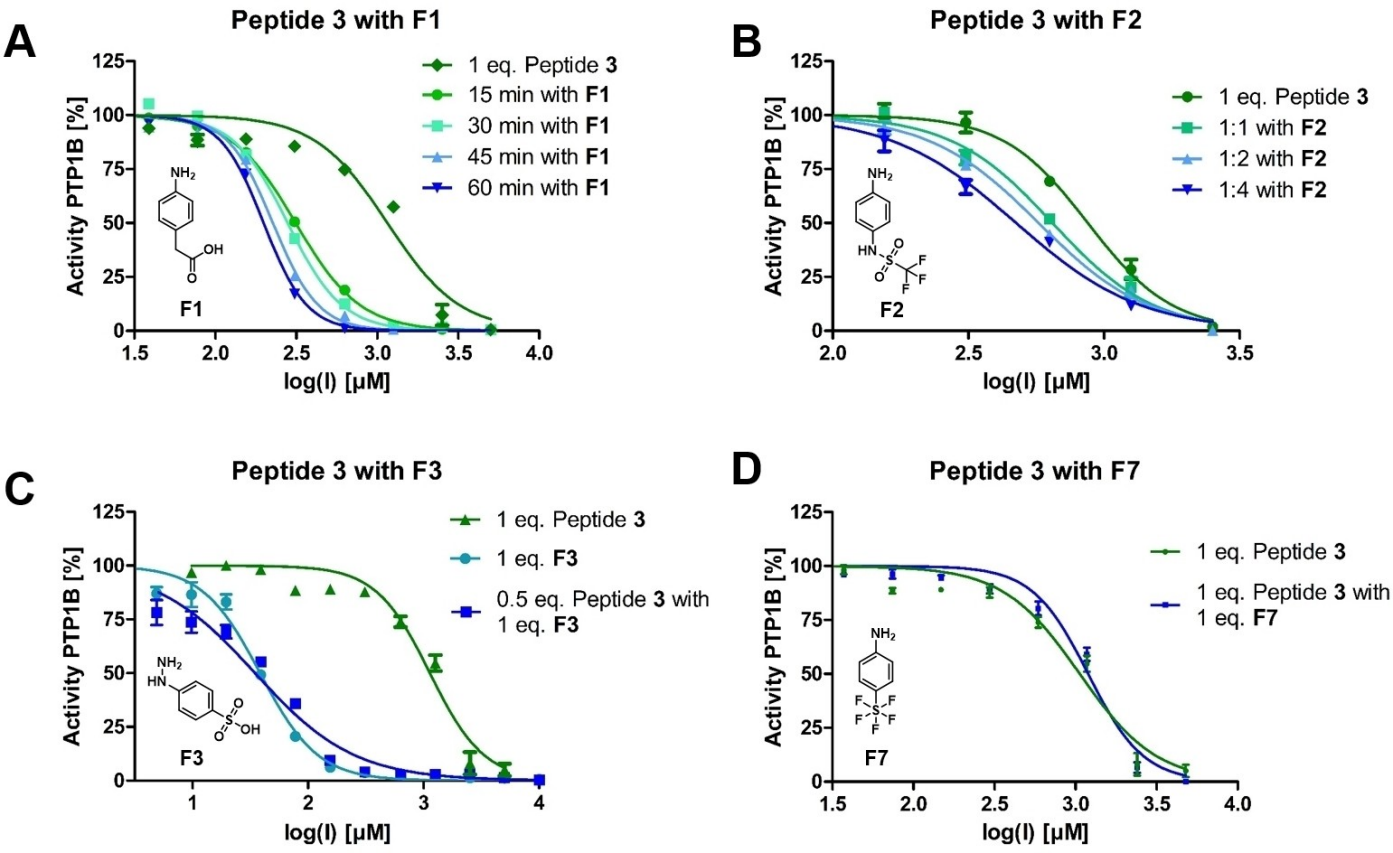
Inhibition of PTP1B with concentration‐ and time‐dependent ligation of formylglycine peptide **3** with fragments. A) Time‐dependent ligation of **3** with 4‐amino‐phenyl acetic acid **F1**. B) Active fragment ligation product of N‐(4‐aminophenyl)‐trifluoromethyl sulfonamide **F2** with **3** with an apparent *K_I_* of 17 μM. C) Inhibition by the active fragment 4‐hydrazo‐phenylsulfonic acid **F3** with and without **3**. D) Inactive fragment ligation product of 4‐amino‐phenyl‐pentafluoro‐sulfate **F7**.

For a more precise, quantitative understanding of the fragment ligation experiments with formylglycine peptide **3**, inhibition experiments were conducted at different concentrations (Figure 5). The active phosphotyrosine‐mimetic fragment **F2** shifted the sigmoidal inhibition curves with increasing substrate excess yielding apparent *K_I_* values of the fragment ligation product (Figures 5B). In agreement with the NMR studies reported above, formation of and inhibition by enamine **11** was time‐dependent with an improvement of the apparent *K_I_* ‐value from 157 to 99 μM from 15 to 60 min incubation (Figure 5A). Formation of enamine ligation product **12** could be forced by increasing the concentration of fragment **F2** in the assay. With 30 min of incubation, the apparent *K_I_*‐value was raised from 322 μM (1 : 1 ratio of **F2** and **3**) to 237 μM (4 : 1 ratio) (Figure 5B). 4‐Hydrazo‐phenylsulfonic acid **F3** was another special case of hit fragments. **F3** was found to inhibit PTP1B with a *K_I_* of 17 μM without addition of the formylglycine peptide **3** (Figure 5C). Formation of the hydrazone did not enforce inhibition of PTP1B further. Modified hydrazines showed even further improved inhibition (Supporting Information Table S1). In the case of inactive fragments such as the 4‐amino‐phenyl‐pentafluorosulfate **F7**, formylglycine peptide **3** displayed identical inhibition curves with or with the added fragment (Figure 5D).

Inhibition of PTP1B by Z‐enamines such as **11** and **12** raised the question, whether also peptides derived from enamines through reduction of the double bond would act as inhibitors or whether they would lose activity. Reductive amination of formylglycine peptide **3** with aryl amines **F1** or **F4**, respectively, in dry MeOH with molecular sieves furnished peptides **15** and **16**, respectively, using NaCNBH_3_ as reductant. The same protocol could be used to prepare the reduced Knoevenagel product **17** from peptide **3** and barbituric acid as a *C*‐nucleophile. Synthesis of the triflyl‐containing peptide **18** required the preparation of the chiral L‐2‐*N*‐Fmoc‐protected 3‐*N*‐(4‐trifluoromethyl‐sulfonylamido‐phenyl)‐2,3‐diamino‐propanoic acid **19**, which was obtained in three steps from chiral L‐2‐*N*‐Boc‐2,3‐diamino‐propanoic acid via 3‐(4‐trifluoromethyl‐sulfonamido‐phenyl)‐2,3‐diamino‐propanoic acid **20**. Building block **19** was employed in peptide synthesis to provide **18**.

Peptides **15**–**18** were subsequently tested as inhibitors of PTP1B and of SHP2 (Table 1). All peptides were active inhibitors of PTP1B in the same range or slightly better than the enamines tested before. Inhibition of SHP2 was remarkably stronger in all four cases than inhibition of PTP1B.

**Figure 6:**
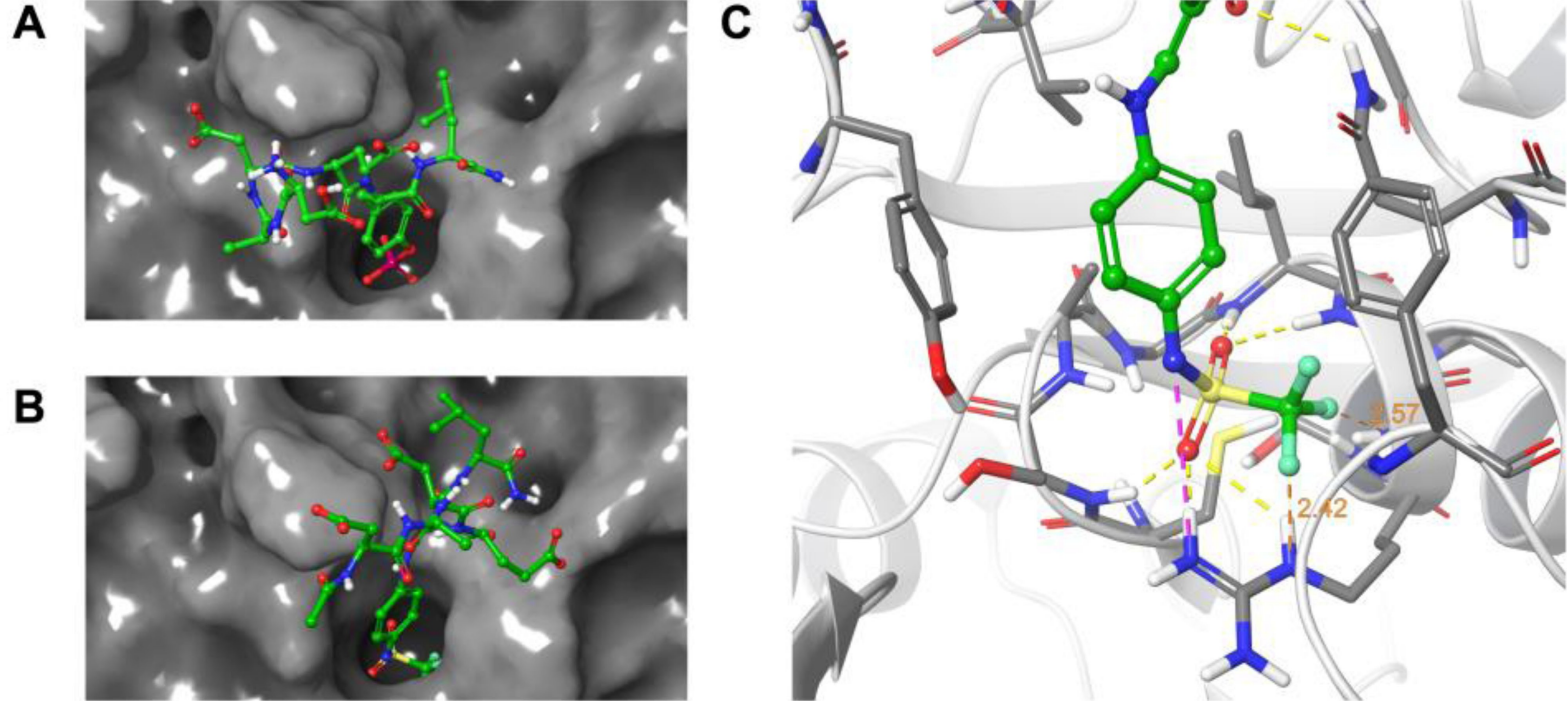
Docking studies. A. Native phosphotyrosine peptide **1** re‐docked in the binding site of modified PTP1B from crystal structure 1PTU.^[24]^ B. Docking pose of *Z*‐enamine **12** (ligation product) in the active site. C. Close‐up of the phosphotyrosine binding pocket hosting the *N*‐(4‐aminophenyl)‐trifluoromethylsulfonamide residue of **12**. H‐bonds are indicated in yellow, salt bridges in magenta, distances in orange. Ligand C‐atoms in green, protein C‐atoms in grey, N in blue, F in light green.

The GLIDE protocol^[25]^ was used to dock formylglycine peptide **3**, hydrate **9**, Z‐enamines **11** and **12**, and the reduced peptides **15**, **16**, and **18** to the phosphotyrosine peptide binding site of PTP1B. As a starting point, the crystal structure of the native phosphotyrosine peptide **1** with PTP1B (pdb: 1PTU) was employed.^[24]^ In all docking runs, only the general binding site region was specified, with no positional restraints on the docking poses. As a result, the position and orientation of the backbone of the docked peptides varied significantly between all obtained docking poses (Supporting Information Figure S32, top). This variation reflected the great number of positional degrees of freedom of peptides, making random docking of peptides and the scoring of docking poses a challenging task. In the docking poses of peptides **3** and **9**, the formylglycine residue, as aldehyde or hydrate, was consistently oriented toward the solvent and the glutamate residue (Glu4) was placed into the phosphotyrosine binding pocket, with the carboxylate anion of Glu4 interacting with the backbone amides and the side chain of Arg221 (Supporting Information Figure S32, bottom). In contrast, the enamine ligation products **11**, **12**, and their reduction products **15**, **18**, docked reliably the phosphotyrosine‐mimetic fragment into in the phosphotyrosine binding pocket of PTP1B, very similar to the phosphate of natural ligand **1** (Figure 6, Supporting Information Figure S33). In the preferred docking poses of **11** and **12**, the headgroups of the phosphotyrosine mimetic fragments **F1** and **F2**, carrying a negative charge as carboxylate and sulfonylamido anions, respectively, were placed in the phosphate binding loop constructed from residues Cys215‐Arg221. The anionic fragments interacted with the backbone amide‐NH groups carrying positive partial charges, as well as with the protonated Arg221 sidechain, thereby replacing the phosphate headgroup of the natural ligand. Sulfonyl oxygen and fluorine atoms of **11** were found in proximity (<3 Å) to several of the backbone nitrogens suggesting N−H−F hydrogen bond interactions (Figure 1C). In docking experiments of the peptides **15**, **16**, and **18** to SHP2 with the same technical specifications as for PTP1B, the obtained docking poses placed either the phosphotyrosine‐mimetic fragment or one of the carboxylate residues into the main phosphotyrosine binding pocket. Analysis of the binding site regions of PTP1B and SHP2 showed that the phosphotyrosine binding site of SHP2 is broad and shallow with a surface displaying a more positive electrostatic potential compared to the deep and narrow phosphotyrosine binding pocket of PTP1B with lower positive potential (Supporting Information Figure S34). These observations might explain that the peptides carrying four negative charges bind with higher affinity and with higher structural diversity to SHP2 than to PTP1B, while the deeper pocket of PTP1B is more specific for binding of the phosphotyrosine mimetic fragment. In conclusion, the docking studies confirmed the suitability of **F1**, **F2**, and **F4** as phosphotyrosine‐mimetic fragments, suggesting significant charge and hydrogen bond interactions of fragment ligation products with the protein binding pocket of PTP1B and SHP2.

**Figure 7:**
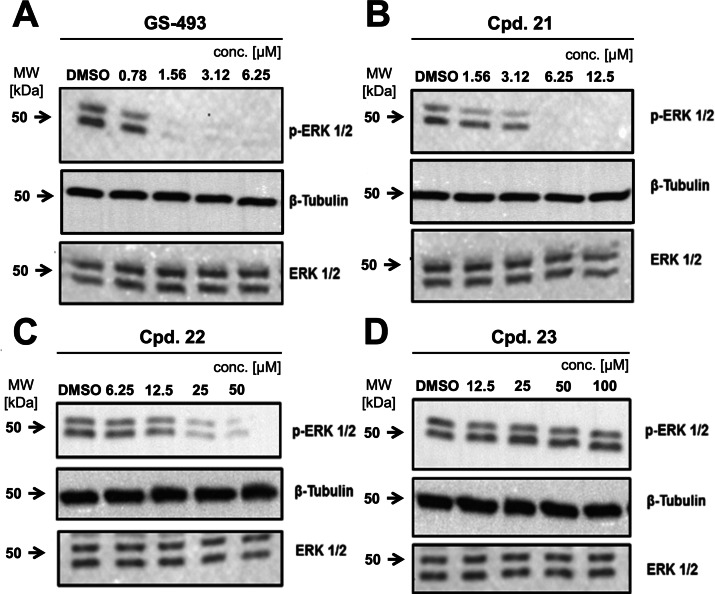
Cell experiments. Compounds **GS‐493**, **21** and **22** decreased the HGF‐induced tyrosine phosphorylation of ERK 1/2; Western blot analysis of p‐ERK and total ERK in HeLa cells after 1 h treatment with compound **GS‐493** (A), **21** (B), **22** (C) and **23** (D); DMSO treatment as control; beta‐tubulin was used as a control for uniform protein loading.

Our next goal was to validate the three phosphotyrosine mimetics **F1**, **F2**, and **F4** identified by fragment ligation screening as substructures of heterocyclic and cell‐penetrating SHP2‐inhibitors. The potent SHP2‐specific inhibitor **GS‐493** was selected as a reference compound. **GS‐493** inhibited the catalytic domain of SHP2 with a *K_I_*‐value of 36 nM and was able to block SHP2 signaling in cancer cells as well as in animal models.^[26]^ Most remarkably, **GS‐493** was able to block the development of mammary gland tumors in an endogeneous cancer model.^[27]^ In combination with a kinase inhibitor, **GS‐493** was able to suppress pancreas cancer.^[28]^ In **GS‐493**, the phenyl sulfonic acid serves as phosphotyrosine mimetic. Sulfonic acids are not considered as drug‐like chemical entities due to low oral bioavailability.^[29]^ Thus, replacement of the sulfonic acid residue in **GS‐493** with an alternative phosphotyrosine mimetic was desirable. Pyrazolones **21**–**23** were prepared from the aromatic amines **F1**, **F2**, and **F4** via diazotation, followed by azo‐coupling of the in situ generated diazonium salt to 2,5‐bis‐(4‐nitro‐phenyl)‐2,4‐dihydro‐3*H*‐pyrazol‐3‐ones (Table 2).^[30]^ Pyrazolones **21** and **23** inhibited SHP2 with *K_I_* values of 79 nM and 275 nM, respectively, in the same range as **GS‐493**, whereas the affinity of **22** for SHP2 was lower (5.9 μM). For compound **21**, the *K_I_* value was determined using Michaelis‐Menten kinetics as well, yielding 32 nM, and indicating a competitive mode of inhibition (Supporting Information Figure S31 and Supporting Information Table S2). Docking of pyrazolones **GS‐493** and **21**–**23** to SHP2 was conducted placing the phosphotyrosine mimetics reliably into the phosphotyrosine binding pocket of the enzyme (see Supporting Information Figure S35). Finally, the cellular activity of pyrazolones **21**–**23** was investigated in HeLa cells (Figure 7). Inhibition of SHP2 has been reported to activate Sprouty a potent inhibitor of Ras, shutting down effectively the Raf‐Erk‐Map signaling pathway.^[31]^ Thusfore, the dephosphorylation of Erk can be monitored as a downstream event following to inhibition of SHP2. For control, cells were treated with **GS‐493** resulting in the complete dephosphorylation of Erk already at 1.6 μM. Pyrazolone **21** carrying the phenyl acetic acid residue was an efficient cellular inhibitor of SHP2 at 6.25 μM with complete dephosphorylation of pErk. Triflyl‐amide **22** inhibited Erk‐phosphorylation effectively at 25 μM while the phenoxy‐acetic acid **23** did not inhibit SHP2 in cells at concentrations up to 100 μM. In summary, phenylacetic acid and *N‐*phenyl‐trifluoromethyl sulfonamide were demonstrated to be cellular effective phosphotyrosine‐mimetic fragments.

**Table 2:** Inhibition of SHP2 by pyrazolones carrying the novel phosphotyrosine mimetics.

#	Compounds^[c]^	*K_I_* [μM] SHP2
**GS‐493**	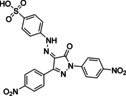	0.036^[a]^ ±0.002
**21**	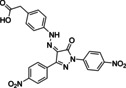	0.079^[a]^ ±0.023 0.032^[b]^ ±0.015
**22**	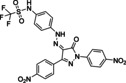	5.9^[a]^ ±0.56
**23**	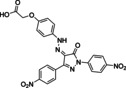	0.224^[a]^ ±0.029

[a] Assays were performed in triplicate with DiFMUP as a substrate (see Supporting Information part for raw data). Enzyme concentration was 1.5 nM, substrate concentration 72 μM, identical with the experimentally determined *K_M_* value of the substrate. *IC_50_* values were converted into the corresponding *K_I_* values applying the Cheng‐Prusoff equation (with [S]=*K_M_* this results in *IC_50_*/2=*K_I_*). [b] *K_I_* value determined by using Michaelis‐Menten kinetics at different substrate concentrations as described in the Supporting Information, the inhibition mode was found to be competitive.

## Conclusion

We have demonstrated the use of formylglycine peptide **3** for site‐directed fragment ligation screening. A simplified protocol for the preparation of formylglycine peptides was developed based on a 3‐step‐synthesis of the Fmoc‐protected glycine acetal **4**. In solution, formylglycine peptide **3** was found to be stable in extended topology, displaying a dynamic equilibrium between the aldehyde, hydrate, and enol structures. Reactions with nucleophiles furnished fragment ligation products in aqueous buffer at physiological pH as studied by NMR spectroscopy and HPLC‐MS. Although the ligation of thiols provided hemi‐thioacetals with limited stability, amines and hydrazines gave stable ligation products with precisely defined stereochemistry and fast ligation kinetics, namely, *Z*‐enamines and *E*‐hydrazones, respectively. As a result, formylglycine peptide **3** enabled fragment ligation screening for phosphotyrosine mimetics employing the enzymes PTP1B and SHP2. Several fragments were identified and validated as hits. Fragment **F1**, **F2**, and **F4** displayed super‐additive inhibition of PTP1B and, much more pronounced, of SHP2 in the presence of formylglycine peptide **3**. The observed effects were very sensitive to subtle changes of the fragment structures as indicated by inactive control fragments. Apparent *K_I_* values of ligation products **11** and **12** were reduced in comparison with peptide **3** and were similar to the inhibition constants of the reduced peptides **15**–**18**. Random docking of the *Z*‐enamine ligation products **11**, **12** and of reduction products **15**, **16**, and **18** consistently placed the fragments **F1**, **F2**, and **F4** into the phosphotyrosine binding pocket of PTP1B, exerting Coulomb interactions, O−H−N, and F−H−N hydrogen bonds with the phosphate binding loop of the protein. Validated phosphotyrosine‐mimetic fragments **F1**, **F2**, and **F4** were incorporated into the pyrazolone scaffold of the SHP2‐specific inhibitor **GS493** and yielded nanomolar inhibitors of SHP2 that were able to block the Raf‐Erk‐Map signaling pathway in HGF‐activated cancer cells.

In summary, we have established a formylglycine peptide as a powerful screening tool to identify fragments binding to a precisely defined binding pocket for a single amino acid residue. In principle, this strategy should be broadly applicable to peptide‐protein and protein‐protein‐interactions, that are defined by binding sites specific for a natively encoded or post‐translationally modified amino acid residue. Such binding sites are, for example, found in proteases, phosphopeptide binding domains, or N‐acetyl lysine binding domains, just to name a few prominent examples.

## Experimental Section

**N‐Acetyl‐L‐aspartyl‐L‐alaninyl‐L‐aspartyl‐L‐glutamyl‐2‐formylglycyl‐L‐leucinyl‐amide (Ac‐DADE‐fG‐L‐NH_2_) 3**: Peptide synthesis was conducted on Rink amide resin using Fmoc‐strategy. PP/PE syringes equipped with a PE‐frit were used as reaction vessels. Amino acids (Asp, Ala, Glu, Leu, and the unnatural amino acid **4**) were coupled with *N*,*N*‐diisopropylcarbodiimide (DIC) and *N*‐hydroxybenzotriazole (HOBt) in a minimal volume of DMF. Five equivalents (with respect to the loading of the resin) of Fmoc‐amino acid were preactivated with DIC/HOBt for 5 min, added to the resin and shaken for 3 h, followed by a washing step with DMF. The coupling reactions were monitored using the Kaiser test. The Fmoc‐group was cleaved by adding a solution of piperidine/DMF (20 : 80 *v*/*v*) twice for 10 min followed by washing with DMF. Before cleavage of peptide from the resin, resin loading was quantified via UV‐photometric determination of the dibenzofulvene product following to Fmoc cleavage from a weighted resin sample. For final N‐acetylation, a solution of acetic anhydride in DCM/DIPEA/Ac_2_O (80 : 10 : 10 *v*/*v*) was added to the resin and shaken for 15 min. Before cleavage, the resin was washed with 5 syringe volumes of DMF, THF and DCM followed by drying in vacuo. 400 mg of preloaded resin (0.4 mmol/g) were used for cleavage. The product was cleaved with TFA/H_2_O (95 : 5 *v*/*v*) for 3 h, peptide **3** was precipitated in cold diethyl ether and was purified by preparative reversed phase HPLC yielding 58.6 mg (73 %) of a white solid after lyophilization. ^**1**^ **H NMR**: (600 MHz, D_2_O): δ=8.44 (1H, d, *J*=10.2 Hz, Leu6 NH), 8.41 (1H, d, *J*=12.5 Hz, Ala2 NH), 8.37 (1H, d, *J*=7.3 Hz, Asp1 NH), 8.34 (1H, d, *J*=7.3 Hz, Asp3 NH), 8.28 (1H, dd, *J*=13.2, 6.8 Hz, fGly5 NH), 8.18 (1H, dd, *J*=7.4, 3.3 Hz, Glu4 NH), 7.49 (1H, d, *J*=22.1 Hz, NH_2_), 7.08 (1H, d, *J*=15.3 Hz, NH_2_), 5.29 (1H, dd, *J*=12.9, 5.5 Hz, fGly5 Hβ as diol), 4.45–4.40 (1H, m, Leu6 Hα), 4.38–4.32 (1H, d, *J*=6.2 Hz, Ala2 Hα), 4.34–4.25 (3H, m, *J*=6.3 Hz, Asp1/Asp3/Glu4 Hα), 2.95–2.83 (4H, m, Asp1/Asp3 Hβ), 2.51–2.41 (2H, m, Glu4 Hγ), 2.17–2.13 (1H, m, Glu4 Hβ), 2.05–2.0 (3H, s, Ac CH_3_), 2.0–1.96 (1H, m, Glu4 Hβ), 1.69–1.61 (3H, m, Leu6 Hβ/Hγ), 1.38 (3H, d, *J*=7.2 Hz, Ala2 Hβ), 0.92 (3H, d, *J*=5.6 Hz, Leu6 Hδ), 0.85 (3H, d, *J*=5.0 Hz, Leu6 Hδ) ppm. **HRMS**: (ESI): C_27_H_41_N_7_O_14_ [M], 687.2711 Da. calcd *m/z* 688.2790 [M+H]^+^, 710.2609 [M+Na]^+^, found *m/z* 688.2792 [M+H]^+^, 710.2607 [M+Na]^+^.

**3,3‐Diethoxy‐2‐(9H‐fluoren‐9‐ylmethoxycarbonylamino)‐ propanoic acid 4**: Ethyl 2‐amino‐3,3‐diethoxypropanoate **7** (500 mg, 2.44 mmol, 1 equiv) was suspended in 0.5 M aq. LiOH solution (5 mL) and methanol was added until a clear solution remained. It was stirred at room temperature for 2 h. TLC control after this time revealed the hydrolysis of **7** after which the mixture was neutralized with Amberlite^®^ IR‐120 loaded with H^+^. The resin was filtered off and washed with water and concentrated under reduced pressure. The product of hydrolysis and NaHCO_3_ (512 mg, 6,09 mmol, 2.5 equiv) were stirred in H_2_O (10 mL) at 0 °C. To this solution was added dropwise 9‐fluorenylmethyl *N*‐succinimidyl carbonate (905 mg, 2.682 mmol, 1.1 equiv) dissolved in 1,4‐dioxane (10 mL) at 0 °C. The mixture was stirred for 1 h at 0 °C, warmed to room temperature and then stirred for another 12 h. The reaction mixture was concentrated under reduced pressure and purified by column chromatography to provide **4** as white solid (891.9 mg, 91 %); *R*_f_=0,46 (dichloromethane/methanol, 6 : 1). ^**1**^ **H NMR**: (500 MHz, CDCl_3_): δ=7.75 (2H, d, *J*=7.5 Hz, Ar−H), 7.61 (2H, d, *J*=7.5 Hz, Ar−H), 7.39 (2H, t, *J*=7.5 Hz, Ar−H), 7.31 (2H, t, *J*=7.5 Hz, Ar−H), 5.67 (1H, d, *J*=8.3 Hz, NH), 4.85 (1H, d, *J*=2.7 Hz, Hβ), 4.59 (1H, d, *J*=8.3 Hz, Hα), 4.39 (2H, d, *J*=7.3 Hz, Fmoc C*H*_2_), 4.24 (1H, t, *J*=7.3 Hz, Fmoc C*H*), 3.88–3.77 (1H, m, C*H*_2_CH_3_), 3.76–3.6 (1H, m, C*H*_2_CH_3_), 3.65–3.57 (2H, m, C*H*_2_CH_3_), 1.32 (3H, t, *J*=6.3 Hz, CH_2_C*H*_3_), 1.21 (3H, t, *J*=6.3 Hz, CH_2_C*H*_3_) ppm. ^**13**^ **C NMR**: (126 MHz, CDCl_3_): δ=172.33 (*C*OOH), 156.59 (*C*ONH), 143.80 (Ar−C_quart_), 141.37 (Ar−C_quart_), 127.83 (Ar−C), 127.19 (Ar−C), 125.28 (Ar−C), 120.06 (Ar−C), 101.17 (Cβ), 67.53 (Fmoc C*H*_2_), 64.74 (*C*H_2_CH_3_), 64.69 (*C*H_2_CH_3_), 56.73 (Cα), 47.15 (Fmoc C*H*), 15.20 (CH_2_ *C*H_3_), 15.08 (CH_2_ *C*H_3_) ppm. **HRMS**: (ESI): C_22_H_25_NO_6_ [M], 399.1682 Da. calcd *m/z* 398.1604 [M−H]^−^, 422.158 [M+Na]^+^, found *m/z* 398.1609 [M+H]^+^, 422.1578 [M+Na]^+^.

**Ethyl 3,3‐diethoxy‐2‐nitropropanoate 6**: To a solution of ethyl‐nitroacetate **5** (1.0 g, 7.51 mmol, 1 equiv) in anhydrous methylene dichloride (25 mL) held at ‐ 10 °C under an argon atmosphere was slowly added by syringe titanium(IV)‐chloride (0.989 mL, 9.01 mmol, 1.2 equiv). The mixture was stirred for 10 min and *N,N*‐diisopropylethylamine (1.53 mL, 9.01 mmol, 1.2 equiv) was added to the mixture dropwise over 30 min. The Mixture was held at −10 °C with stirring for 1 h. triethyl‐orthoformate (3.71 mL, 22.54 mmol, 3 equiv) was added to the mixture dropwise and stirring was continued for 2 h at −10 °C. The reaction mixture was diluted with a 20 % solution of ethanol in saturated aqueous NaHCO_3_ (100 mL) and the mixture was stirred vigorously for 10 min. Organic solvents were removed from the mixture under reduced pressure. Water (200 mL) was added to the concentrated reaction mixture and the aqueous phase was extracted three times with ethylacetate. The combined organic phases were filtered over celite and dried over Na_2_SO_4_. Evaporation of the solvents under reduced pressure afforded compound **6** as yellow oil (1.421 g, 81 %); *R*_f_=0,46 (hexane/ethyl acetate, 9 : 1). ^**1**^ **H NMR**: (500 MHz, CDCl_3_): δ=5.24 (1H, d, J=8.0 Hz, Hα), 5.17 (1H, d, J=8.0 Hz, Hβ), 4.28 (2H, q, J=7.1 Hz, COOC*H*_2_CH_3_), 3.81–3.75 (2H, m, OC*H*_2_CH_3_), 3.69–3.61 (2H, m, OC*H*_2_CH_3_), 1.30 (3H, t, J=7.1 Hz, COOCH_2_C*H*_3_), 1.19 (6H, dt„ J=7.1 Hz, OCH_2_C*H*_3_) ppm. ^**13**^ **C NMR**: (126 MHz, CDCl_3_): δ=161.77 (COOEt), 100.08 (Cβ), 88.52 (Cα), 64.79 (O*C*H_2_CH_3_), 64.60 (O*C*H_2_CH_3_), 63.18 (COO*C*H_2_CH_3_), 15.16 (OCH_2_ *C*H_3_), 15.07 (OCH_2_ *C*H_3_), 13.94 (COOCH_2_ *C*H_3_) ppm. **HRMS**: C_9_H_17_NO_6_ [M], 235.1056 Da. calcd *m/z* 258.0948 [M+Na]^+^, 274.0687 [M+K]^+^, found *m/z* 258.0948 [M+Na]^+^, 274.0680 [M+K]^+^.

**Ethyl 2‐amino‐3,3‐diethoxypropanoate 7 (Method A)**: Ethyl 3,3‐diethoxy‐2‐nitropropanoate **6** (150 mg, 0.638 mmol) was dissolved in absolute ethanol (5 mL) and hydrogen was bubbled through the mixture for 5 min. raney nickel was added and the mixture was hydrogenated at atmospheric pressure for 12 h at room temperature. The reaction mixture was filtered through celite, and the filter cake was washed plentiful with ethanol. Filtrate was concentrated under reduced pressure, leaving a reddish oil. Vacuum distillation of this afforded **7** as yellowish oil (119.1 mg, 92 %); *R*_f_=0,29 (hexane/ethyl acetate, 1 : 1). ^**1**^ **H NMR**: (500 MHz, CDCl_3_): δ=4.61 (1H, d, J=4.5 Hz, Hβ), 4.24–4.16 (2H, m, J=7.1 Hz, COOC_2_CH_3_), 3.77–3.67 (2H, m, OC_2_CH_3_), 3.61 (1H, d, J=4.5 Hz, Hα), 3.58–3.51 (2H, m, OC_2_CH_3_), 2.05–1.95 (2H, br s, NH_2_) 1.30–1.25 (3H, t, J=7.1 Hz, COOC_2_C*H*_3_), 1.23–1.19 (3H, m, OC_2_C*H*_3_), 1.19–1.15 (3H, m, OC_2_C*H*_3_) ppm. ^**13**^ **C NMR**: (126 MHz, CDCl_3_): δ=161.8 (*C*OOEt), 100.1 (Cβ), 88.5 (Cα), 64.8 (O*C*_2_CH_3_), 64.60 (O*C*_2_CH_3_), 63.2 (COO*C*_2_CH_3_), 15.2 (OC_2_ *C*H_3_), 15.1 (OC_2_ *C*H_3_), 13.9 (COOC_2_ *C*H_3_) ppm. **HRMS**: (ESI): C_9_H_19_NO_4_ [M], 205.1314 Da. calcd *m/z* 206.1392 [M+H]^+^, 228.1206 [M+Na]^+^, found *m/z* 206.1379 [M+H]^+^, 228.1195 [M+Na]^+^.

**Ethyl 2‐amino‐3,3‐diethoxypropanoate 7 (Method B)**: A solution of *N*‐formylglycine ethylester **8** (5000 mg, 38.13 mmol, 1 equiv) in ethylformate (15.4 mL, 190.65 mmol, 5 equiv) was added dropwise to a mixture of potassium *tert*‐butoxide (5135 mg, 45.74 mmol, 1.2 equiv) in toluene (60 mL) at 10 °C over a periode of 2 h. Stirring was continued for additional 2 h after which the mixture was allowed to stand at 4 °C for 18 h. The supernatant was discarded and the gelantinious residue dissolved in ethanol (35 mL). This solution was the diluted with methylene dichloride (50 mL) and cooled to −25 °C and treated with HCl gas for 3 h. The mixture was then stirred for 24 h at room temperature. The solution was concentrated under reduced pressure and the residue was suspended in diethyl ether (80 mL). This suspension was treated with saturated K_2_CO_3_ until strongly basic when the phases were separated, and the organic phase was washed further with water and dried over Na_2_SO_4_ and evaporated under reduced pressure to afford an oil. Vacuum distillation of this afforded **8** as yellowish oil (3685 mg, 47 %).

**NMR ligation experiments**: The ligation of peptide **3** with fragments **F1**, **F2** or **F3** was confirmed by NMR experiments. The respective fragments were dissolved in a solution of **3**, to yield a sample with a final concentration of 10 mM of the fragment and 5 mM of **3**. Experiments were performed in 9 : 1 H_2_O/D_2_O or buffer (9 : 1 H_2_O/D_2_O, pH 6.5, 50 mM sodium phosphate, 200 mM NaCl) at 300 K. Experiments were performed using a WATERGATE water suppression.

**Peptide 15**: Formylglycine peptide **3** (3 mg, 0.005 mmol, 1 equiv) and amine **F1** (2.6 mg, 0.011 mmol, 2.5 equiv) were stirred in dry MeOH (1 mL) and AcOH (20 μL) with molecular sieve (4 Å, 1 spatula) for 1 h at room temperature. NaCNBH_3_ (2.5 equiv) was added and the mixtures was stirred for 18 h. Molecular sieves were filtered off and the mixture was concentrated under reduced pressure. The residue was purified by RP flash column chromatography yielding **15** as a white solid (1.46 mg, 41 %); **HRMS**: (ESI): C_35_H_50_N_8_O_15_ [M], 822.3396 Da. calcd *m/z* 823.3474 [M+H]^+^, 821.3317 [M−H]^−^, found *m/z* 823.3477 [M+H]^+^, 821.3316 [M−H]^−^.

**Peptide 16**: Following the procedure for peptide **15**, **3** (3 mg, 0.005 mmol, 1 equiv) and **F4** (1.8 mg, 0.011 mmol, 2.5 equiv) yielded **16** as white solid (2.1 mg, 57 %); **HRMS**: (ESI): C_35_H_50_N_8_O_16_ [M], 838.3345 Da. calcd *m/z* 839.3423 [M+H]^+^, 837.3267 [M−H]^−^, found *m/z* [M+H]^+^, [M−H]^−^.

**Peptide 17**: Following the procedure for peptide **15**, **3** (3 mg, 0.005 mmol, 1 equiv) and barbituric acid (2.6 mg, 0.011 mmol, 2.5 equiv) yielded **17** as white solid (1.89 mg, 48 %); **HRMS**: (ESI): C_31_H_45_N_9_O_16_ [M], 799.2984 Da. calcd *m/z* 800.3063 [M+H]^+^, 798.2906 [M−H]^−^, found *m/z* 800.3058 [M+H]^+^, 798.2912 [M−H]^−^.

**Peptide 18**: Following the procedure for peptide **3**, using the unnatural amino acid **19**, **18** was obtained as a white solid (26.6 mg, 51 %) from 200 mg resin. ^**1**^ **H NMR**: (500 MHz, DMSO‐d6): δ=8.21 (1H, d, *J*=7.4 Hz, Leu6 NH), 8.16 (1H, d, *J*=7.7 Hz, Ala NH), 8.06 (1H, d, *J*=6.8 Hz, H‐2 Asp1 NH), 8.03 (1H, d, *J*=7.5 Hz, H‐15 fGly5 NH), 7.97 (1H, d, *J*=8.2 Hz, Asp3 NH), 7.78 (1H, d, *J*=7.7 Hz, Glu4 NH), 7.23 (1H, m, NH_2_), 7.04 (1H, m, NH_2_), 6.98 (2H, d, *J*=8.8 Hz, Ar−H), 6.64 (2H, d, *J*=8.8 Hz, Ar−H), 4.59–4.54 (1H, m, Leu6 Hα), 4.54–4.50 (1H, m, Ala2 Hα), 4.44 (1H, q, *J*=7.1 Hz, Dap5 Hα), 4.30–4.24 (1H, m, Glu4 Hα), 4.26–4.24 (1H, m, 1.9 Hz, Asp3 Hα), 4.21 (1H, d, *J*=2.7 Hz, Asp1 Hα), 4.18 (1H, d, *J*=7.2 Hz, Dap5 NH), 3.34 (1H, dd, *J*=13.0, 7.1 Hz, Dap5 CH_2_), 3.23 (1H, dd, *J*=13.0, 7.3 Hz, Dap5 CH_2_), 2.77–2.69 (2H, m, Asp1 Hβ), 2.69–2.66 (1H, m, Asp3 Hβ), 2.59–2.55 (1H, m, Asp3 Hβ), 2.26–2.19 (2H, m, Glu4 Hβ), 1.97–1.89 (1H, m, Glu4 Hγ), 1.85 (3H, s, Ac CH_3_), 1.81–1.73 (1H, m, Glu4 Hγ), 1.63–1.56 (1H, m, Leu6 Hγ), 1.51–1.45 (2H, m, Leu6 Hβ), 1.22 (3H, d, *J*=7.1 Hz, Ala2 CH_3_), 0.87 (3H, d, *J*=6.6 Hz, Leu6 Hδ), 0.82 (3H, d, *J*=6.5 Hz, Leu6 Hδ). **HRMS**: (ESI): C_34_H_48_F_3_N_9_O_15_S [M], 911.2943 Da. calcd *m/z* 912.3021 [M+H]^+^, 934.2840 [M+Na]^+^, found *m/z* 912.3021 [M+H]^+^, 934.2838 [M+Na]^+^.

**2‐N‐(9H‐Fluoren‐9‐ylmethoxycarbonylamino)‐3‐(4‐trifluoromethyl‐sulfonamido)‐phenyl)‐amino‐propanoic acid 19**: To a solution of **20** (390 mg, 1.193 mmol, 1 equiv) and NaHCO_3_ (251 mg, 2.983 mmol, 2.5 equiv) in water (10 mL) was added N‐(9‐fluorenylmethoxy‐carbonyloxy)‐succinimide (403 mg, 1.193 mmol, 1 equiv) dissolved in 1,4‐dioxane (10 mL) dropwise at 0 °C. The mixture was allowed to slowly warm up to room temperature and was subsequently stirred for 18 h. Water was added (25 mL) and the mixture extracted with ethyl acetate (3×50 mL). The organic layer was washed with 0.2 M HCl (40 mL) and brine (40 mL) and dried over Na_2_SO_3_. The solvents were removed under reduced pressure and the crude product purified via MPLC yielding **19** as a brown solid (575.8 mg, 88 %). ^**1**^ **H NMR**: (700 MHz, DMSO‐d6) δ=7.90 (2H, d, J=7.5 Hz, Fmoc Ar−H), 7.72 (2H, d, J=7.5 Hz, Fmoc Ar−H), 7.68 (1H, d, J=8.3 Hz, N*H*SO_2_CF_3_), 7.42 (2H, t, J=7.5 Hz, Fmoc Ar−H), 7.33 (2H, t, J=7.5 Hz, Fmoc Ar−H), 7.31 (1H, d, J=7.2 Hz, CON*H*), 6.99 (2H, d, J=8.8 Hz, Ar−H), 6.63 (2H, d, J=8.8 Hz, Ar−H), 4.35–4.29 (2H, m, Fmoc CH_2_), 4.24 (1H, d, J=7.1 H, Hα), 4.23–4.20 (1H, m, Fmoc C*H*), 3.42 (1H, dd, J=13.6, 7.6 Hz, Hβ), 3.36 (1H, dd, J=13.5, 7.5 Hz, Hβ). ^**13**^ **C NMR**: (176 MHz, DMSO) δ=172.88 (*C*OOH), 156.62 (*C*ONH), 147.93 (Ar−C_quart_), 144.25 (Fmoc Ar−C_quart_), 141.18 (Fmoc Ar−C_quart_), 128.09 (Fmoc Ar−C), 127.54 (Fmoc Ar−C), 127.17 (Fmoc Ar−C), 125.68 (Fmoc Ar−C), 122.78 (Ar−C_quart_), 121.26, 120.57 (Ar−C), 119.42 (CF_3_), 112.83 (Ar−C), 66.17 (Fmoc *C*H), 53.75 (Cα), 47.08 (Fmoc *C*H_2_), 44.52 (Cβ). **HRMS**: (ESI): C_25_H_22_F_3_N_3_O_6_S [M], 549.1181 Da. calcd *m/z* 550.1260 [M+H]^+^, 572.1079 [M+Na]^+^, found *m/z* 550.1260 [M+H]^+^, 572.1080 [M+Na]^+^.

**2‐Amino‐3‐(4‐trifluoromethyl‐sulfonamido‐phenyl)‐amino‐propanoic acid 20**: A mixture of L‐2‐*N*‐Boc‐2,3‐diamino‐propanoic acid (Boc‐Dap‐OH) (500 mg, 2.45 mmol, 1 equiv), 1‐fluoro‐4‐nitrobenzene (415 mg, 2.94 mmol, 1.2 equiv) and K_2_CO_3_ (407 mg, 2.94 mmol, 1.2 equiv) in ethanol (15 mL) was stirred for 24 h at 90 °C. To the crude residue of this reaction was added 10 % Pd/C and the flask wash flushed with H_2_ and left under H_2_ atmosphere for 18 h at room temperature. The catalyst was filtered off, the solvent evaporated under vacuum and purified by column chromatography to provide *N*‐2‐Boc‐3‐(4‐aminophenyl)‐2,3‐diamino‐propanoic acid as brownish solid (593 mg, 82 %). ^**1**^ **H NMR**: (500 MHz, DMSO‐*d*_6_) δ=6.41 (2H, d, *J*=8.5 Hz, Ar−H), 6.33 (2H, d, *J*=8.5 Hz, Ar−H), 6.08 (1H, d, *J*=6.0 Hz, CON*H*) 3.64 (1H, q, *J*=6.0 Hz, Hα), 3.11 (1H, dd, *J*=11.1, 5.9 Hz, Hβ), 2.94 (1H, dd, *J*=11.1, 6.1 Hz, Hβ), 1.38 (9H, s, CH_3_). ^**13**^ **C NMR**: (126 MHz, DMSO‐*D*_6_) δ=172.95 (*C*OOH), 155.55 (Boc *C*O), 141.09 (Ar−C_quart_), 139.52 (Ar−C_quart_), 116.07 (Ar−C), 114.29 (Ar−C), 78.08 (Boc C_quart_), 54.97 (Cα), 48.36 (Cβ), 28.79 (Boc CH_3_). **HRMS**: (ESI): C_14_H_21_N_3_O_4_ [M], 295.1532 Da. calcd *m/z* 296.1610 [M+H]^+^, 294.1454 [M−H]^−^, found *m/z* 296.1612 [M+H]^+^, 294.1451 [M−H]^−^. The obtained intermediate (400 mg, 1.36 mmol, 1 equiv) was dissolved in anhydrous DCM (10 mL) under an argon atmosphere and the reaction mixture was cooled to 0 °C. Trifluoromethanesulfonic anhydride (275 μL, 1.63 mmol, 1.2 equiv) was added dropwise to the reaction mixture and stirred for 30 min at 0 °C, after which the mixture was slowly warmed to room temperature and stirred further for 18 h. The reaction was diluted with water (20 mL), filtered and the feed was washed twice with MeCN/H_2_O (2×10 mL, 50 : 50 v/v). The solvents were evaporated under vacuum and purified by column chromatography to provide **20** as brownish solid (338 mg, 76 %). ^**1**^ **H NMR**: (500 MHz, DMSO‐d_6_) δ=7.20 (1H, d, J=9.0 Hz, H‐9), 6.86 (1H, d, J=9.0 Hz, H‐4), 6.77 (2H, d, J=8.8 Hz, H‐7), 6.45 (2H, d, J=8.8 Hz, H‐6), 3.53 (1H, dd, J=8.0, 4.2 Hz, H‐1), 3.42 (1H, dd, J=13.3, 4.2 Hz, H‐3), 3.22 (1H, dd, J=13.3, 8.0 Hz, H‐3). ^**13**^ **C NMR**: (126 MHz, DMSO−D6) δ=174.37 (C‐2), 143.13 (C‐5), 125.16 (C‐6), 123.58 (C‐8), 118.43 (C‐10), 113.56 (C‐7), 53.77 (C‐1), 45.69 (C‐3). **HRMS**: (ESI): C_10_H_12_F_3_N_3_O_4_S [M], 327.0501 Da. calcd m/z 328.0579 [M+H]^+^, 326.0422 [M−H]^−^, found m/z 328.0582 [M+H]^+^, 326.0419 [M−H]^−^.

**(*Z*)‐2‐(4‐(2‐(1,3‐Bis‐(4‐nitrophenyl)‐5‐oxo‐1,5‐dihydro‐4H‐pyrazol‐4‐ylidene)‐hydrazinyl)‐phenyl)‐acetic acid (21)**: A solution of NaNO_2_ (0.17 mmol) in H_2_O (100 μL) was added at 0 °C to a suspension of 2‐(4‐aminophenyl)‐acetic acid (0.17 mmol) in 4 M HCl (0.3 mL). The acidic solution was stirred for 1 h at 0 °C, after which a solution of 2,5‐bis(4‐nitrophenyl)‐2,4‐dihydro‐3H‐pyrazol‐3‐one (0.15 mmol) in THF (2 mL) was added and the mixtures was stirred for 10 min at 0 °C. Afterwards a solution of 1.3 M NH_4_OH (0.2 mL) was added, stirred for 1 h at 0 °C and for 24 h at r.t. The suspension was filtered under suction, the solid was washed with H_2_O (2×20 mL), and dried in air. A mixture of EtOAc:DCM:n‐Hex (3 mL, 1 : 1 : 8 *v*/*v*) was added in a sealed flask for 2 h. The suspension was filtered off, washed with Et2O and dried under vacuo to provide product **21**. ^**1**^ **H NMR**: (500 MHz, DMSO‐d6): δ=8.55 (2H, d, J=9.3 Hz, Ar−H), 8.51 (2H, d, J=9.1 Hz, Ar−H), 8.34 (2H, d, J=9.1 Hz, Ar−H), 8.29 (2H, d, J=9.3 Hz, Ar−H), 7.54 (2H, d, J=7.9 Hz, Ar−H), 7.29 (2H, d, J=7.9 Hz, Ar−H), 3.56 (2H, s, CH_2_) ppm. ^**13**^ **C NMR**: (126 MHz, DMSO‐d_6_): δ=173.6 (*C*OOH) 157.9 (C‐7), 147.1 (Pyr C_quart_), 146.3 (Ar−C_quart_), 143.88 (Pyr C_quart_), 142.4 (Ar−C_quart_), 141.3 (Ar−C_quart_), 130.4 (Ar−C), 130.0 (Ar−C_quart_), 129.1(Ar−C), 125.4 (Ar−C), 123.9 (Ar−C), 122.3 (Ar−C_quart_), 121.2 (Ar−C_quart_), 120.9 (Ar−C), 117.6 (Ar−C), 32.0 (*C*H_2_) ppm. **HRMS**: (ESI): C_23_H_16_N_6_O_7_ [M], 488.1080 Da. calcd *m/z* 489.1159 [M+H]^+^, 487.1002 [M−H]^−^, found *m/z* 489.1162 [M+H]^+^, 487.1005 [M−H]^−^. **Anal**: calcd for C_22_H_14_F_3_N_7_O_7_S: C, 56.56; H, 3.30; N, 17.21; found: C, 57.12; H, 3.58; N, 17.56.

**(Z)‐4‐(2‐(1,3‐Bis‐(4‐nitrophenyl)‐5‐oxo‐1,5‐dihydro‐4H‐pyrazol‐4‐ylidene)‐hydrazinyl)‐phenyl)‐1,1,1‐trifluoromethanesulfonamide (22)**: Following the procedure for compound 21, using N‐(4‐aminophenyl)‐1,1,1‐trifluoromethanesulfonamide (82 mg, 0.34 mmol, 1.1 equiv) and 2,5‐bis(4‐nitrophenyl)‐2,4‐dihydro‐3H‐pyrazol‐3‐one (100 mg, 0.31 mmol, 1 equiv), 22 was obtained as an orange solid (111 mg, 62 %). HRMS: (ESI): C_22_H_14_F_3_N_7_O_7_S [M], 577.0628 Da. calcd *m/z* 578.0706 [M+H]^+^, found *m/z* 578.0696 [M+H]^+^. Anal: calcd for C_22_H_14_F_3_N_7_O_7_S: C, 45.76; H, 2.44; N, 16.98; found: C, 45.78; H, 2.54; N, 16.98.

**(*Z*)‐2‐(4‐(2‐(1,3‐Bis‐(4‐nitrophenyl)‐5‐oxo‐1,5‐dihydro‐4H‐pyrazol‐4‐ylidene)‐hydrazinyl)‐phenoxy)‐acetic acid (23)**: Following the procedure for compound **21**, using 2‐(4‐aminophenoxy)‐acetic acid (28 mg, 0.17 mmol, 1.1 equiv) and 2,5‐bis(4‐nitrophenyl)‐2,4‐dihydro‐3H‐pyrazol‐3‐one (50 mg, 0.15 mmol, 1 equiv), **23** was obtained as an orange solid (35 mg, 46 %). **HRMS**: (ESI): C_23_H_16_N_6_O_8_ [M], 504.1030 Da. calcd *m/z* 505.1108 [M+H]^+^, 503.0951 [M−H]^−^, found *m/z* 505.1106 [M+H]^+^, 503.0976 [M−H]^−^. **Anal**: calcd for C_23_H_16_N_6_O_8_: C, 54.77; H, 3.20; N, 16.66; found: C, 55.23; H, 3.18; N, 15.62.

**Protein Expression and purification**: DNA encoding the catalytic domain of human PTP1B (amino acids 1–321) was subcloned into the pQLinkH vector. The gene encoding the N‐terminal His_7_‐tagged protein was over‐expressed at 17 °C in E. coli Rosetta (DE3). The purification procedure comprises an affinity chromatography on a 5 mL HisTrap FF crude column (GE Healthcare), charged with Ni^2+^, and a size‐exclusion chromatography on a Superdex 26/60 column (GE Healthcare) equilibrated with 25 mM HEPES‐NaOH (pH=7.5), 50 mM NaCl, 1 mM DTT. The His7‐tag was cleaved with tobacco etch virus protease at 20 °C prior to the gel‐filtration step. The catalytic domain of human SHP2 (amino acids 225–541) was purified from *E. coli* B21 (DE3). The overexpressed protein was fused to an N‐terminal His_10_‐tag. Cells were harvested and resuspended in 25 mM Tris‐HCl (pH=7.5), 50 mM NaCl, 1 % (*v/v*) Triton X‐100, 10 % (*v/v*) glycerol, 1 mM DTT. After cell lysis, debris was removed by centrifugation at 12,000 rpm for 30 min. The supernatant was filtered prior to affinity purification with Ni‐NTA agarose (Qiagen). The slurry was washed with 50 mM Tris‐HCl (pH=8.0), 500 mM NaCl supplemented with 25 mM imidazole. Reasonably pure protein was eluted with 200–300 mM imidazole. The purification was completed by size‐exclusion chromatography with a Superdex 75 16/600 column (GE Healthcare) equilibrated with 20 mM HEPES‐NaOH (pH=7.5), 50 mM NaCl, 1 mM DTT.

**Enzyme activity assays of SHP2 and PTP1B**: The catalytic activity of SHP2 catalytic domain and PTP1B were monitored using the fluorogenic substrate DiFMUP (6,8‐difluoro‐4‐methylumbelliferyl phosphate). Phosphatase reactions were performed at room temperature in 384‐well black plate, clear flat bottom, low flange, non‐binding surface (Corning, Cat# 3766) using a final volume of 20 μL and the following assay buffer conditions: 50 mM MOPS, pH=6.5, 200 mM NaCl, 50 μM TCEP, 0.03 % Tween‐20 (freshly added prior to each measurement). Test compounds were dissolved in DMSO or buffer at stock concentrations of 50, 20 or 10 mM and serially diluted. Enzyme (5 nM PTP1B or 2.5 nM SHP2 catalytic domain) and different concentrations of the tested compounds were incubated in buffer for 30–60 min at room temperature. Measurements were performed with a final concentration of 2.5 % DMSO unless stated otherwise at 37 °C and were performed in triplicate. Enzymatic reactions were started by adding DiFMUP (Invitrogen, cat# D6567) concentrations matching the experimentally determined *K_M_* values of the enzymes of 67 μM (PTP1B) or 72 μM (SHP2). Samples were excited at a wavelength of 360 nm and emitted fluorescence was recorded at 460 for 10 min using a microplate reader (infinite M1000 Pro, Tecan). Initial slope of fluorescence was determined in triplicate and *IC_50_* values were calculated with GraphPad Prism 5. Determined *IC_50_* values were converted into the corresponding *K_I_* values applying the Cheng Prusoff equation *K_I_* =*IC_50_*/(1+[S]/*K_M_* ).

**Dynamic ligation screening of nucleophilic fragments with 3**: Under assay conditions described above, PTP1B or SHP2 were pre‐incubated with **3** (100 μM) for 5 min. Subsequently, (200 μM) of the respective fragments were added and the resulting mixtures were incubated for 30 min at RT, prior to DiFMUP addition.

**Determination of apparent** ***K***_***I***_ **‐values of 11 in a time‐dependent assay**: Under the conditions described above, PTP1B was pre‐incubated with peptide **3** in different concentrations (from 5 mM to 10 μM final assay concentration) for 5 mins. Subsequently, over the course of 60 mins (every 15 min), 2 equivalents of fragment **F1** were added to each of the different concentrations of **3** at RT, prior to DiFMUP addition.

**Determination of apparent** ***K***_***I***_ **‐values of 12 in a concentration‐dependent assay**: Under the conditions described above, PTP1B was pre‐incubated with peptide **3** at different concentrations (from 2.5 mM to 0.3 μM final assay concentration) for 5 min. Subsequently, fragment **F2** was added at three different concentrations (1 equiv, 2 equiv, and 4 equiv) and the resulting mixtures were incubated for 30 min at RT, prior to DiFMUP addition.

**Computational Methods**: The protein X‐ray diffraction crystal structure of mutated PTP1B (PDB code: 1PTU)^[24]^ was prepared for docking with Schrödinger's Protein Preparation Wizard.^[32]^ The residue Ser215 was mutated back to the natural Cys215. The protonation states of amino acid sidechains were assigned with PROPKA at pH 7.0. Small molecules and crystal water were deleted. The hydrogen‐bond network was optimized and a brief molecular mechanics minimization using the OPLS4^[33]^ force field was run. The structures of **3**, **9**, **11**, **12**, **15**, **16**, and **18** were docked to the binding pocket of PTP1B using Schrödinger's GLIDE.^[23]^ A receptor grid was generated using the default settings with OH‐ and SH‐ groups within the binding pocket allowed to rotate. Ligand docking was performed with the XP protocol. Non‐planar amide conformations were penalized, and halogens were included as weak noncovalent interaction acceptors of hydrogen bond type.

**Cellular experiments**: HeLa cells were cultivated in DMEM buffer with 10 % FCS in 75 cm^2^ cell culture flasks at 37 °C, 5 % CO_2_. At confluency of about 75 %, cells were seeded in a density of 250,000 cells/mL in 6‐well plates and incubated for 24 h. Subsequently, medium was removed and replaced by DMEM buffer with 0.1 % BSA (1 mL per well) and incubated for another 16 h. Hepatocyte growth factor (HGF, 20 ng/well) and test compounds in different final concentrations (1 μL, in DMSO) were added. Control wells received the same amount of DMSO (final concentration 0.1 %) or no addition. Plates were incubated for 1 h, then washed with cold PBS and shaken with lysis buffer (mPer reagent ThermoScientific # 78501 with 1 mM NaF, 2 mM Na_3_VO_4_, 25× protease inhibitor) for 5 min. Cell lysates were transferred to Eppendorf cups and centrifuged at 4 °C with 10,000 g for 10 min. Total protein was quantified in cell lysates using RotiNanoquant (Carl Roth # K880.1) and 30 μg of protein was applied to 12 %‐SDS‐PAGE with a run time of 90 min at 150 V. Then protein was blotted in Towbin bufer to an PVDF‐membrane over 1 h at 100 V, and the membrane was saturated with 20 ml TBS‐Tween/ 2 % BSA, 1 h at RT. The blot was incubated with anti‐phospo‐ERK as primary antibody (Cell Signaling # 9106, dilution 1 : 2000 in TBS‐Tween/ 2 % BSA) overnight at 4 °C, washed 3×5 min with TBS‐Tween, and incubated with anti‐mouse IgG‐hrp as secondary antibody (Santa Cruz # sc2031, 1 : 6000 in TBS‐ Tween/ 2 % BSA, RT, 1.5 h). The blot was again washed 3×5 min with TBS‐Tween and imaged with the ECL system (ThermoScientific # 34080) at Syngene PXi Imager. From the same lysis sample, under the same conditions, ERK 1/2 and β‐tubulin were blotted. For β‐tubulin, the antibody incubation time was adjusted to 2 h at RT.

## Conflict of interest

The authors declare no conflict of interest.

1

## Supporting information

As a service to our authors and readers, this journal provides supporting information supplied by the authors. Such materials are peer reviewed and may be re‐organized for online delivery, but are not copy‐edited or typeset. Technical support issues arising from supporting information (other than missing files) should be addressed to the authors.

Supporting Information

## Data Availability

The data that support the findings of this study are available in the supplementary material of this article.
