# Supplementary material for: A Formylglycine‐Peptide for the Site‐Directed Identification of Phosphotyrosine‐Mimetic Fragments
Source: Chemistry. 2022 Aug 23;28(57):e202201282. doi: 10.1002/chem.202201282 (PMC9804470; doi:10.1002/chem.202201282)
Supplement: Supplementary file 1 — Supporting Information [file CHEM-28-0-s001.pdf]

Chemistry–A European Journal

Supporting Information

A Formylglycine-Peptide for the Site-Directed Identification of Phosphotyrosine-Mimetic Fragments

Markus Tiemann, Eric Nawrotzky, Peter Schmieder, Leon Wehrhan, Silke Bergemann, Vera Martos, Wei Song, Christoph Arkona, Bettina G. Keller, and Jörg Rademann*

Supporting Information

A Formylglycine-Peptide Used for the Site-Directed Identification of Phosphotyrosine-Mimetic Fragments

Markus Tiemann, Eric Nawrotzky, Peter Schmieder, Leon Wehrhan, Silke Bergemann, Vera Martos, Wei Song, Christoph Arkona, Bettina G. Keller, and Jörg Rademann*

Table of Contents

General methods ... 3

NMR Experiments with Formylglycine Peptides 3, 9, and 10 ... 4

NMR Ligation Experiments with Peptide 3 ... 8

Reaction of Formylglycin-Peptide 3 with DTT ... 19

Fragments Tested in Fragment Ligation Experiments (Selection) ... 22

Computational Methods and Docking Experiments ... 32

Supporting Chemical Methods and NMR Spectra ... 37

References ... 43

General methods

All moisture sensitive reactions were performed in glassware that was previously vacuum heat dried and flushed with Ar or N_2 using Schlenk technology.

All other chemicals were purchased from Sigma (Merck), ABCR and Fluka and were used without any further purification.

Dry DMF and ACN were bought as anhydrous and stored over activated molecular sieves 4Å. All other dry solvents were obtained from a column-based solvent system (MBraun, MB-SPS-800).

Removal of volatile components was performed using rotary evaporators from Heidolph with a hot water bath of 40 °C, if not otherwise stated. The high vacuum obtained with an oil pump corresponds to 1 µbar or less. Lyophilized fractions were obtained from Christ Alpha 2-4 LD.

Product isolation was conducted on Biotage, Isolera™ Spektra equipped with KP-Sil or RP-C18 SNAP Cartridges with appropriate HPLC grade solvent mixtures and deionized water, or with HPLC (Agilent Technologies, 1260 series, column Macherey-Nagel, Nucleodur 5 µm C18, 150 x 32 mm, equipped with Agilent 1260 Infinite diode array and multiple wavelength detector and fraction collector).

Thin layer chromatography analyses were conducted on Merck Aluminum sheets pre-coated with silica gel (Merck, 60 F_{254}). Detection was carried out using 254 nm UV-Light, followed by dipping in ceric ammonium molybdate or ninhydrin stains.

NMR spectra were measured on the following spectrometers: JEOL ECX400 (9.39 T), JEOL ECP500 (11.74 T), JEOL ECZ600 (14.09 T), Bruker Avance III 700 (16.44 T). Chemical shifts (δ) are reported in ppm and coupling constants (J) are given in Hz. ^{1}H and ^{13}C chemical shifts were referenced to the solvent peaks. ^{13}C and ^{31}P NMR spectra were hydrogen decoupled. Chemical shifts are given in ppm relative to the signal of the used deuterated solvent as internal standard.

NMR ligation experiments were performed in a 9:1 ratio of H_2O to D_2O in order to visualize acidic protons. To suppress the solvent signal in these experiments a Watergate-sequence was employed. Unless mentioned otherwise, compounds were added together and incubated for 30 min prior to the start of the measurement.

MS data were recorded with an analytical HPLC system (Agilent Technologies, 1100 Series) equipped with a Luna column, 3 µm C18 100 Å, 100 x 4.6 mm coupled with an ESI single quadrupole mass spectrometer LCMSD (Model# G1956B, Serial# US 44500857) from Agilent.

ESI high resolution mass spectra were recorded with an equipped with an analytical HPLC system (Agilent Technologies, Infinity II 1290), Zorbax Eclipse plus C-18 RRHD (2.1 x 50 mm, 1.8 µM, 95 Å) column, coupled with an ESI-Q-TOF iFunnel mass spectrometer (Agilent Technologies, 6550).

UV chromatograms were recorded with an HPLC system (Agilent Technologies, Infinity II 1290), Zorbax Eclipse plus C-18 RRHD (2.1 x 50 mm, 1.8 µM, 95 Å) column, coupled with a DAD detector (Agilent Technologies).

NMR Experiments with Formylglycine Peptides 3, 9, and 10

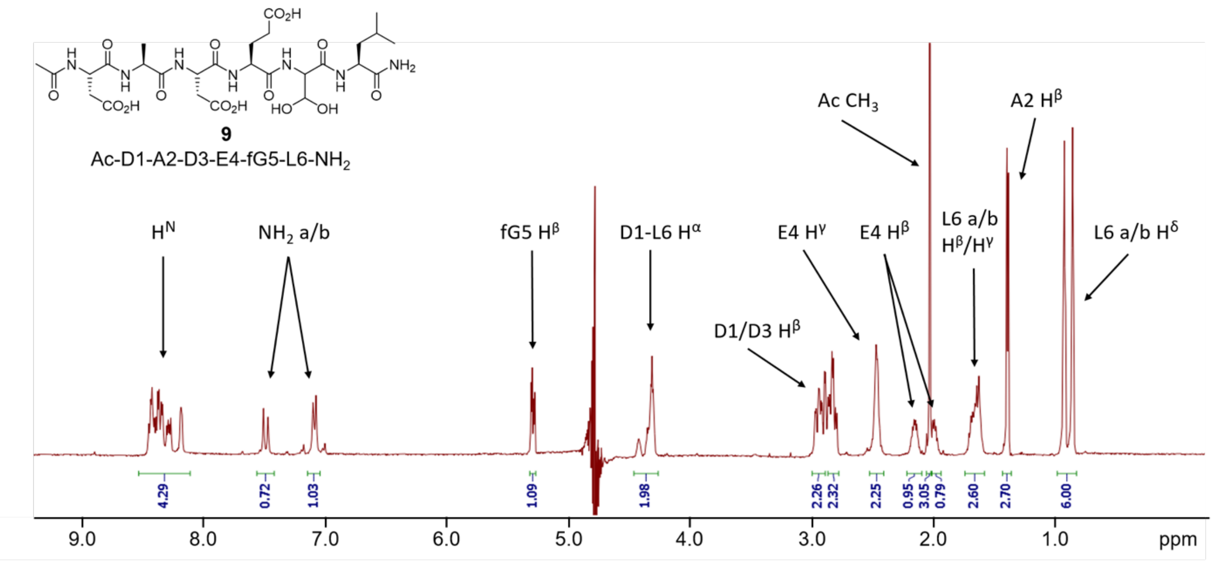

Supplementary Figure 1. ^{1}H, Watergate NMR spectrum of **9** (5 mM), in 9:1 H_2O/D_2O, 300 K.

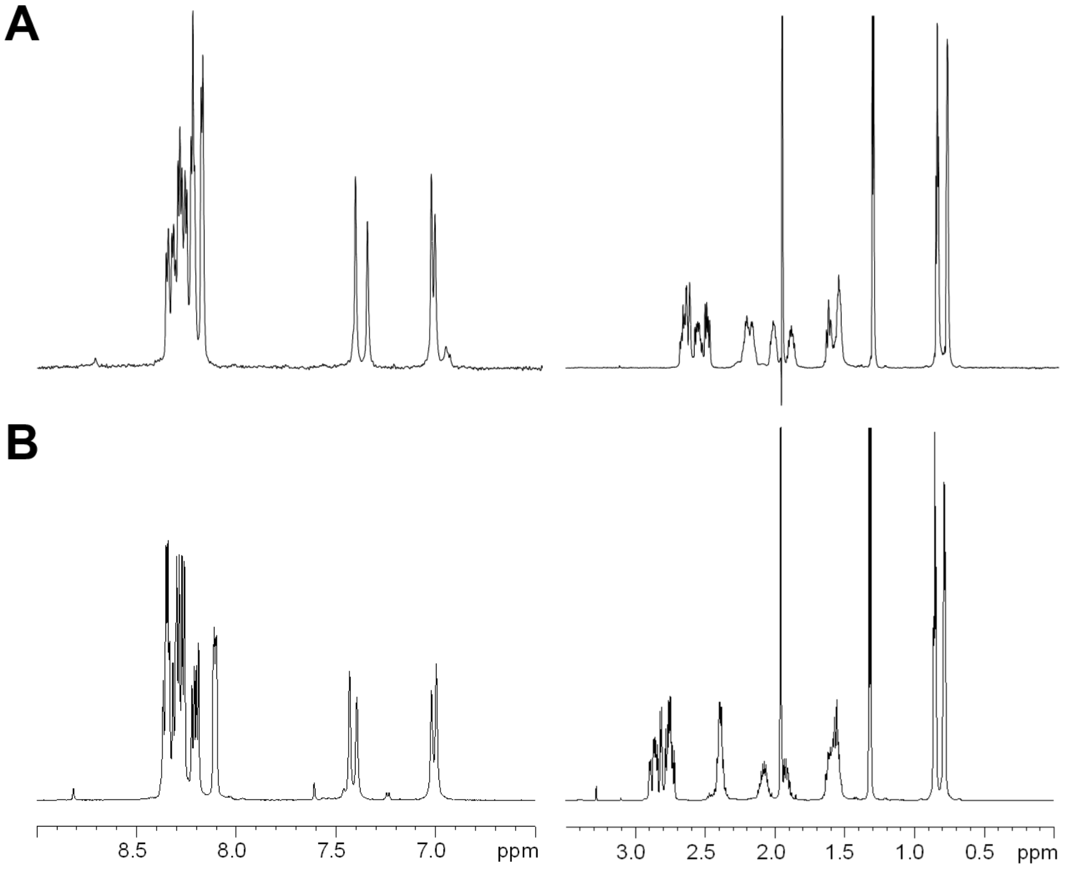

Supplementary Figure 2. ^{1}H, Watergate NMR spectrum of **9** (5 mM), A) in buffer (9:1 H_2O /D_2O, pH 6.5, 50 mM sodium phosphate buffer, 200 mM NaCl), B) in 9:1 H_2O/D_2O, 300 K.

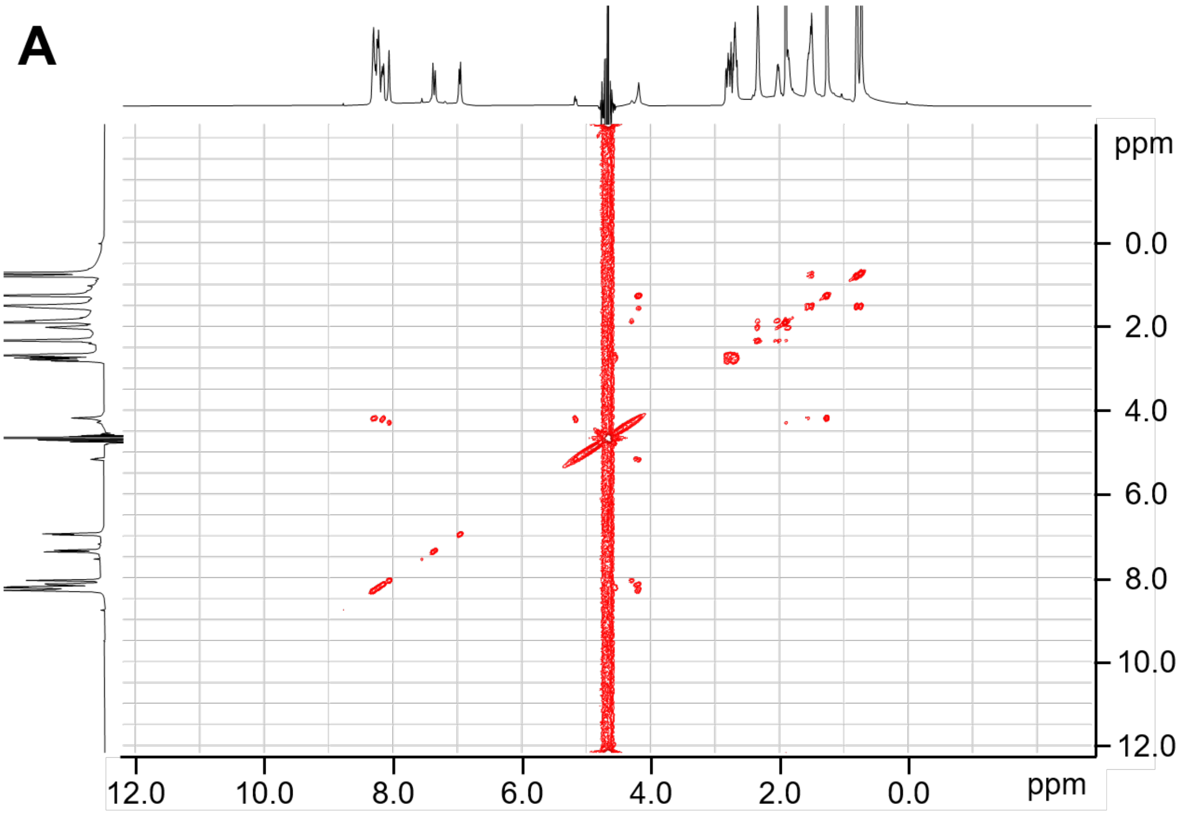

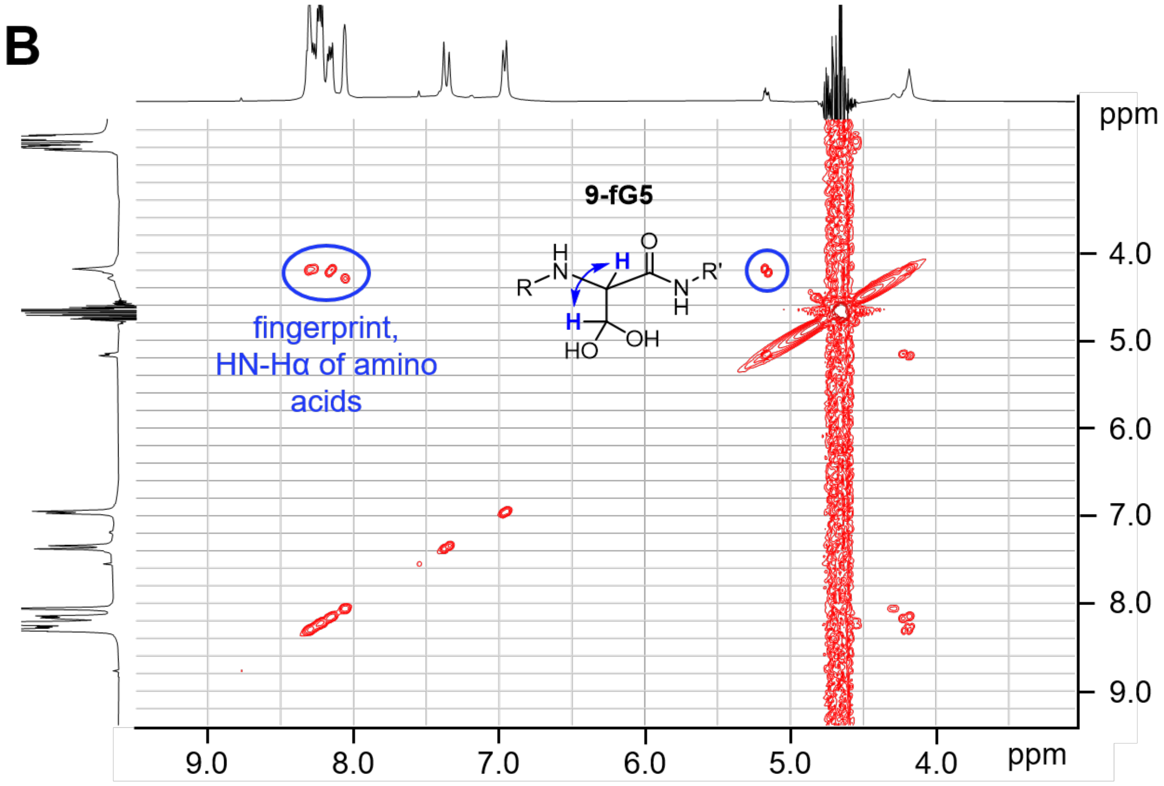

Supplementary Figure 3. ^{1}H-^{1}H COSY Watergate spectrum of **9** (5 mM) in 9:1 H_2O/D_2O, 300 K, A) full view, B) partial view with highlighted fingerprint region, showing HN-Hα crosscoupling of the amino acids A2, E4, fG5 and L6, as well as the Hα-Hβ cross peak of fG5.

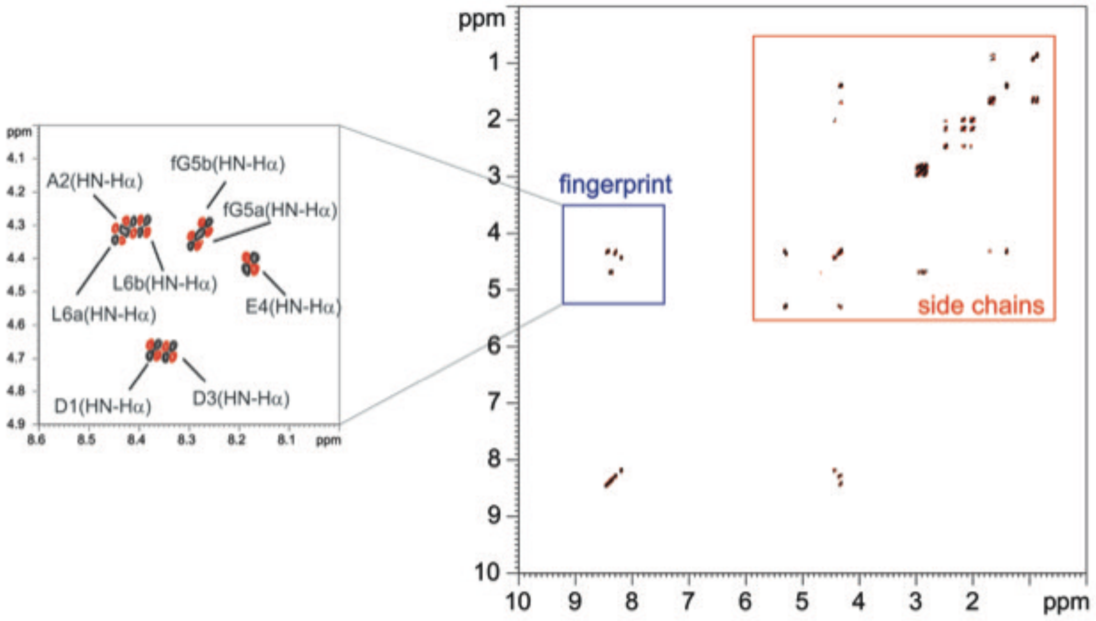

Supplementary Figure 4. DQF-^{1}H-^{1}H-COSY-COSY spectrum of **9** (5 mM) in 9:1 H_2O/D_2O, 300 K with highlighted side chain and fingerprint region, showing HN-Hα crosscoupling of the amino acids D1, A2, D3, E4, fG5 and L6.

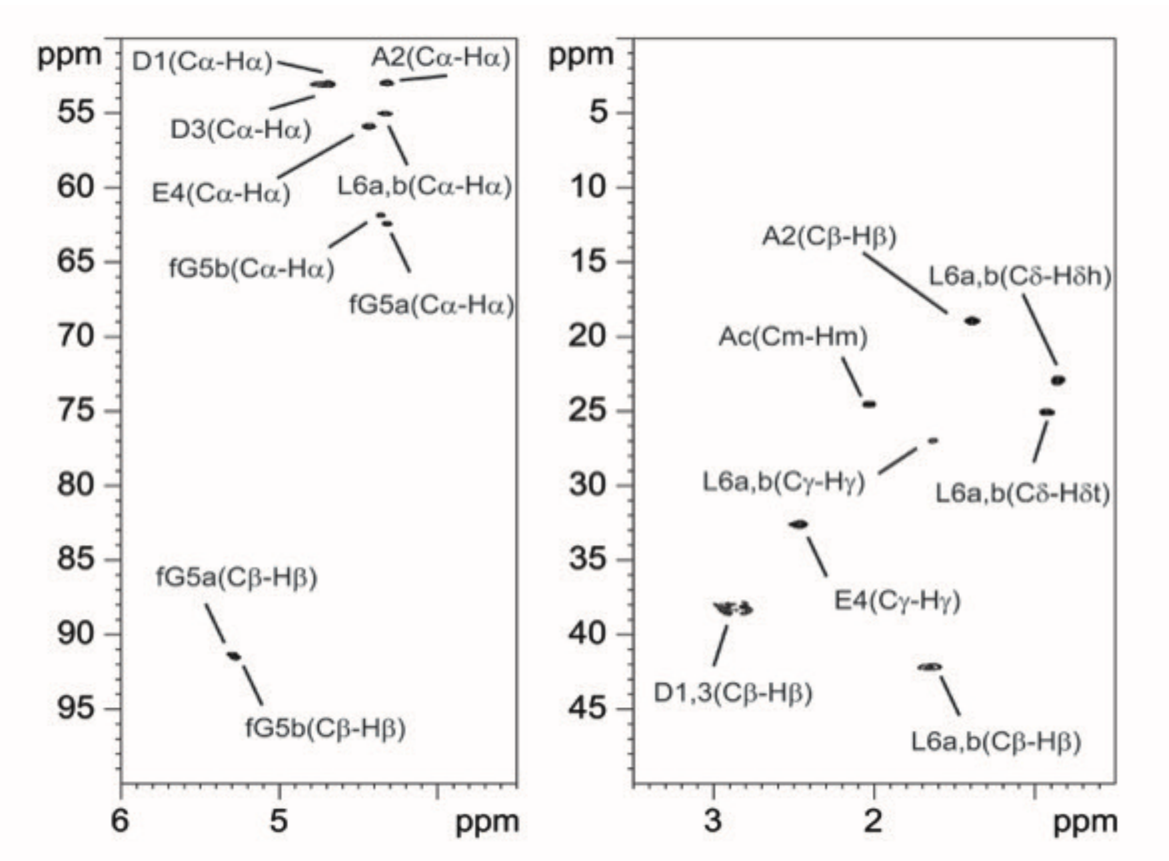

Supplementary Figure 5. Important regions of the HMQC spectrum of **9** (5 mM) in 9:1 H_2O/D_2O, 300 K, highlighting Cα-Hα as well as Cβ-Hβ crosscouplings of the amino acids.

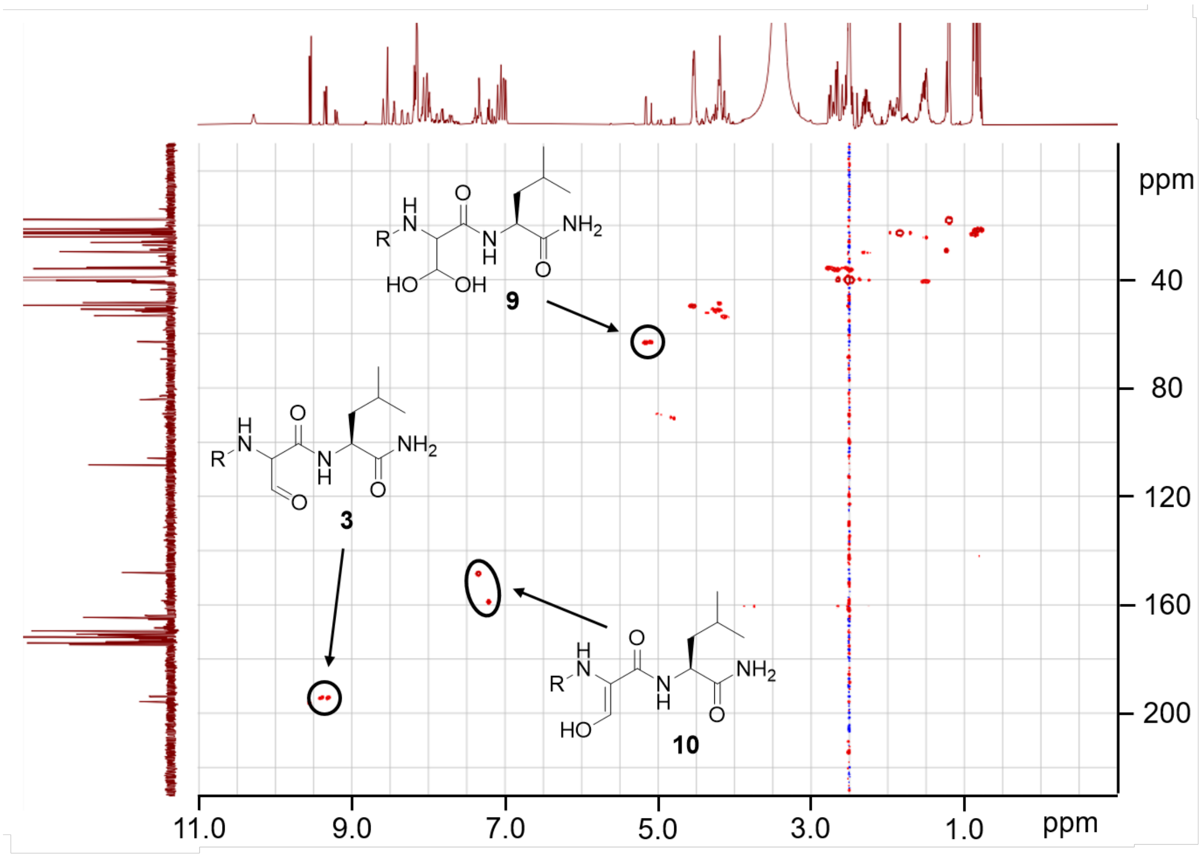

Supplementary Figure 6. HMQC of fG-peptide in DMSO, 300 K, highlighting the different Cβ-Hβ crosscouplings of the equilibrium of **3**, **9**, and **10**.

NMR Ligation Experiments with Peptide 3

For the ligation experiments of fG-peptide **9** with **F1** following procedure was used:

The samples for NMR-spectroscopy were prepared by dissolving **F1** and the fG-peptide **9** in buffer to yield samples of a concentration of 10 mM and 5 mM, respectively. The sample for monitoring the reaction was prepared by dissolving **F1** in a solution of **9** to yield a sample with 10 mM **F1** and 5 mM **9**, i.e. in a 2:1 ratio. The solutions were transferred to 5 mm sample tubes and in case of the mixture measured immediately.

All NMR spectra were recorded at 300 K at 600 MHz or 750 MHz (^{1}H frequency) on Bruker AV-III spectrometers (Bruker Cooperation, Billerica, MA, USA) using cryogenically cooled 5 mm QCI- or TCI probes with one-axis self-shielded gradients. The software used to control the spectrometer was topspin 3.5 pl6. Temperature had been calibrated using d_4-methanol and the formula of Findeisen et al.[1]

One-dimensional NMR-experiments were performed using a WATERGATE water suppression with excitation sculpting using 32 scans, an acquisition time of 330 msec and a spectral width of 12.5 kHz. To follow the reaction a series of such one-dimensional spectra using 128 scans each was recorded over night to yield a pseudo-2D data matrix. To assign the ligation product 11 the following NMR-experiments were recorded: A CLIP-COSY was recorded using 16 scans and 2048 x 512 complex data points and acquisition times of 163 and 51 ms in F2 and F1, respectively. A TOCSY was recorded with the same parameters, in addition, a ROESY using 64 scans. A ^{13}C-HMQC was recorded utilizing presaturation of the water resonance combined with echo/antiecho coherence selection using 64 scans and 512 × 256 complex data points, acquisition times of 41 and 8 ms in F2 and F1, respectively. In addition, a ^{15}N-SOFAST-HMQC was recorded using 4096 scans and 512 × 64 complex data points, acquisition times of 41 and 17 ms in F2 and F1, respectively.

Data were processed using topspin3.6 (Bruker Cooperation, Billerica, MA, USA), typically a squared sine bell shifted by 90° was used in both dimensions. Datasets were processed to yield a data matrix of 4096 by 2048 points. To analyze the time course of the reaction, one-dimensional datasets were extracted from the pseudo-2D data matrix using in-house scripts within topspin and subsequently analyzed using Kaleidagraph.

For the ligation experiments of fG-peptide **9** with **F1** following nomenclature was used:

9

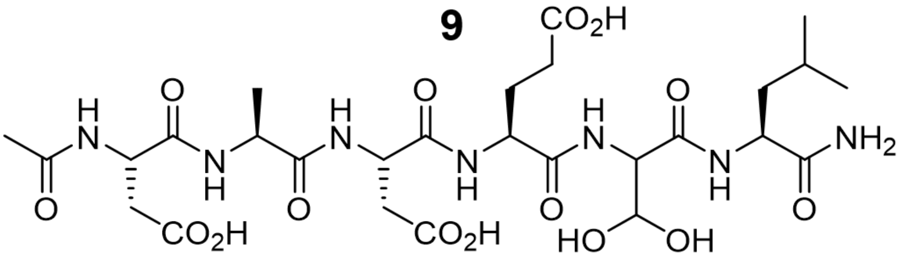

Ac-D1-A2-D3-E4-fG5-L6-NH_2

F1

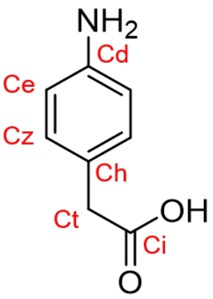

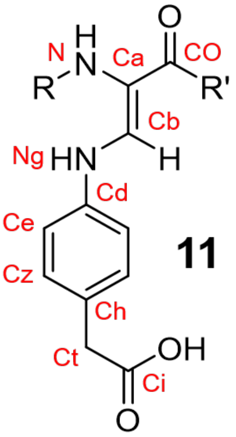

11

Ac-D1-A2-D3-E4-mG5-L6-NH_2

Supplementary Figure 7. Nomenclature for ligation NMR experiments.

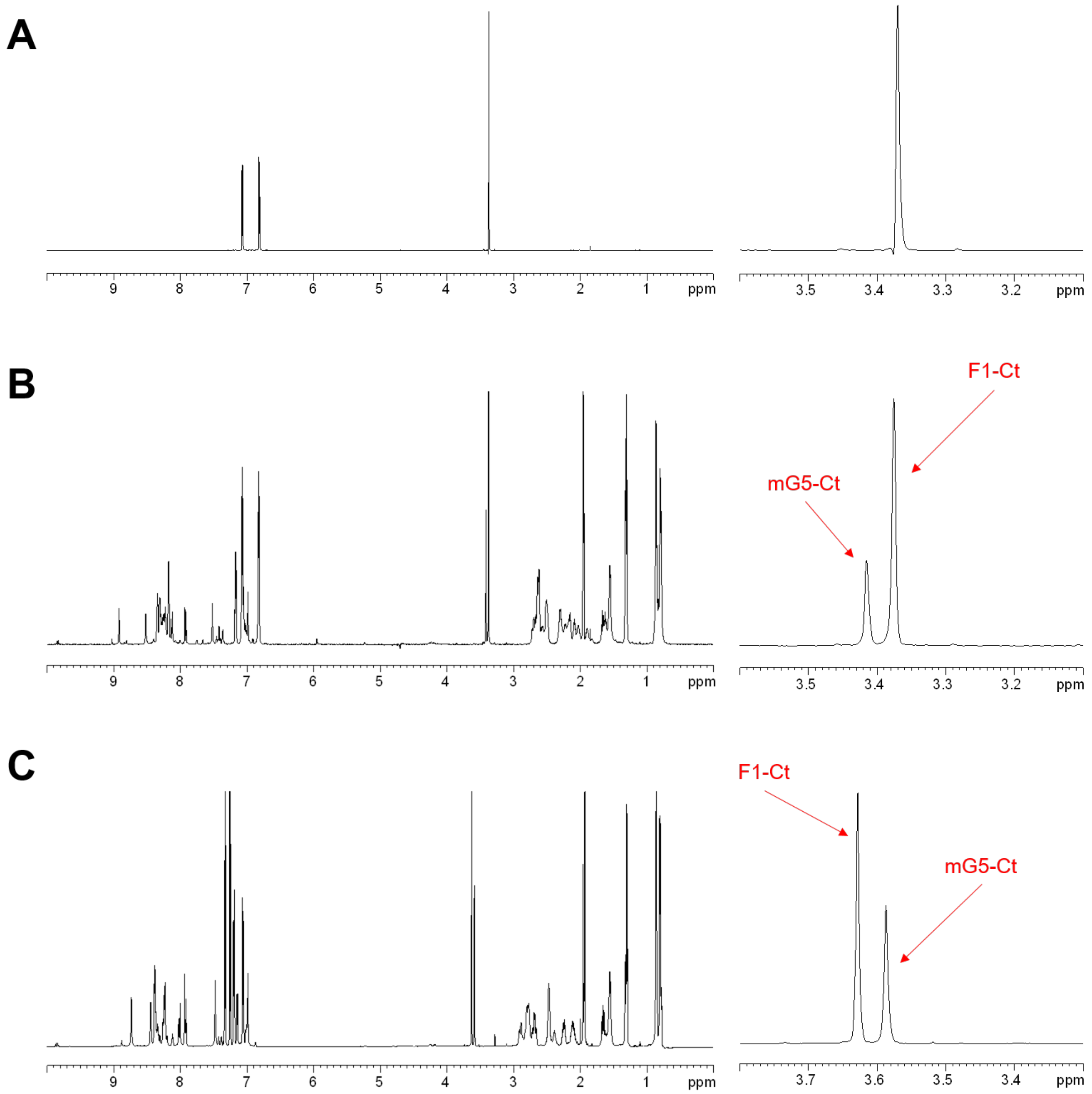

Supplementary Figure 8. ^{1}H, Watergate NMR spectra of A) **F1** (10 mM) in buffer (9:1 H_2O/D_2O, pH 6.5, 50 mM sodium phosphate, 200 mM NaCl), 300K, B) **9** (5 mM) with **F1** (10 mM) in buffer (9:1 H_2O/D_2O, pH 6.5, 50 mM sodium phosphate, 200 mM NaCl), 300K, C) **9** (5 mM) with **F1** (10 mM) in 9:1 H_2O/D_2O, 300 K.

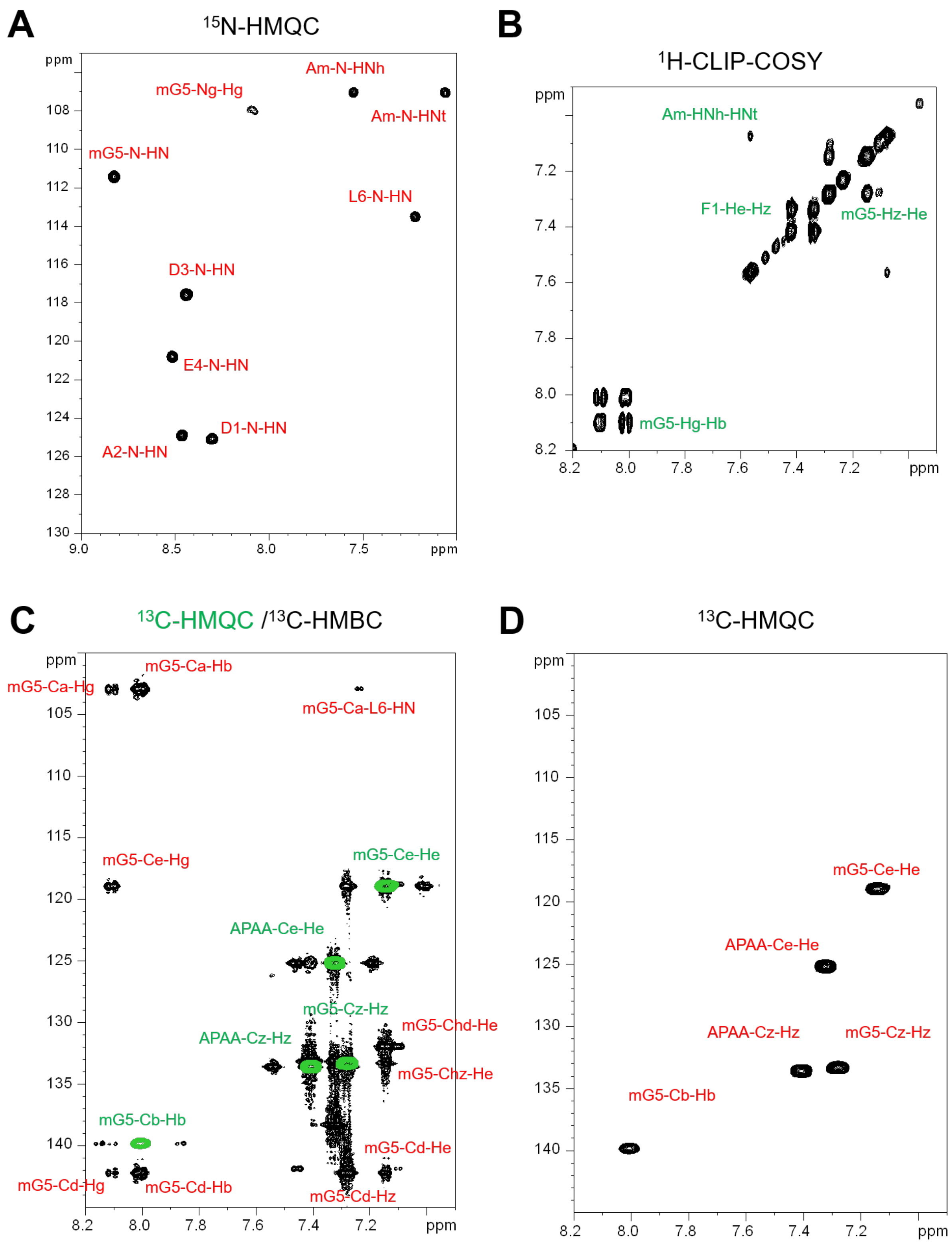

Supplementary Figure 9. Different NMR spectra of **9** (5 mM) with **F1** (10 mM) in 9:1 H_2O/D_2O, 300 K. A) ^{15}N-HMQC, B) ^{1}H-CLIP-COSY, C) Superimposed ^{13}C-HMQC (green) and ^{13}C-HMBC (black) spectra, D) ^{13}C-HMQC.

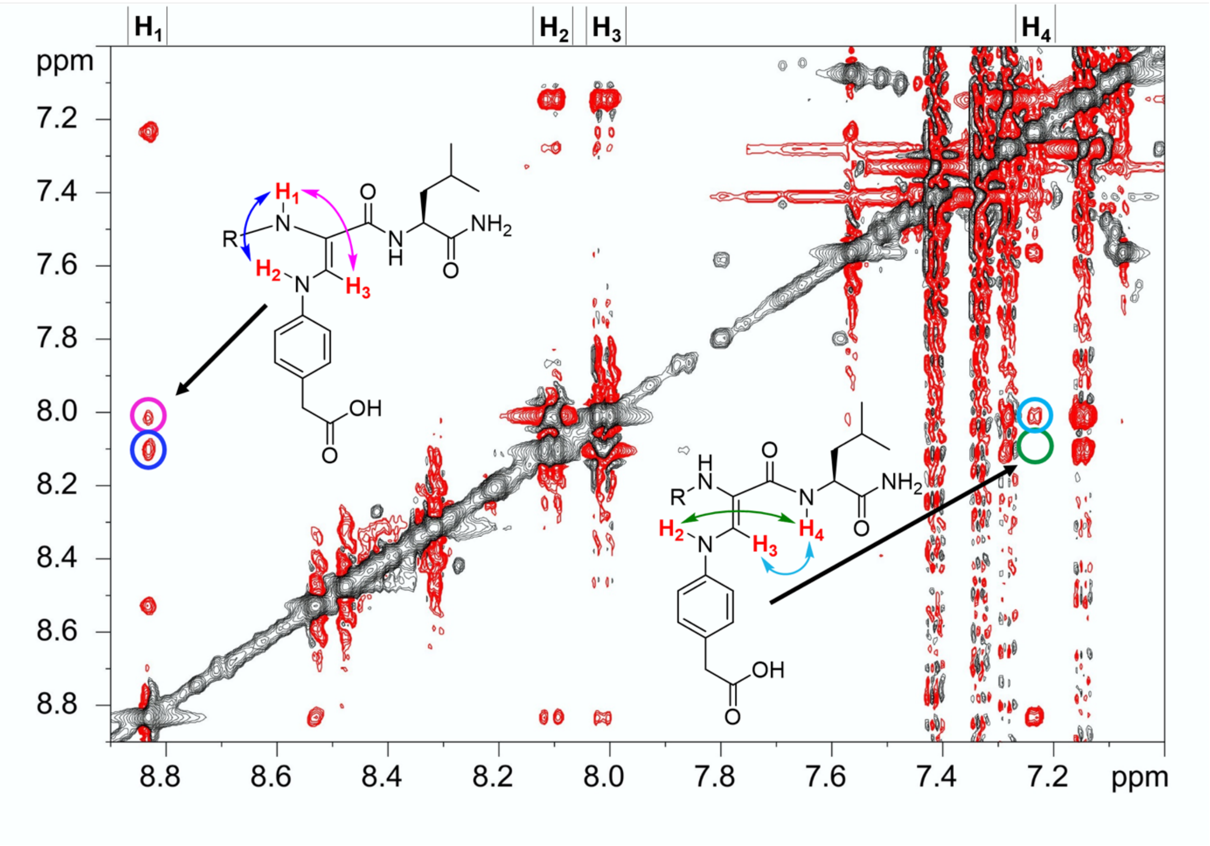

Supplementary Figure 10. ROESY spectrum of the ligation product **11** obtained in-situ from peptide **9** (5 mM) and fragment **F1** (10 mM) in 9:1 H_2O/D_2O, 300 K. Crosspeaks indicate the spatial proximity of H_1 with H_2 and H_3 and of H_4 with H_3 but not that of H_4 with H_2 confirming the formation of *E*-enamine **11**.

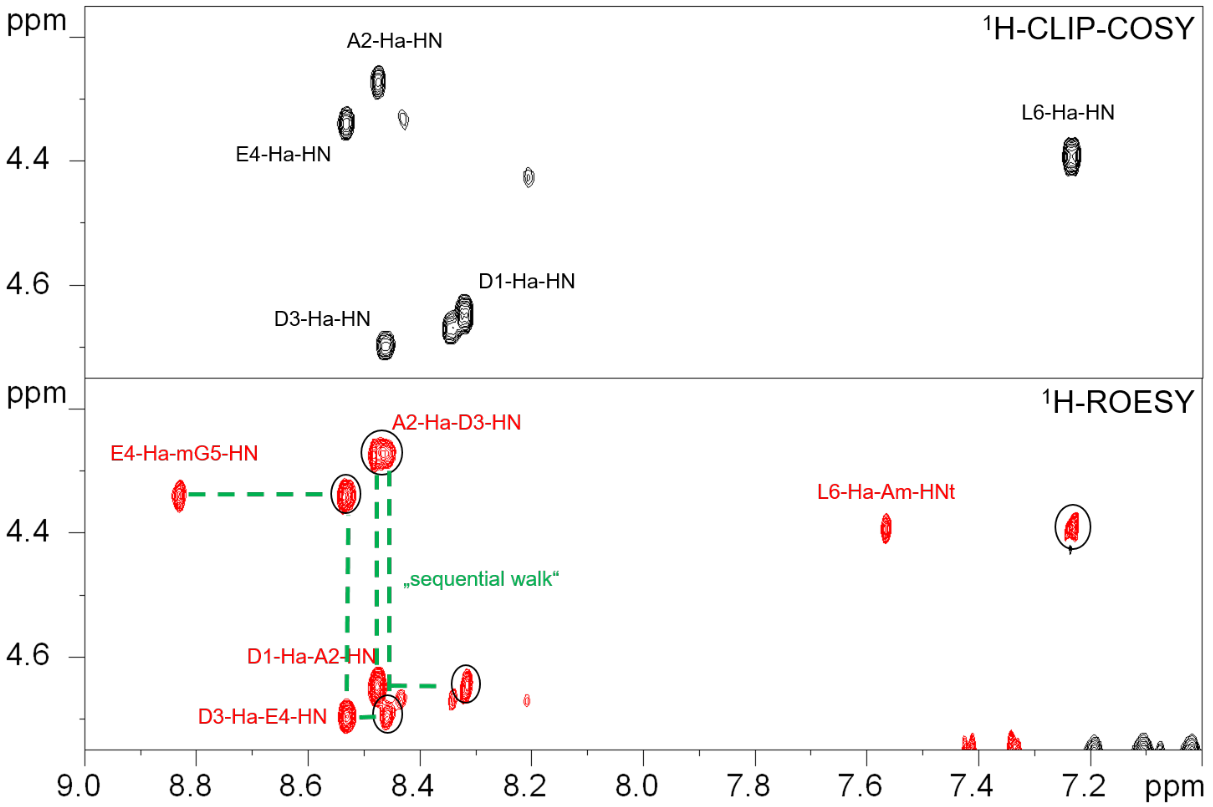

Supplementary Figure 11. ^{1}H-CLIP-COSY and ^{1}H-ROESY spectra of **9** (5 mM) with **F1** (10 mM) in 9:1 H_2O/D_2O, 300 K.

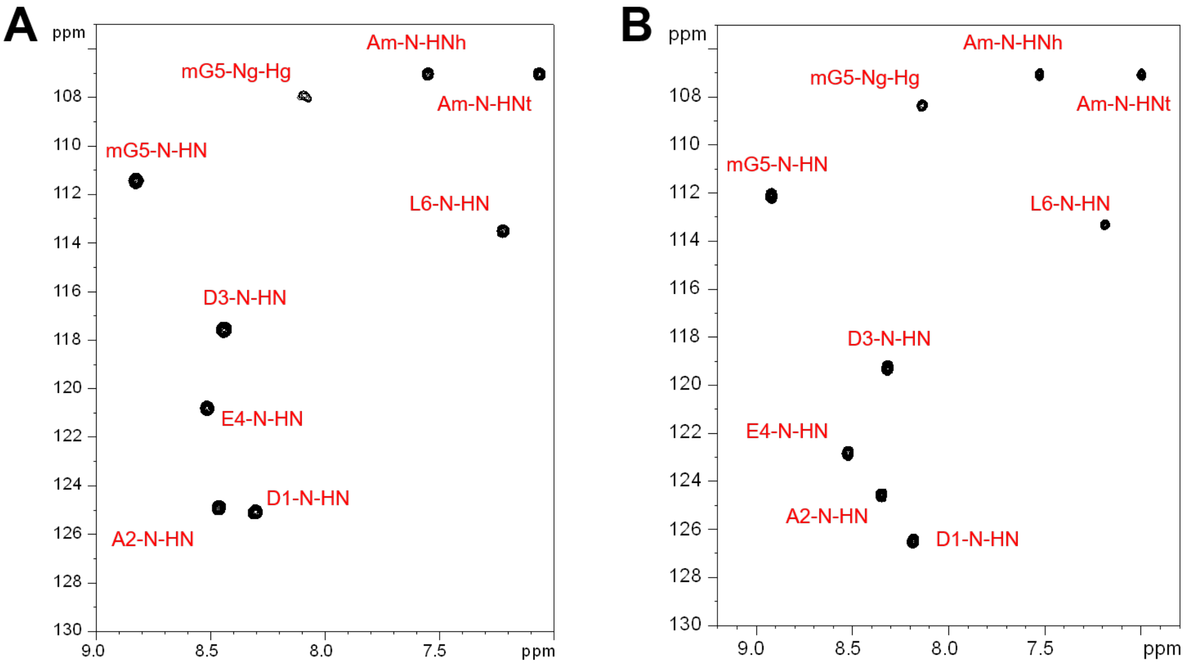

Supplementary Figure 12. ^{15}H-HMQC NMR spectra of A) **9** (5 mM) with **F1** (10 mM) in 9:1 H_2O/D_2O, 300 K, B) **9** (5 mM) with **F1** (10 mM) in buffer (pH 6.5, 50 mM sodium phosphate, 200 mM NaCl), 300 K.

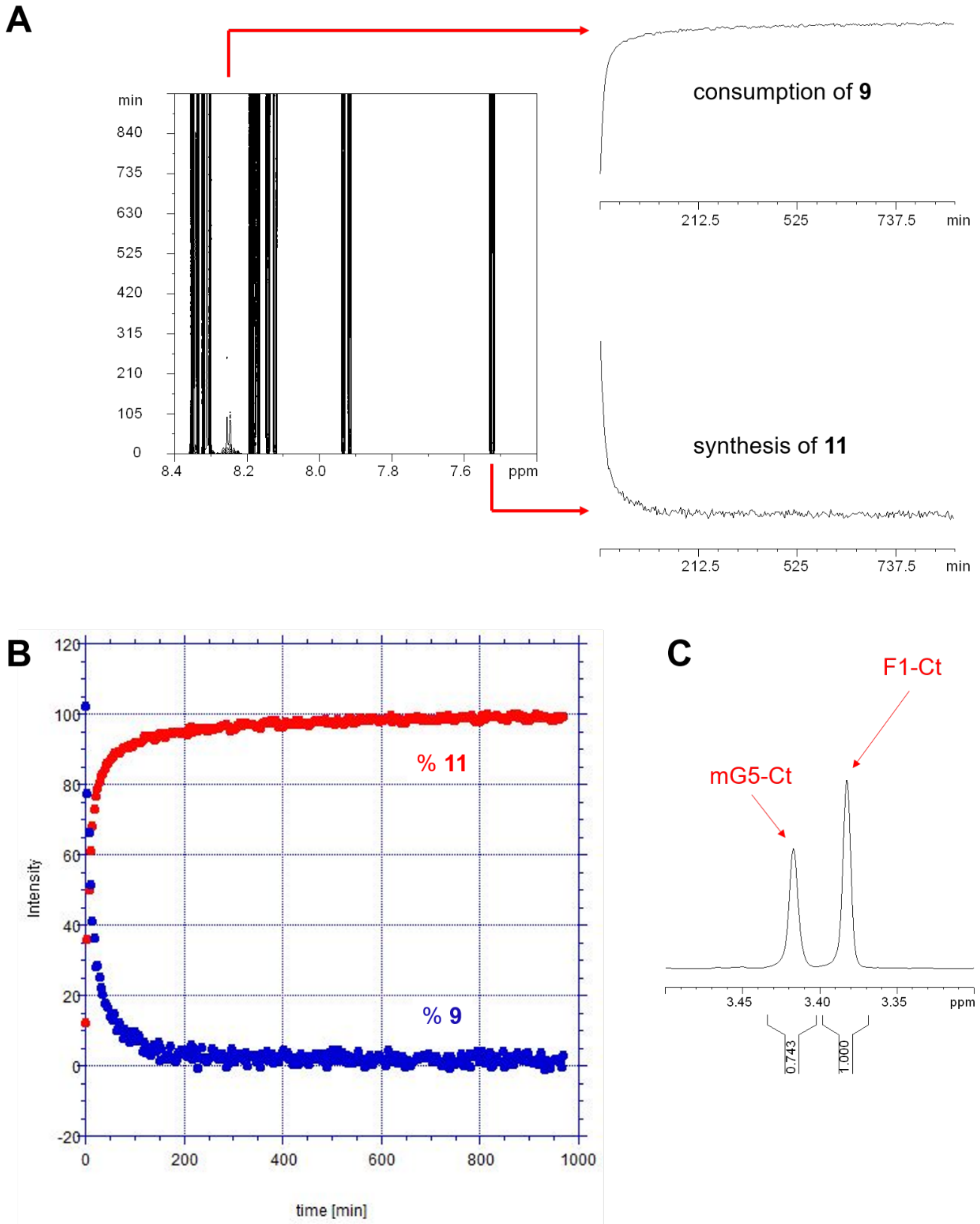

Supplementary Figure 13. A) Pseudo 2D Watergate NMR of **9** (5 mM) with **F1** (10 mM) in buffer (9:1 H_2O/D_2O, pH 6.5, 50 mM sodium phosphate, 200 mM NaCl), 300K. B) Visualization of A over 1000 min, C) Final ratio between mG5-Ct and F1-Ct after 1000 min in 9:1 H_2O/D_2O, 300 K.

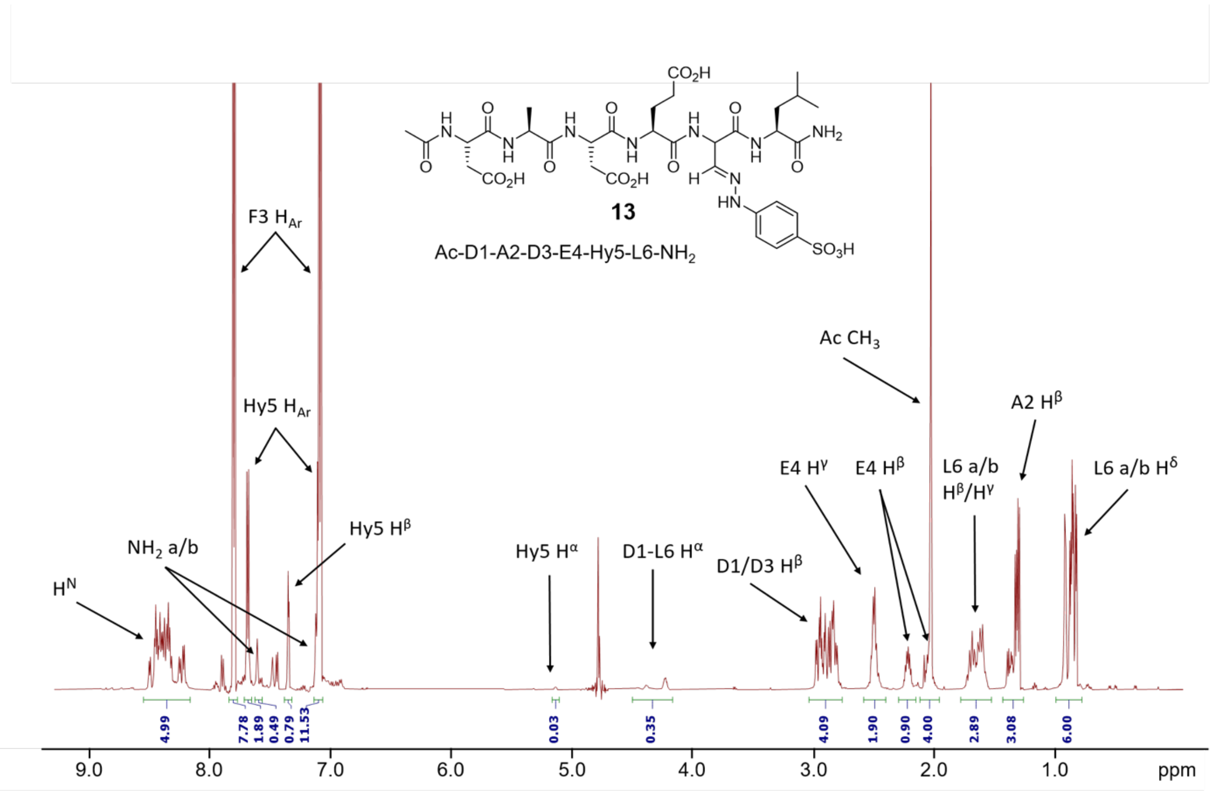

Supplementary Figure 14. ^{1}H, Watergate NMR spectrum of **9** (5 mM) with **F3** (25 mM) in 9:1 H_2O/D_2O, 300 K.

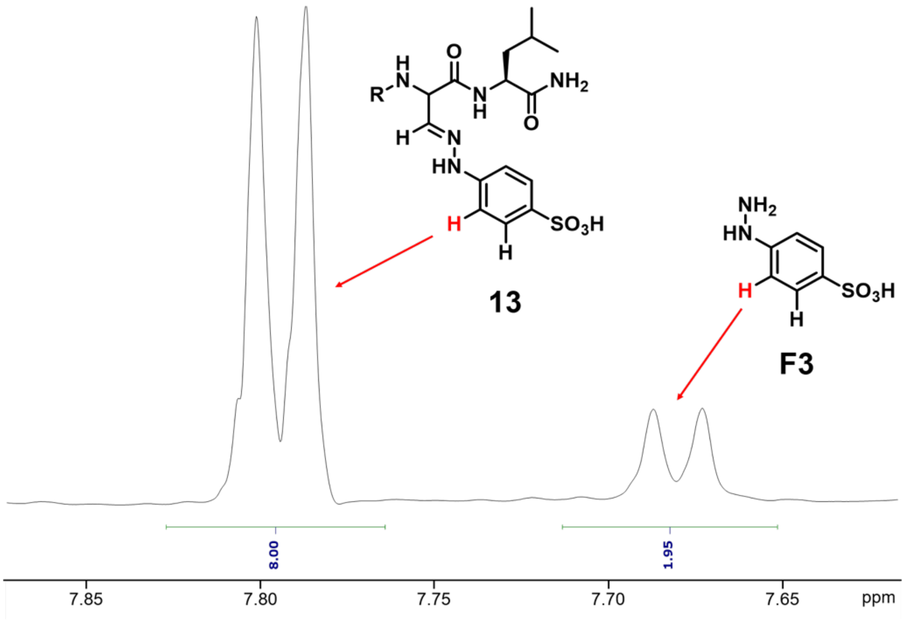

Supplementary Figure 15. ^{1}H, Watergate NMR spectrum of **9** (5 mM) with **F3** (25 mM) in 9:1 H_2O/D_2O, 300 K shows a 8:2 ratio between the aromatic protons of free **F3** and hydrazone product **13**, indicating full conversion.

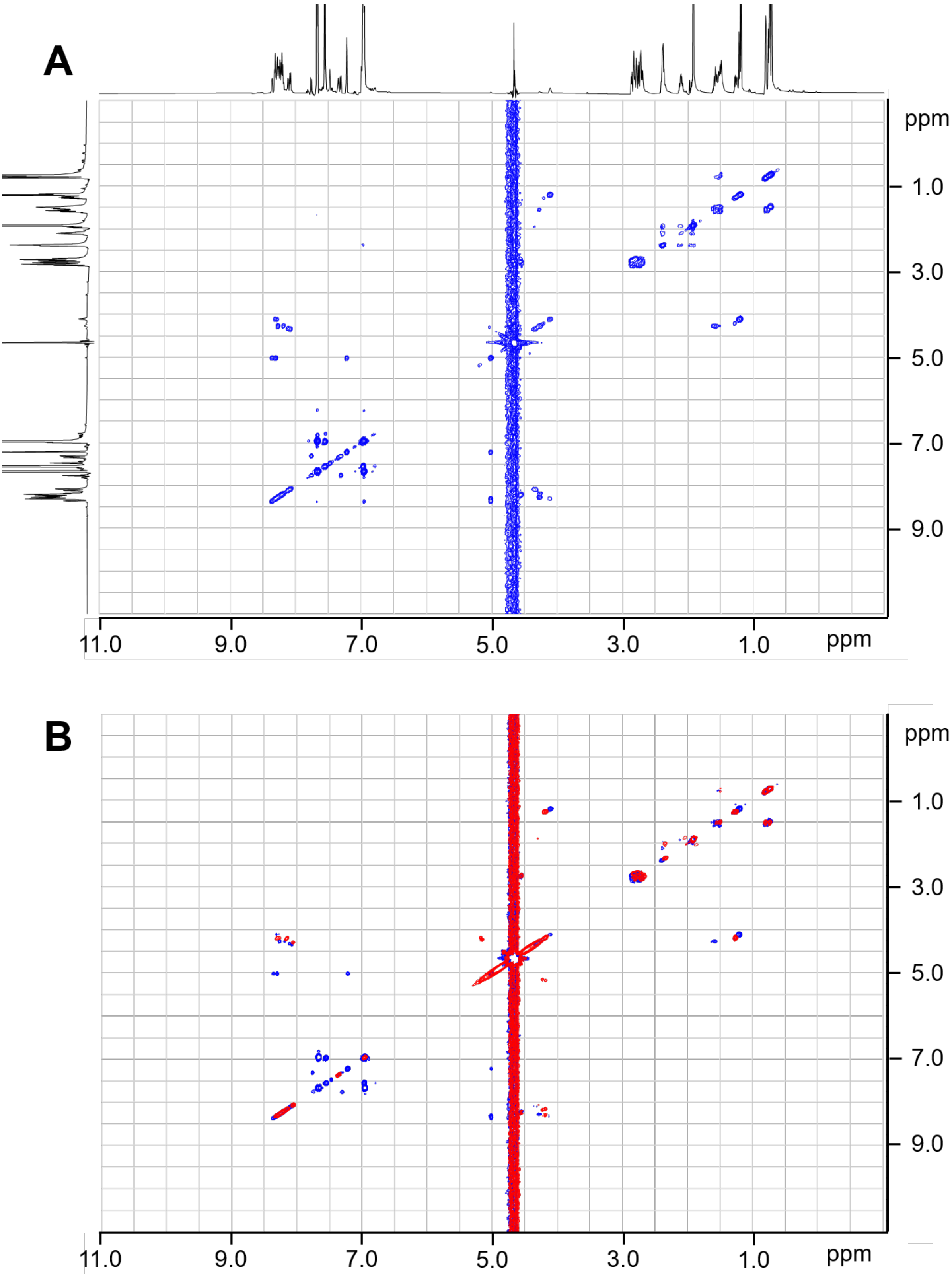

Supplementary Figure 16. A) ^{1}H-^{1}H-COSY spectrum of **9** (5 mM) with **F3** (25 mM) in 9:1 H_2O/D_2O, 300 K. B) Superimposed spectrum of A) (blue) with ^{1}H-^{1}H-COSY spectrum of **9** (red) (5 mM) in 9:1 H_2O/D_2O, 300 K (partial view with highlighted interactions in main paper).

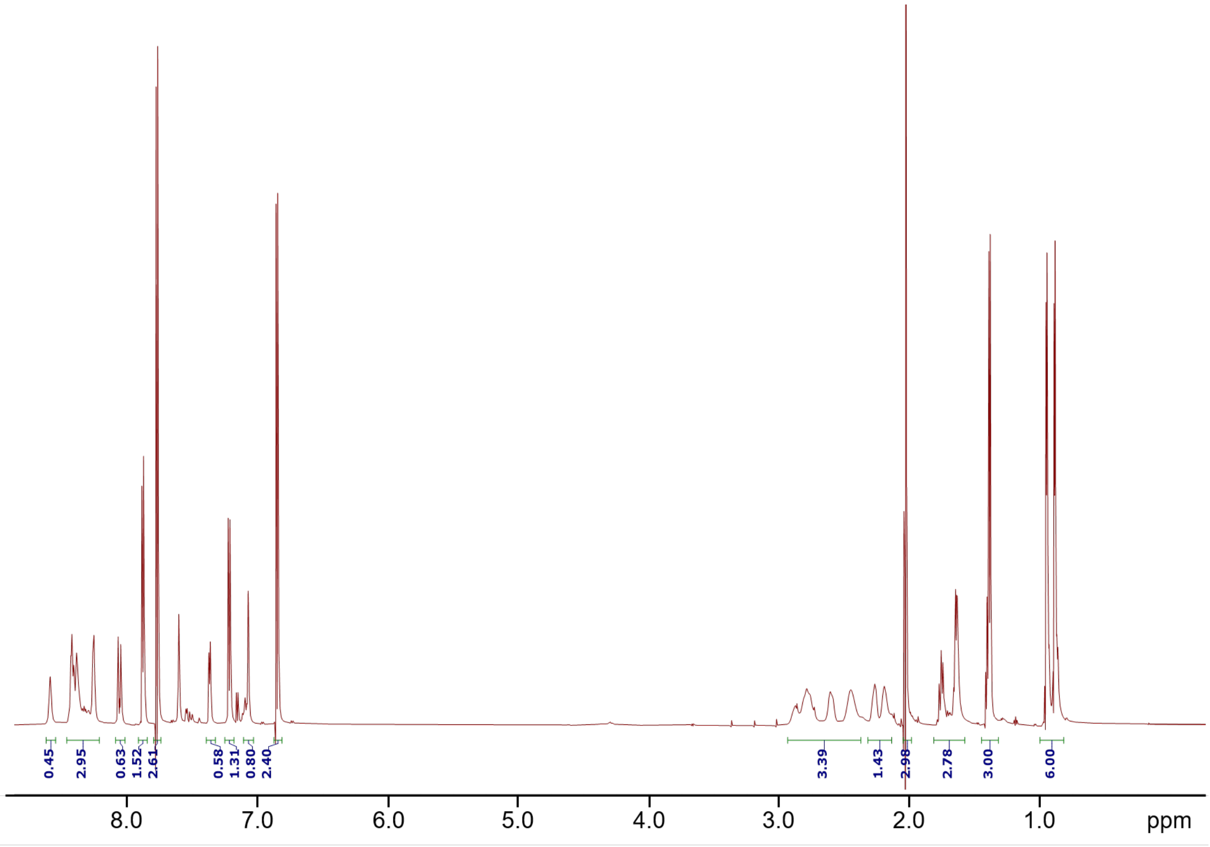

Supplementary Figure 17. ^1H, Watergate NMR spectra of **9** (5 mM) with 4-aminobenzoic acid (10 mM) in buffer (9:1 H_2O/D_2O, pH 6.5, 50 mM sodium phosphate, 200 mM NaCl), 300K

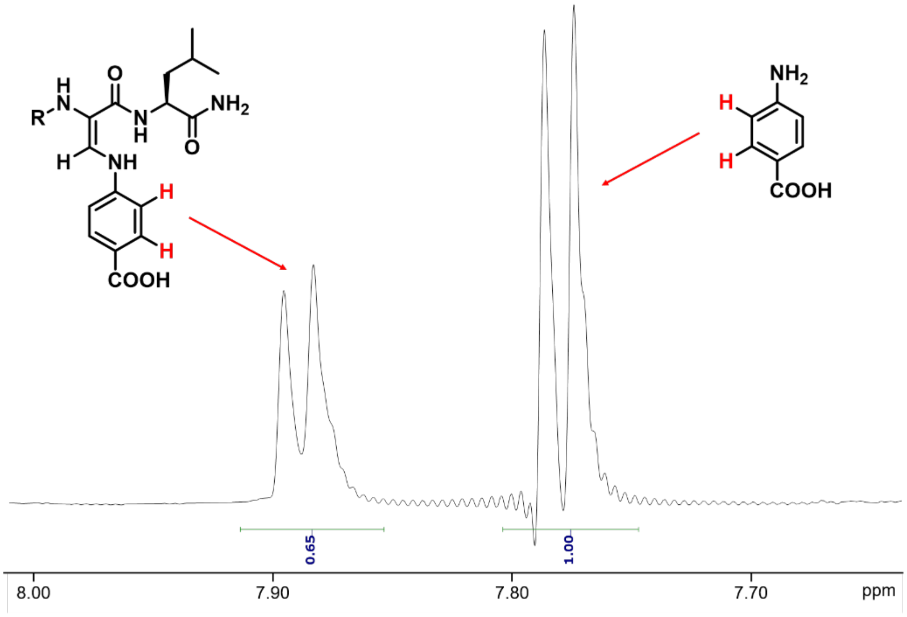

Supplementary Figure 18. ^1H-Watergate NMR spectrum of **9** (5 mM) with 4-aminobenzoic acid (10 mM) in 9:1 H_2O/D_2O, 300 K shows a 0.65:1 ratio between the aromatic protons of free 4-aminobenzoic acid and enamine product indicating 79% conversion.

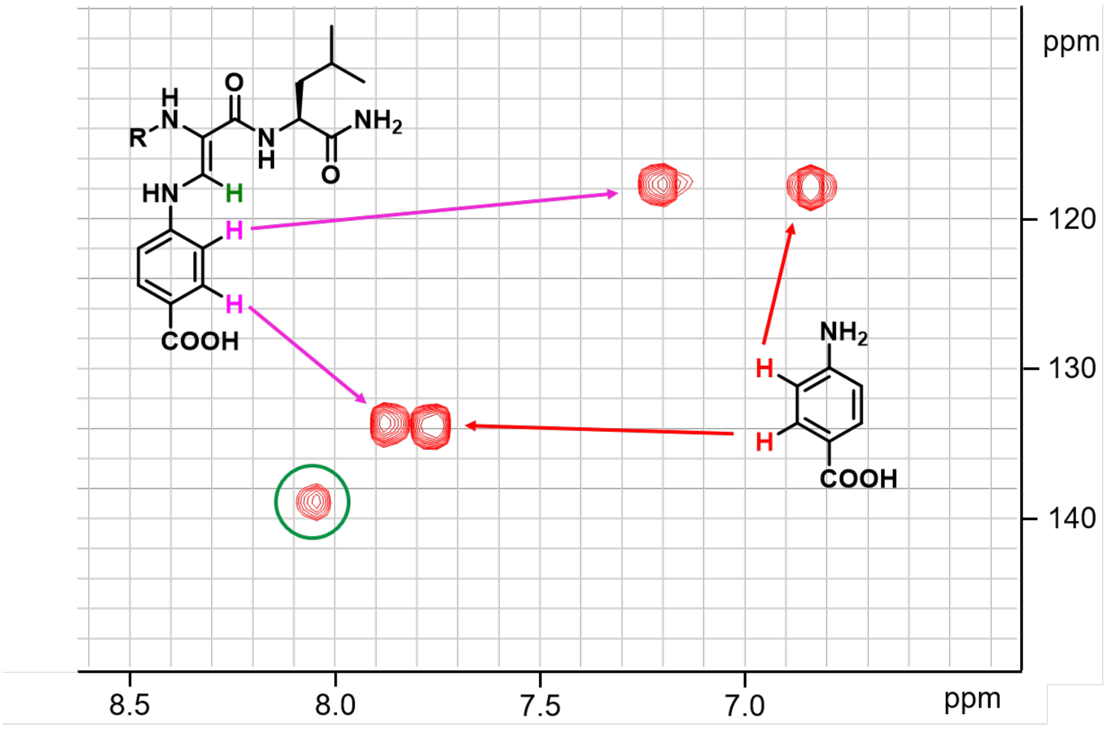

Supplementary Figure 19. HMQC-NMR NMR spectrum of **9** (5 mM) with 4-aminobenzoic acid (10 mM) in 9:1 H_2O/D_2O, 300 K. The enamine product is characterized by the crosspeak (green) between the Cβ of the formyl glycine residue and the β-proton.

Reaction of Formylglycin-Peptide 3 with DTT

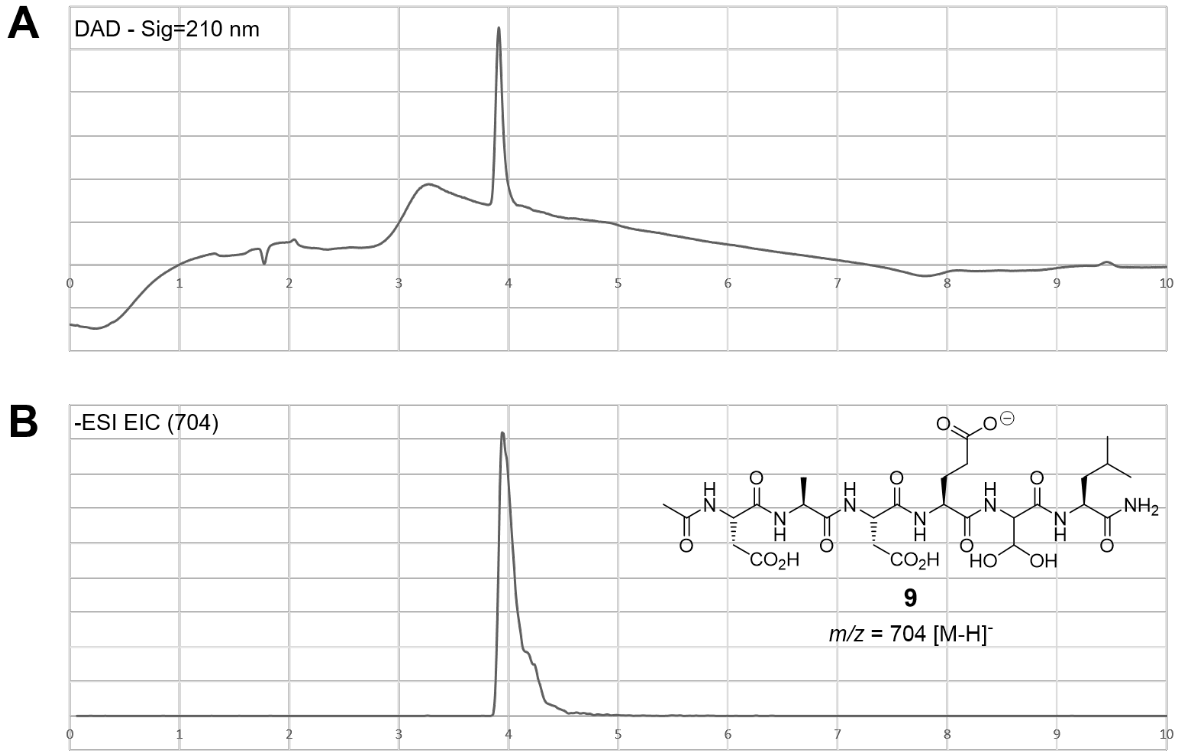

Supplementary Figure 20. HPLC chromatogram of fG peptide **9**, A) 210 nm UV trace, B) extracted ion chromatogram for 704 *m/z* (mono-anion of fG hydrate **9**).

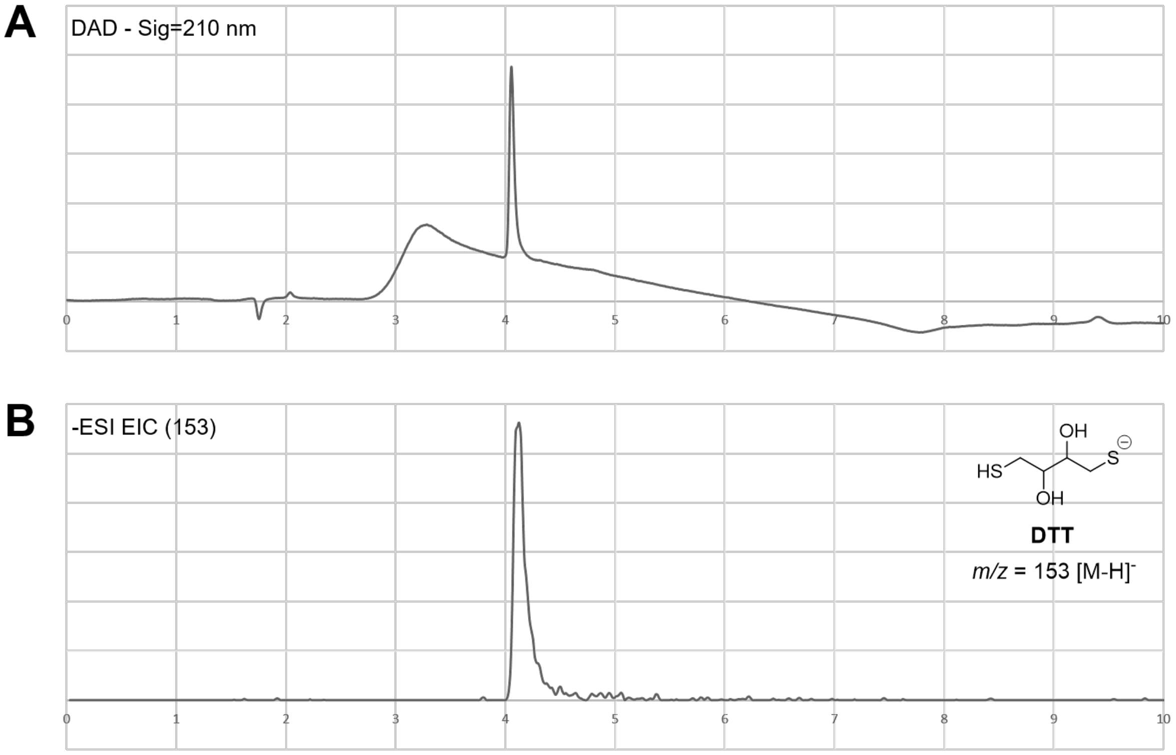

Supplementary Figure 21. HPLC chromatogram of 1,4-dithio-threitol (DTT), A) 210 nm UV trace, B) extracted ion chromatogram for 153 *m/z* (mono-anion of DTT).

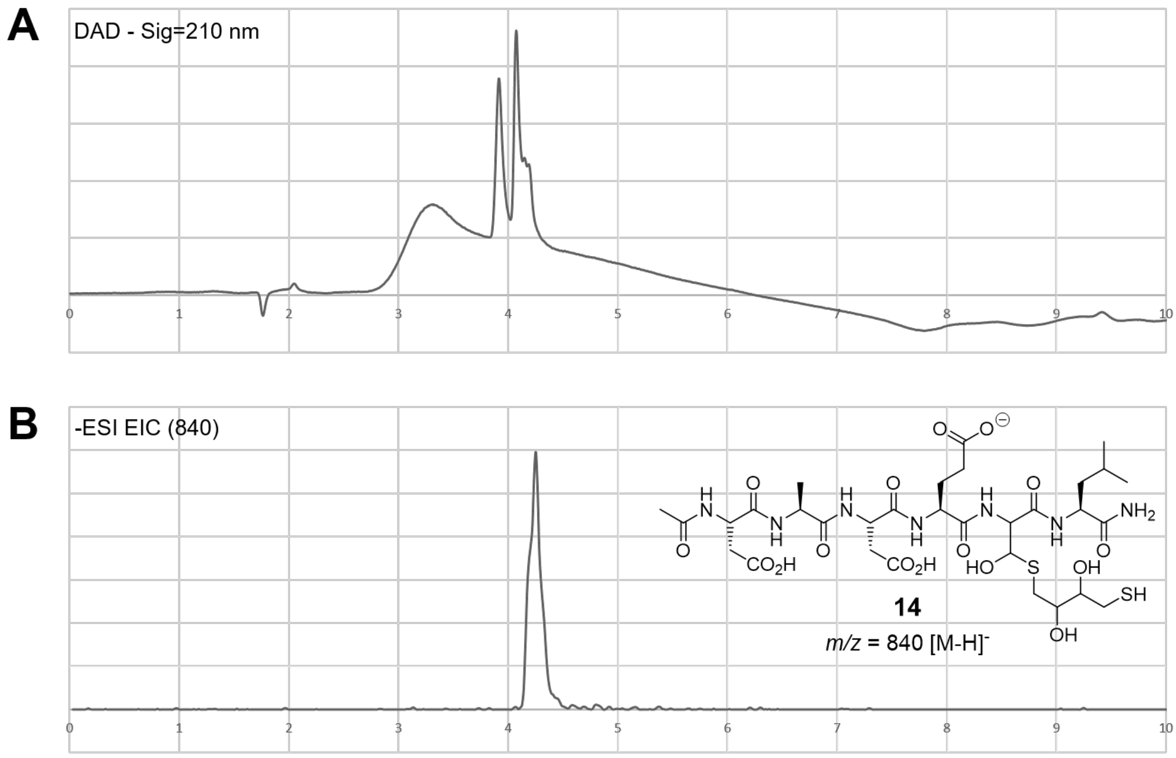

Supplementary Figure 22. HPLC chromatogram of fG-peptide **3** (2.5 mM) with 1,4-dithio-threitol (DTT) (12.5 mM) after 10 min at RT in water, A) 210 nm UV trace, B) extracted ion chromatogram for 840 *m/z* (mono-anion of the hemi-thioacetal product **14**).

Fragments Tested in Fragment Ligation Experiments (Selection)

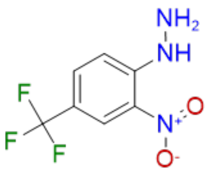

STK731597, (2)

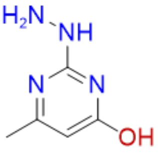

STK261625, (8)

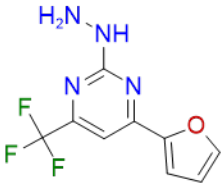

STK298600, (16)

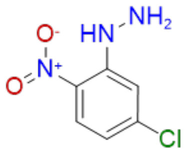

STK002063, (17)

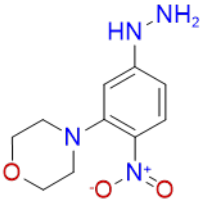

STK003188, (18)

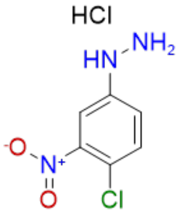

STK662472, (30)

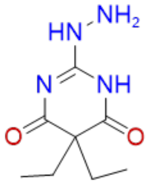

STK257524, (36)

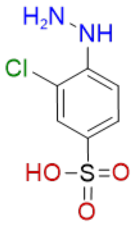

STK379058, (57)

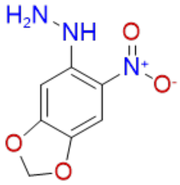

STL119725, (62)

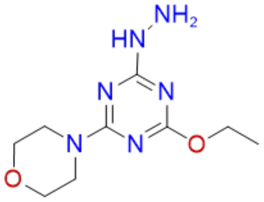

STK682887, (66)

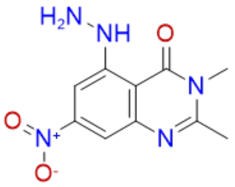

STK689486, (68)

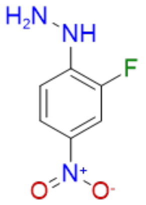

STK694376, (71)

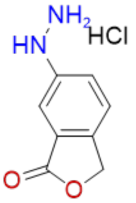

STK694732, (73)

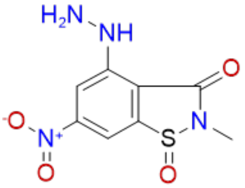

STK763014, (74)

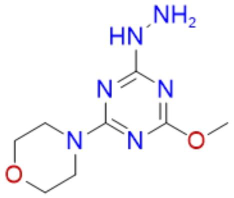

STK772658, (78)

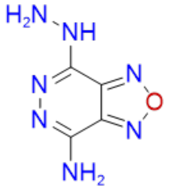

STK785161, (80)

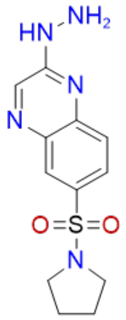

STK902133, (83)

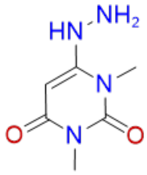

STL122221, (93)

Supplementary Figure 23. Selection of tested primary amino/hydrazine aromatic fragments part 1.

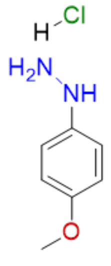

ID 359

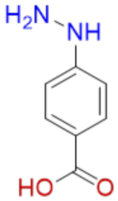

ID: 455

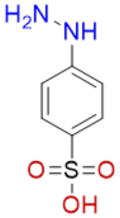

ID: 458

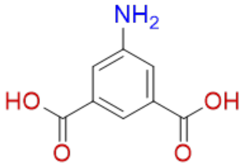

ID: 519

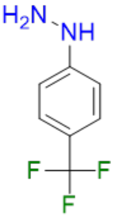

ID: 602

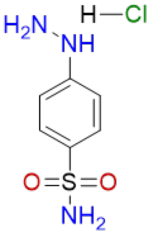

ID: 685

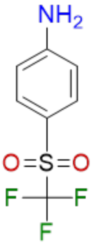

ID: 729

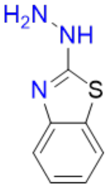

ID: 731

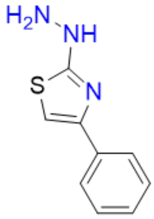

ID: 1498

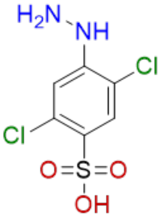

ID: 1499

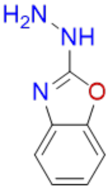

ID: 1537

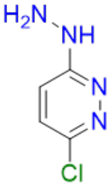

ID: 1590

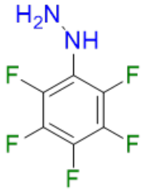

ID: 1591

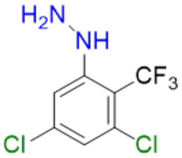

ID: 1592

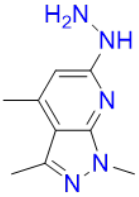

ID: 1604

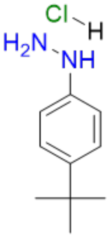

ID1605

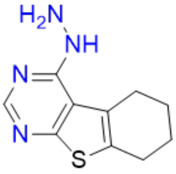

ID1606

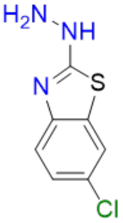

ID1607

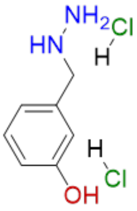

ID1609

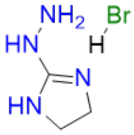

ID1610

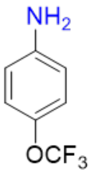

ID: 2067

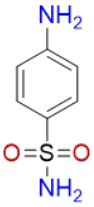

ID: 2068

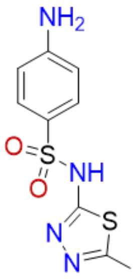

ID: 2069

Supplementary Figure 24. Selection of tested primary amino/hydrazine aromatic fragments part 2.

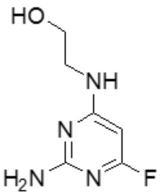

300087

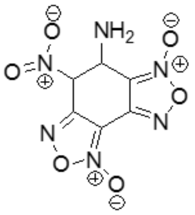

300192

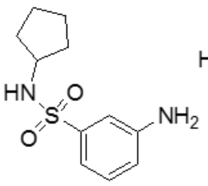

300380

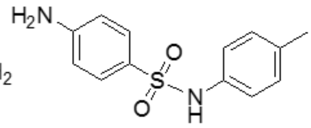

300456

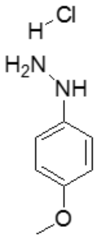

359

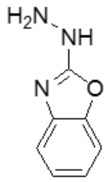

1537

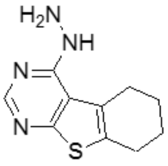

1606

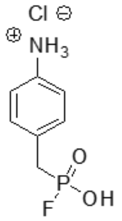

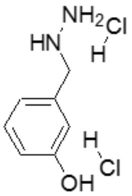

1609

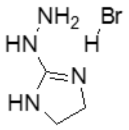

1610

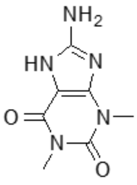

300177

Supplementary Figure 25. Selection of tested primary amino/hydrazine aromatic fragments part 3.

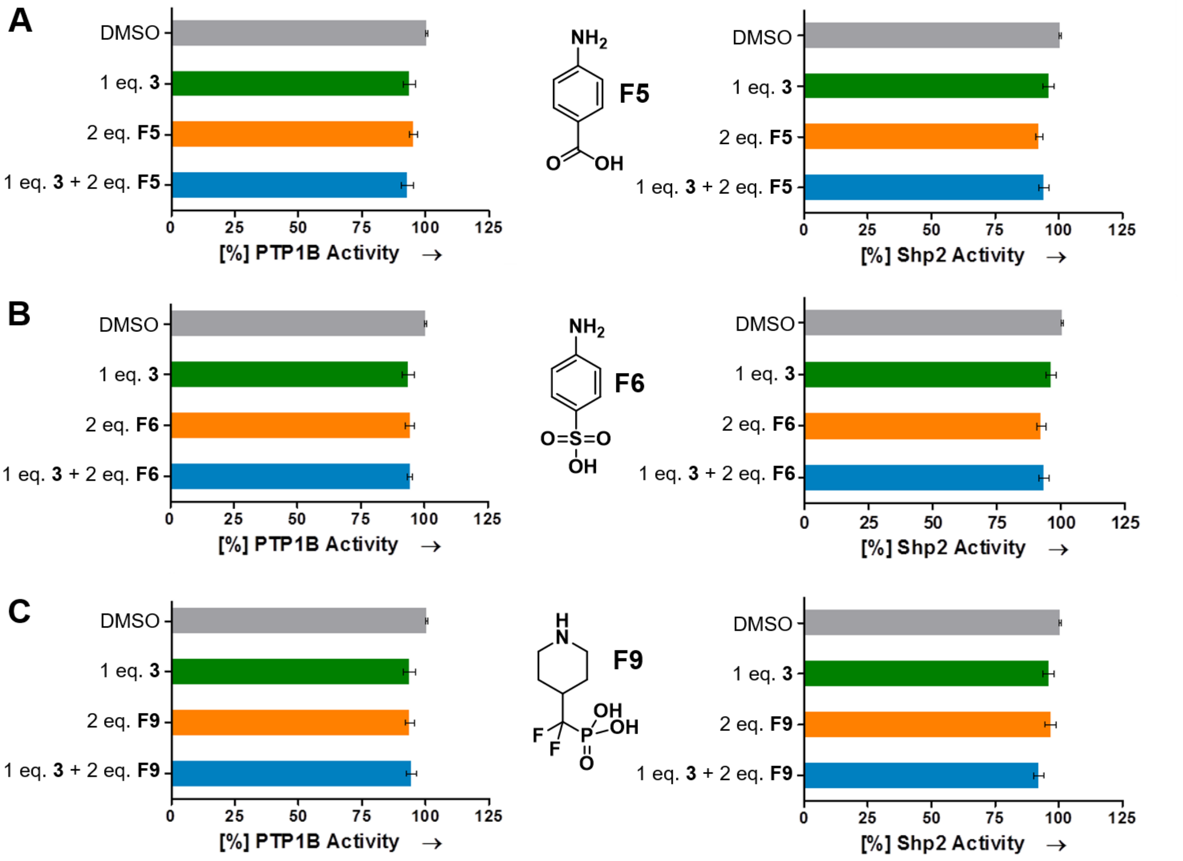

Supplementary Figure 26. Results of dynamic ligation assay of peptide **3** with fragments **F5**, **F6**, and **F9** for PTP1B and SHP2.

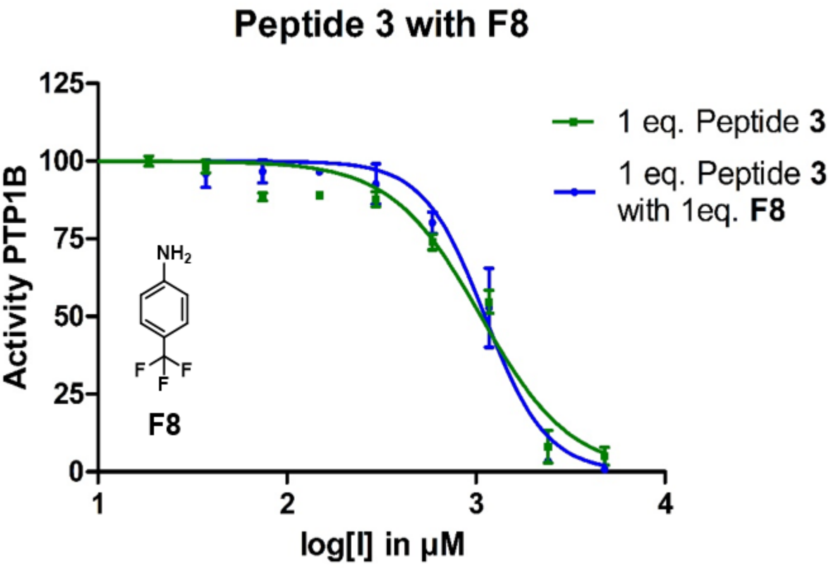

Supplementary Figure 27. Inactive fragment ligation product of 4-amino-phenyl-trifluoromethane **F8**.

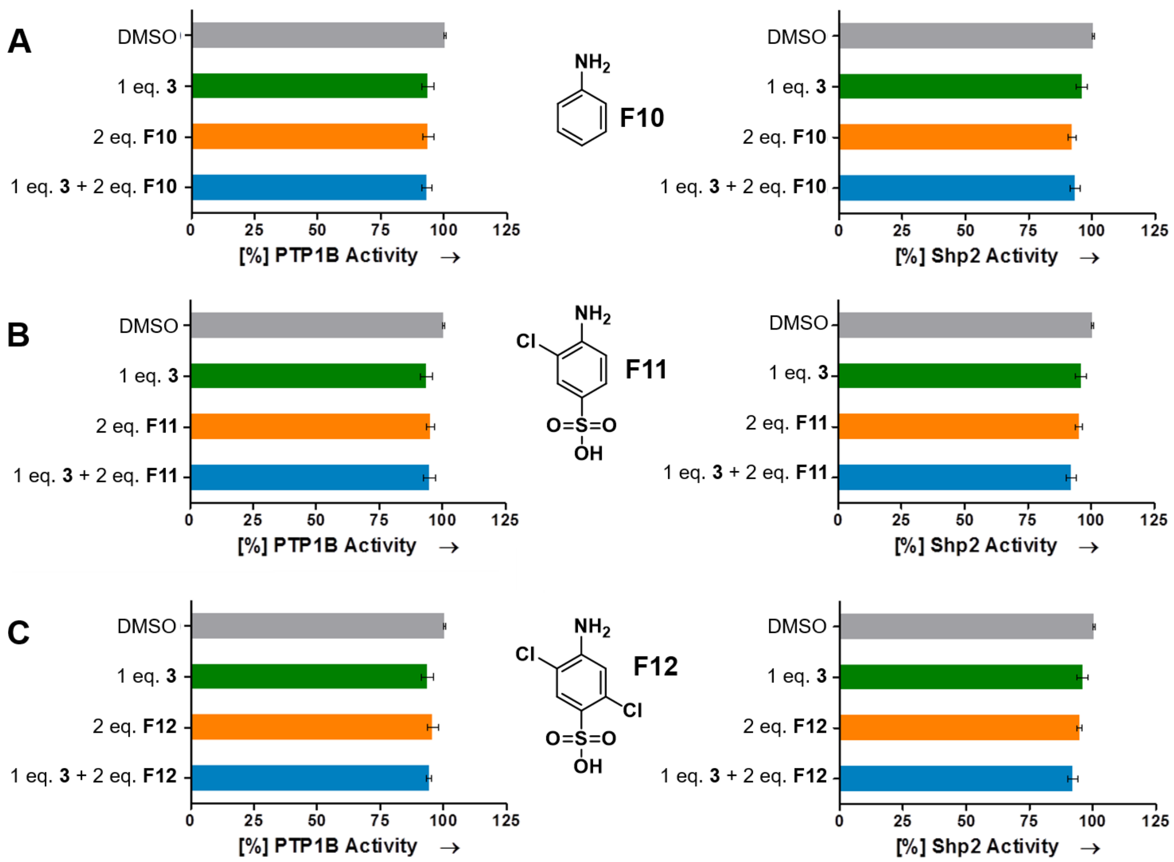

Supplementary Figure 28. Results of dynamic ligation assay of peptide **3** with fragments **F10**, **F11**, and **F12** for PTP1B and SHP2.

Table 1. Inhibition constants of hydrazine fragments **F3** and **F13**-**F17** toward PTP1B and SHP2.

#	Compound	*IC₅₀* (μM) PTP1B	*IC₅₀* (μM) SHP2
**F3**	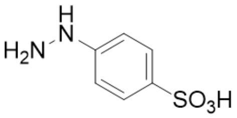	34 ± 3	> 250
**F13**	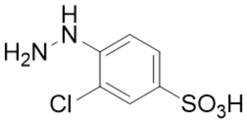	29 ± 3	74 ± 9
**F14**	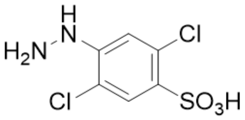	> 250	4.3 ± 0.9
**F15**	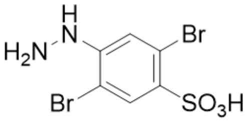	244 ± 10	77 ± 9
**F16**	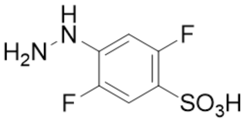	> 250	12.6 ± 4.4
**F17**	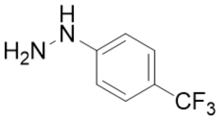	> 250	> 250

[

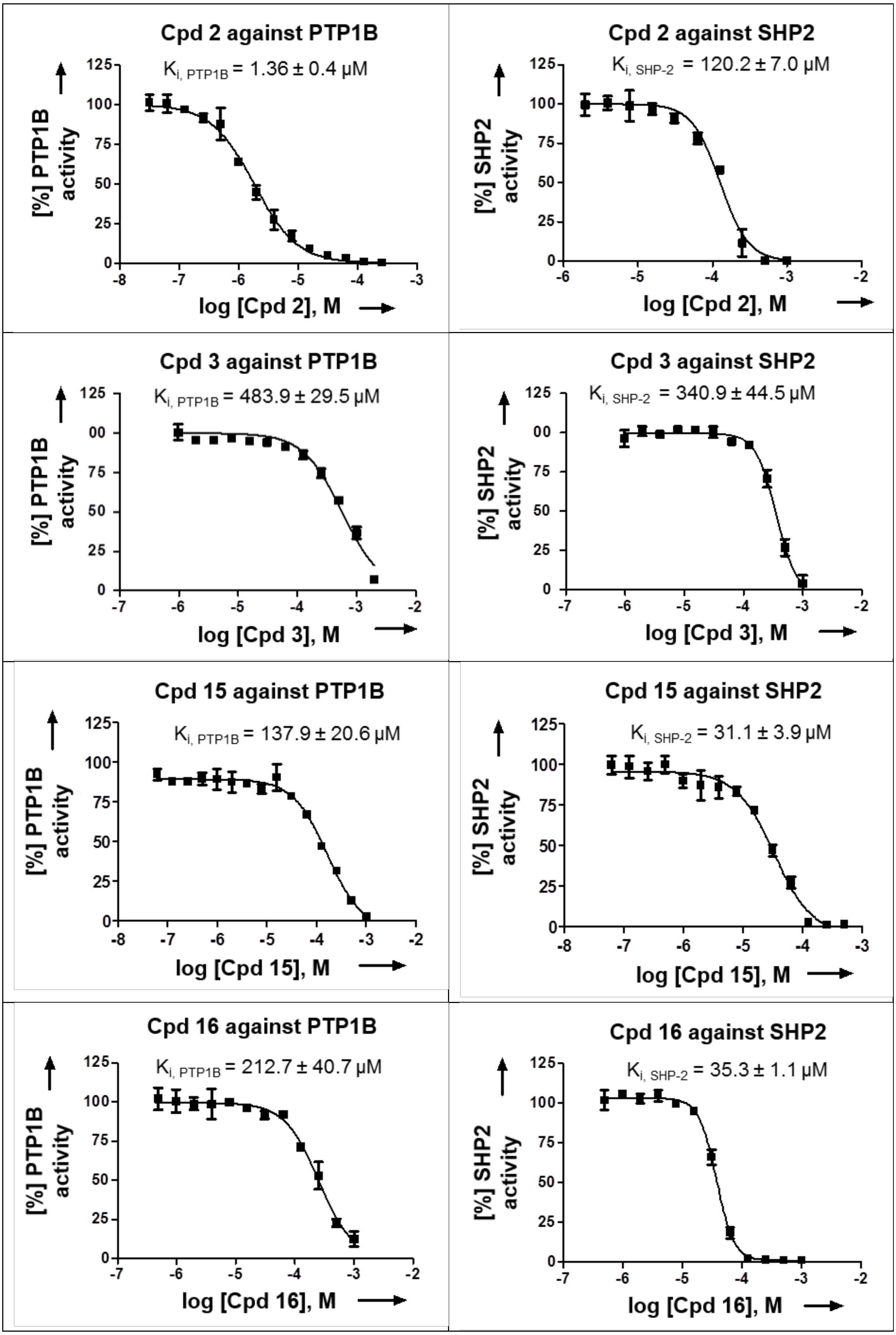

Supplementary Figure 29. *IC_{50}*-Curves and resulting *K_I*-values calculated with Cheng-Prusoff equation; error in SD

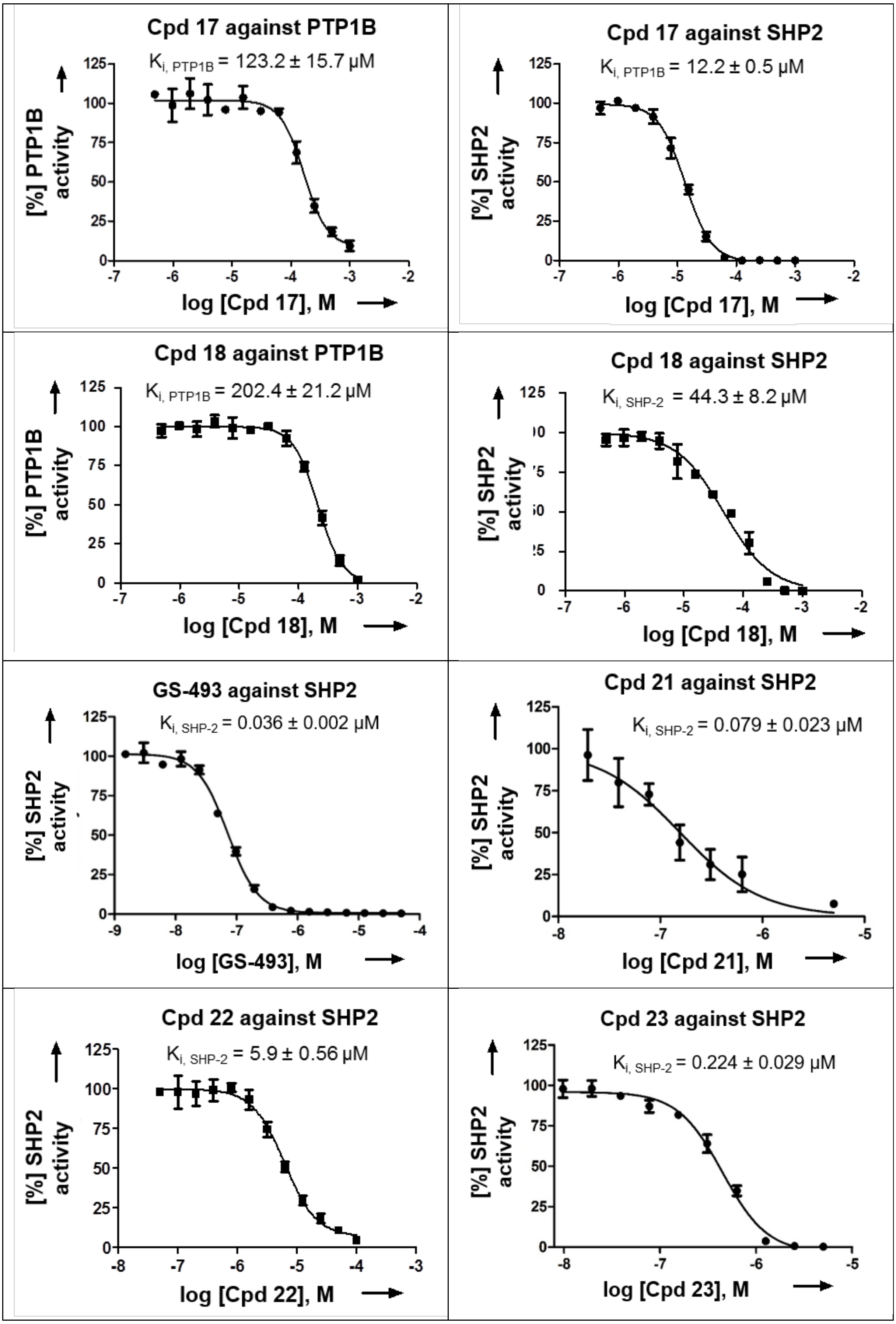

Supplementary Figure 30. *IC_{50}*-Curves and resulting *K_I*-values calculated with Cheng-Prusoff equation; error in SD

Determination of K_I values using Michaelis-Menten kinetics

The assays for the determination of K_I values were performed as described under the same conditions as mentioned in the main paper. The initial velocities of the enzymatic reaction inhibited by compound **21** were determined using four different inhibitor concentrations (0, 75, 100 and 150 nM) and four different substrate concentrations (100, 50, 25 and 12.5 µM). Fluorescence was monitored over 10 min, during which the increase in fluorescence was linear. Initial velocities were calculated and were plotted as a function of the substrate concentration. The inhibition constant K_I was determined using Michaelis-Menten kinetics applying GraphPad Prism 9 (Supplementary Figure 31). The obtained data were fitted to three different inhibition models, namely competitive, uncompetitive, and non-competitive inhibition. The best fit was obtained for the competitive inhibition mode, resulting in the highest correlation coefficient and indicating that compound **21** acted as a competitive inhibitor (Supplementary Table 2).

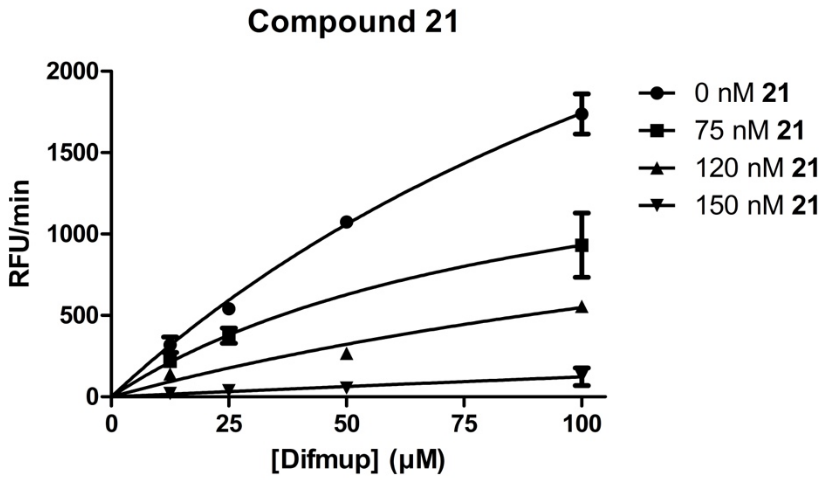

Supplementary Figure 31. Michaelis-Menten plots of compound **21** using inhibitor concentrations of 0, 75, 100 and 150 nM and Difmup concentrations of 100, 50, 25 and 12.5 µM, respectively.

Table 2. Results of Michaelis-Menten plots for different modes of inhibition of compound **21** (competitive, uncompetitive and non-competitive inhibition).

Inhibition model	Competitive	Uncompetitive	Non-competitive
K_M (µM)	189.7	351.9	220.9
K_I (nM)	32.01	8.4	46.54
R^2	0.9007	0.8637	0.8991

Computational Methods and Docking Experiments

Table 3. Structures of the ligation products of **3** with fragments docked to PTP1B (1PTU).

**3**	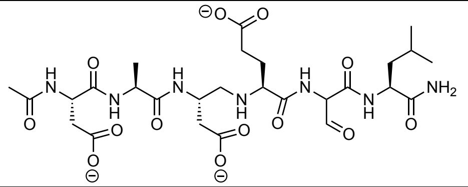	**9**	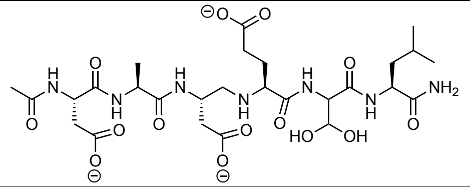
**12**	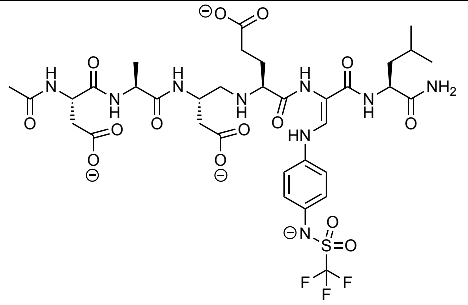	**18**	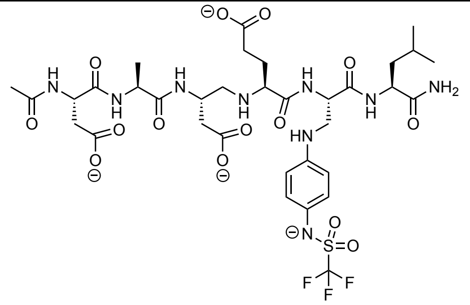
**11**	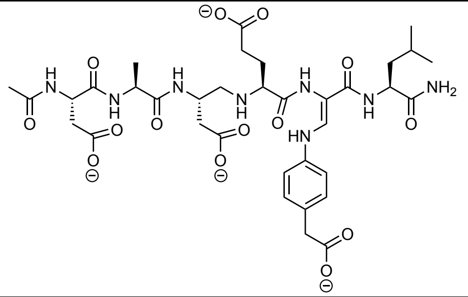	**15**	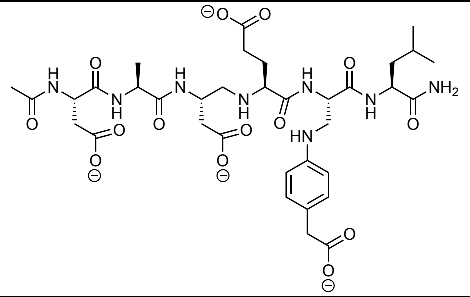
**L1**	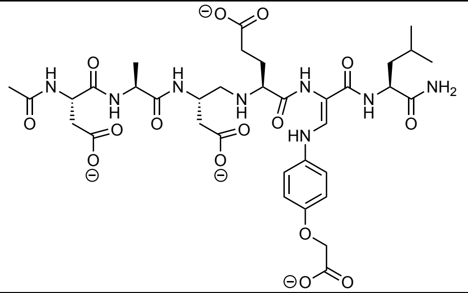	**16**	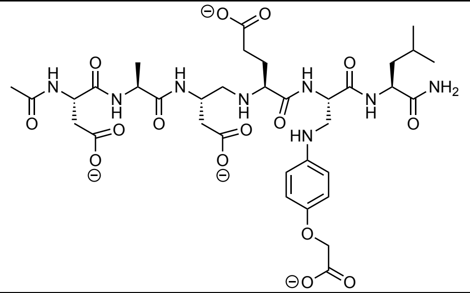
**L2**	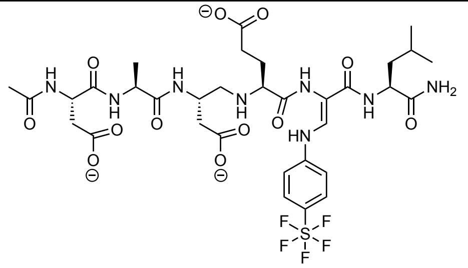		

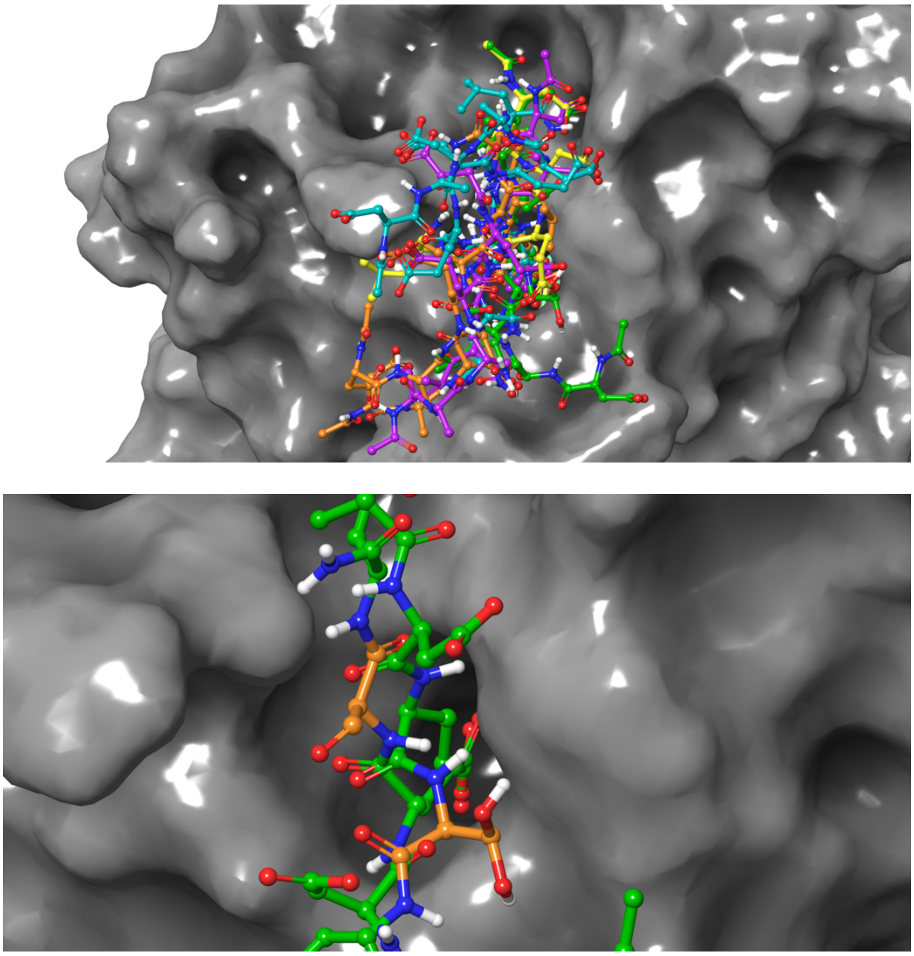

Supplementary Figure 32. Top: Peptide backbone positions from docking calculations of all ligation products shown in **Table 2**. Each peptide is colored in a different color. Bottom: fGly peptide **3** and hydrate **9** docked to PTP1B. The peptides are colored in green and the hydrated or non-hydrated fGly residue is colored in orange.

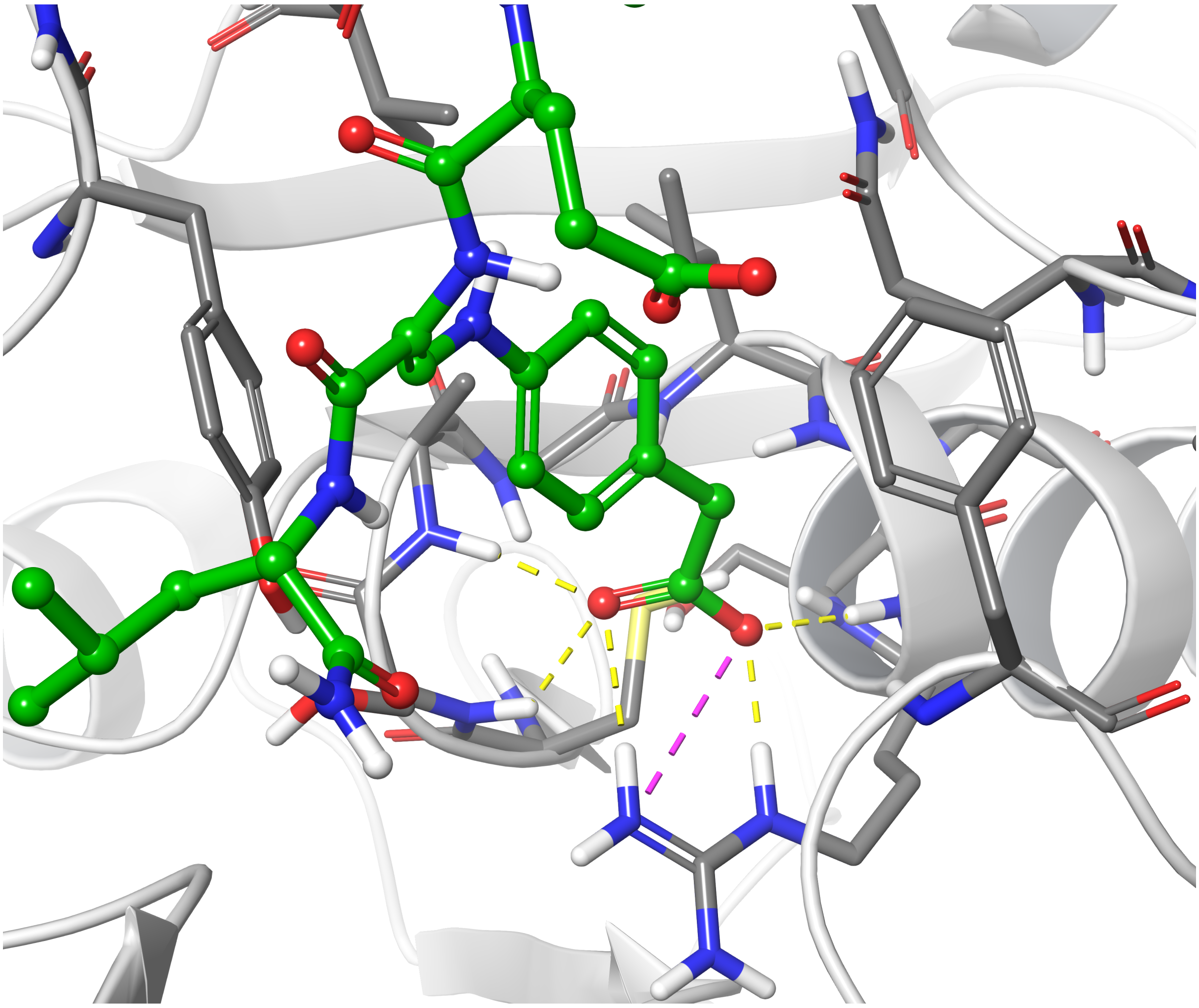

Supplementary Figure 33. Docking of ligation product **11** to the active site of PTP1B. N-H-O hydrogen bonds are assigned in yellow and Coulomb interactions in magenta.

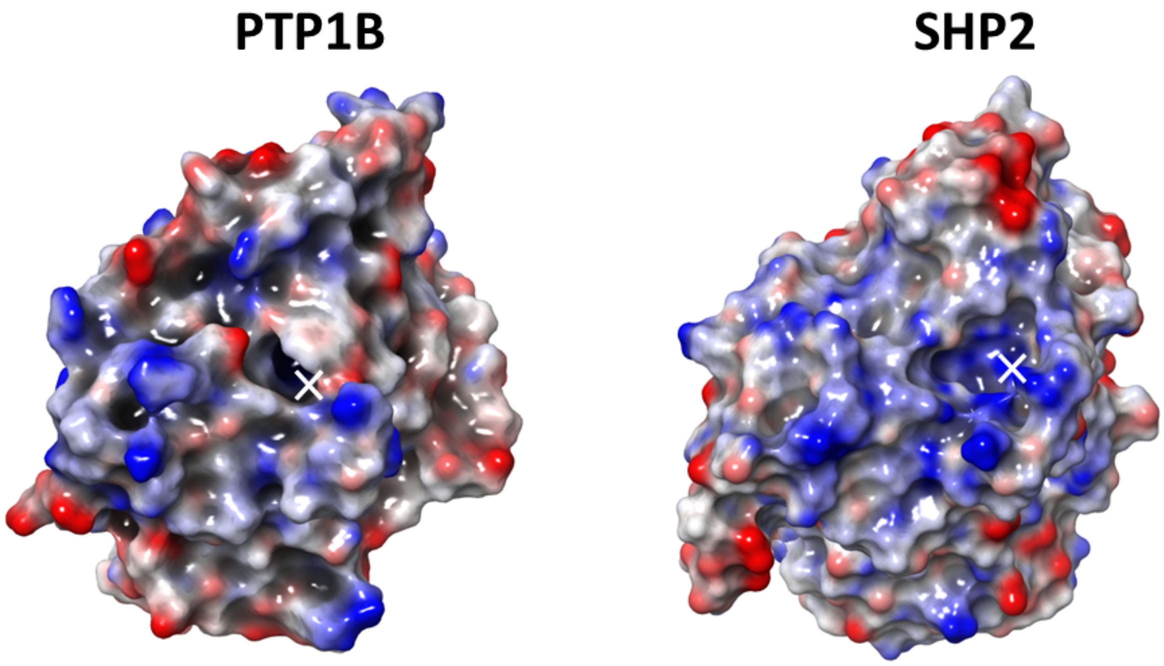

Supplementary Figure 34. Electrostatic surfaces of PTP1B and SHP2. The white X marks the active site (more specifically the head group of Arg221).

Docking of Pyrazolones

The deprotonated structures of **GS-493**, **21** and **23** were docked to the receptor SHP2 (pdb: 3ZM3). The hydrogen bond between the carbonyl oxygen of the pyrazolone moiety and the aryl NH was enforced to stay in place by dihedral constraints on this region of the ligands throughout the docking. The preferred docking pose (fig. SI-XX) of all of these three structures places the phosphotyrosine-mimetic fragment into the main binding pocket. Here, the oxygens of the headgroup interact, through hydrogen bonds or salt-bridges with the sidechains and backbone NH of Arg465 and Ser460. The carboxyl oxygen of **23** also forms a hydrogen bond to the backbone NH of Gly427. Moreover, the three structures show a similar orientation in their preferred binding poses with one of the Ph-NO_2 rings pointing towards the loop of Asn281 and the other ring pointing to the proximity of the sidechains of Arg362, Lys364 and Lys366, where salt-bridges can be formed between the positively charged sidechains and the nitro moiety.

The structure of **22** was also docked with the same specifications but did not place the phosphotyrosine-mimetic fragment into the main pocket and was therefore excluded from this analysis.

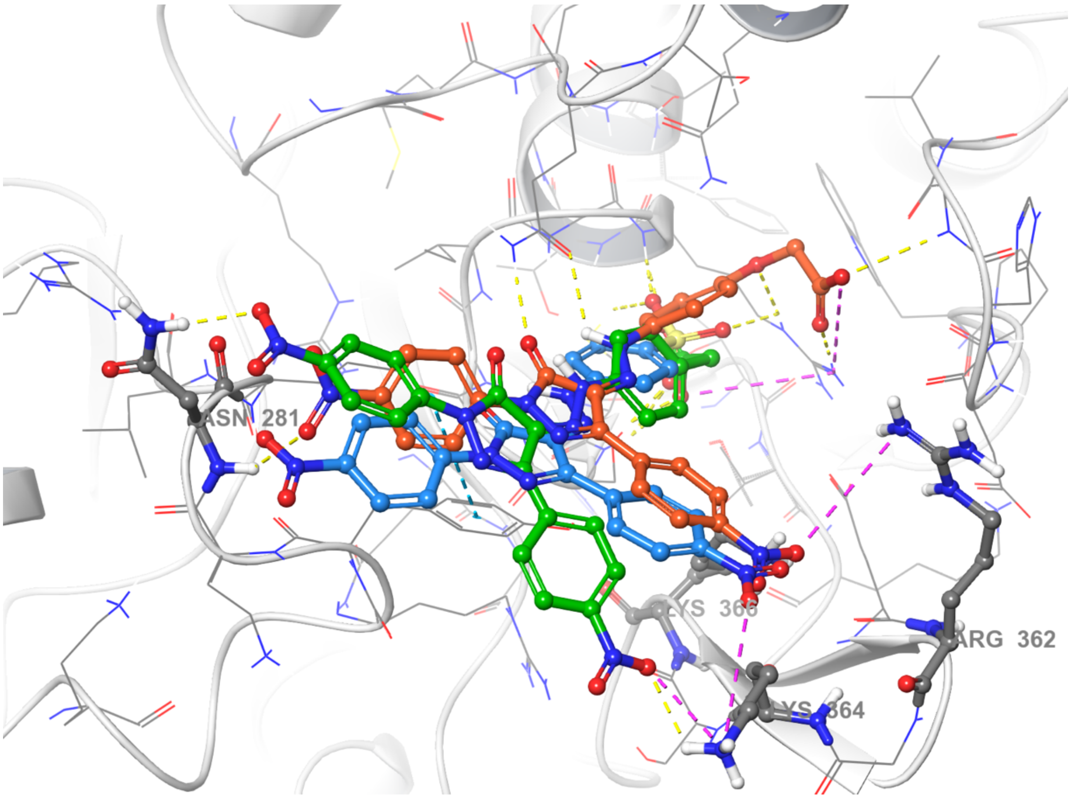

Supplementary Figure 35. Docking pose of **GS-493** (blue), **21** (green) and **23** (orange) at the SHP2 binding site. Ligand-receptor interactions are indicated. Hydrogen bonds are shown in yellow, salt-bridges in purple and pi-pi interactions in cyan.

Supporting Chemical Methods and NMR Spectra

Ac-DADE-(P-Y)-L-NH$_2$ (1).

Phosphopeptide **1** was prepared following the procedure described for peptide **3** in this paper using the amino acid N-Fmoc-*O*-benzyl-*O*-phospho-tyrosine as a building block.

Ac-DADE-(PDFM-F)-L-NH$_2$ (2)

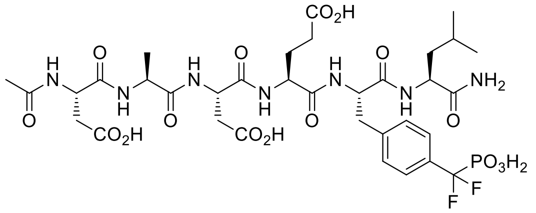

Following the procedure described for peptide **3** in this paper, using the unnatural amino acid N-Fmoc-(4-phosphono-difluoromethyl)-phenylalanine (PDFM-Phe, PDFM-F), **2** was obtained as a white solid 300 mg preloaded resin (0.4 mmol/g) were used for cleavage. The peptide was purified by column chromatography to provide **2** as white solid (29.6 mg, 41%); **HRMS:** (ESI): $C_{34}H_{48}F_2N_7O_{16}P$ [M], 879.2863 Da. calcd *m/z* 880.2941 $[M+H]^+$, 902.2761 $[M+Na]^+$, found *m/z* 880.2939 $[M+H]^+$, 902.2758 $[M + Na]^+$.

2,5-Bis(4-nitrophenyl)-2,4-dihydro-3H-pyrazol-3-one (X1)

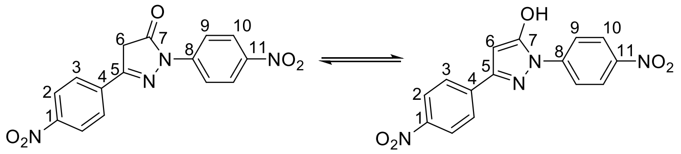

4-Nitrophenylhydrazine (1.136 g, 7.59 mmol, 1.3 eq.) and Ethyl 4-nitrobenzoylacetate (1.5 g, 6.33 mmol, 1.0 eq.) were stirred in AcOH (10 mL) at 120 °C for 18 h. H_2O (20 mL) was added to the mixture and the aqueous phase was extracted with EtOAc (3 x 30 mL). The combined organic phases were washed with sat. aq. $NaHCO_3$ (20 mL) and Brine (20 mL), dried over Na_2SO_4. The crude product was concentrated under reduced pressure and purified by column chromatography to provide **X1** as yellow solid (1.86 g, 90%); R_f = 0,68 (dichloromethane/methanol, 9:1).

^{1}H-NMR: (300 MHz, DMSO-*d$_6$*): Enol: δ = 12.50 (1H, br. s, H-7), 8.33 (2H, d, *J* = 9.3 Hz, H-10), 8.24 (2H, d, *J* = 8.9 Hz, H-2), 8.09 (2H, d, *J* = 9.3 Hz, H-9), 8.08 (1H, d, *J* = 8.9 Hz, H-3), 6.20 (1H, br. s, H-6) ppm. **^{13}C-NMR** (75 MHz, DMSO-*d$_6$*): Enol: δ = 155.9 (C-7), 149.3 (C-5), 147.0 (C-1), 144.2 (C-11), 143.7 (C-8), 139.0 (C-4), 126.3 (C-3), 124.9 (C-10), 123.9 (C-2), 120.4 (C-9), 86.7 (C-6) ppm. **HRMS:** (ESI): $C_{15}H_{10}N_4O_5$ [M], 326.0651 Da. calcd *m/z* 325.0573 $[M-H]^-$, 327.0729 $[M+H]^+$, found *m/z* 325.0569 $[M-H]^-$, 327.0725 $[M+H]^+$.

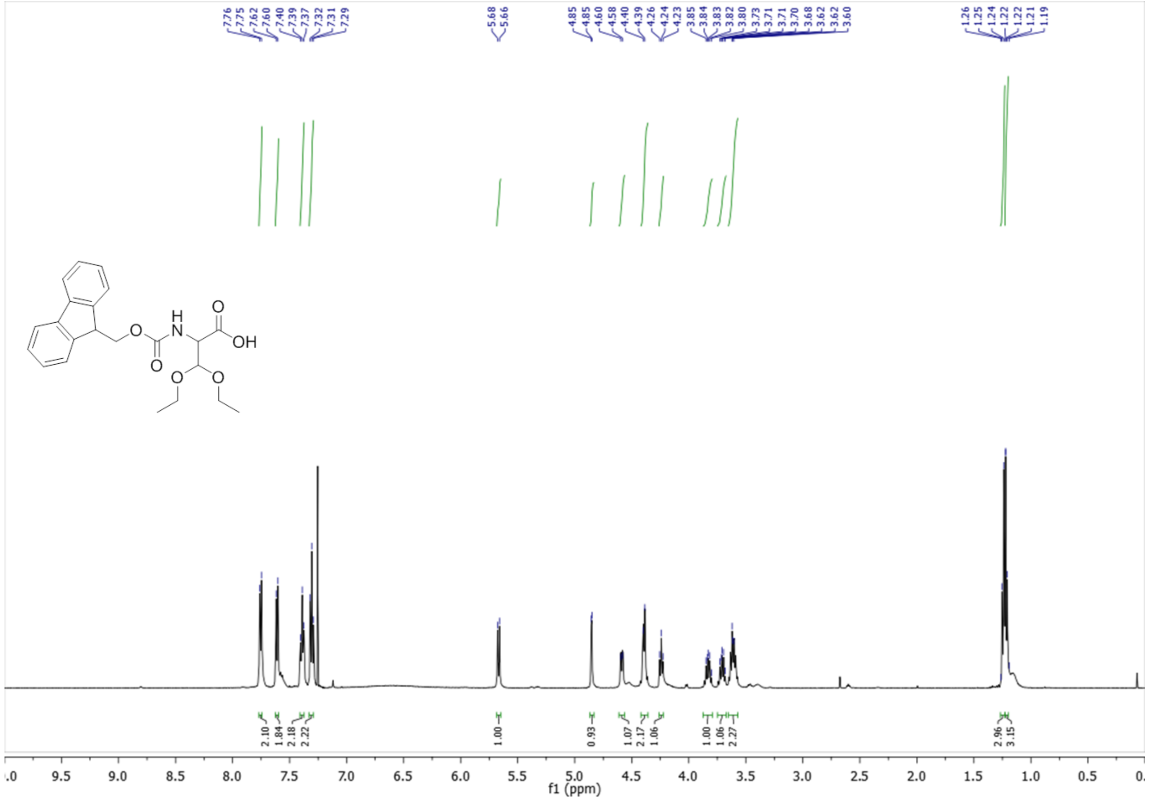

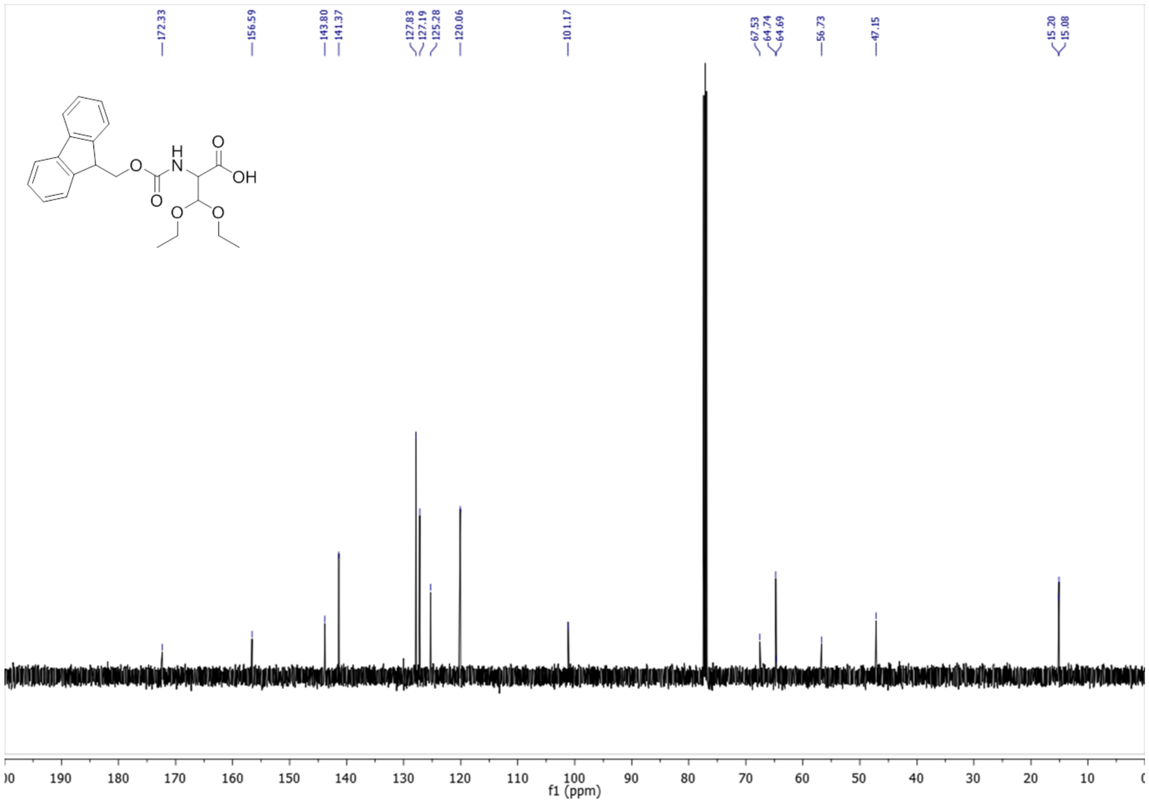

Supplementary Figure 36. ^{1}H- und ^{13}C-Spectra of **4** (500 MHz, $CDCl_3$)

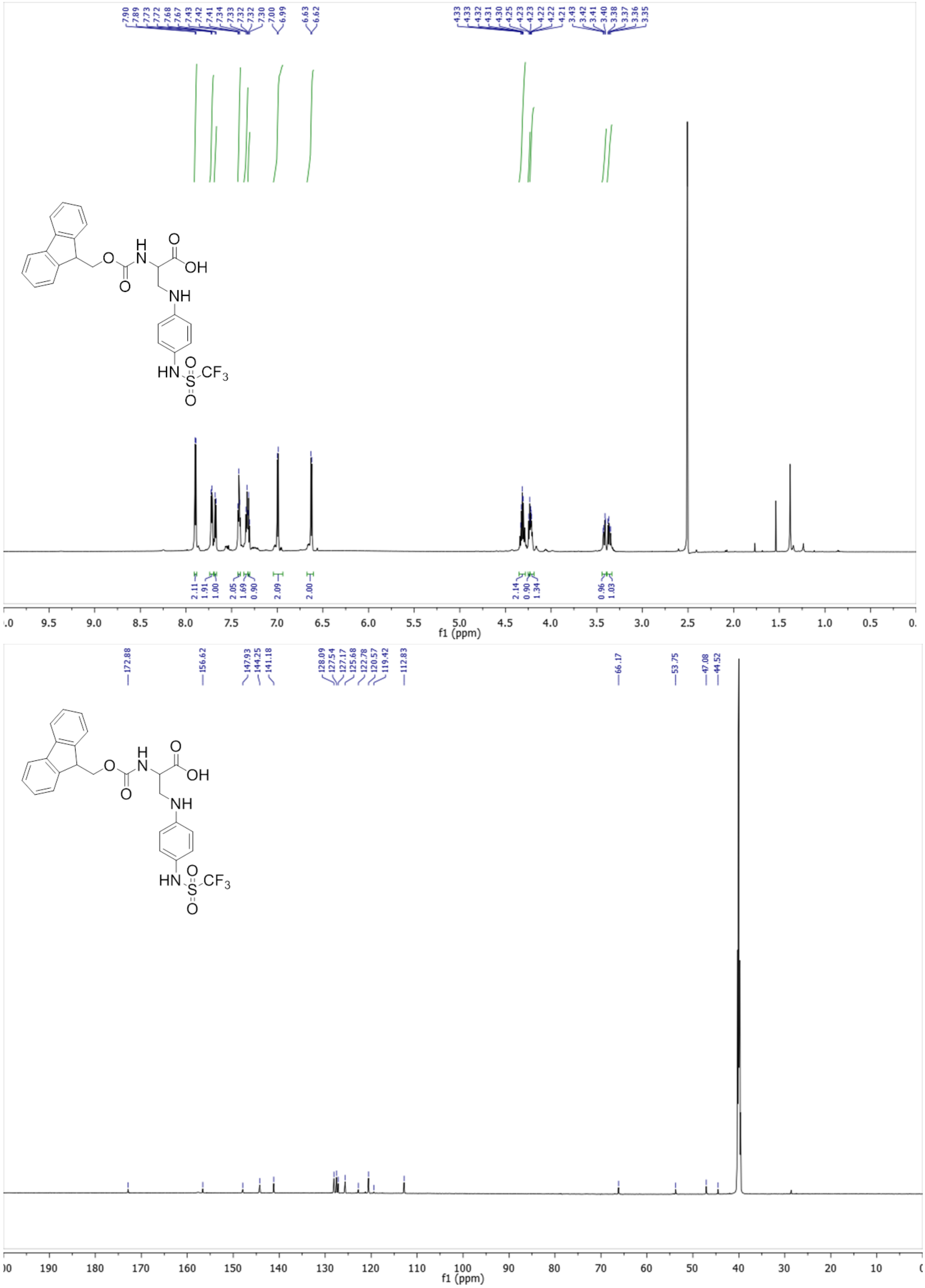

Supplementary Figure 37. ^{1}H- und ^{13}C-Spectra of **19** (700 MHz, DMSO-d_6)

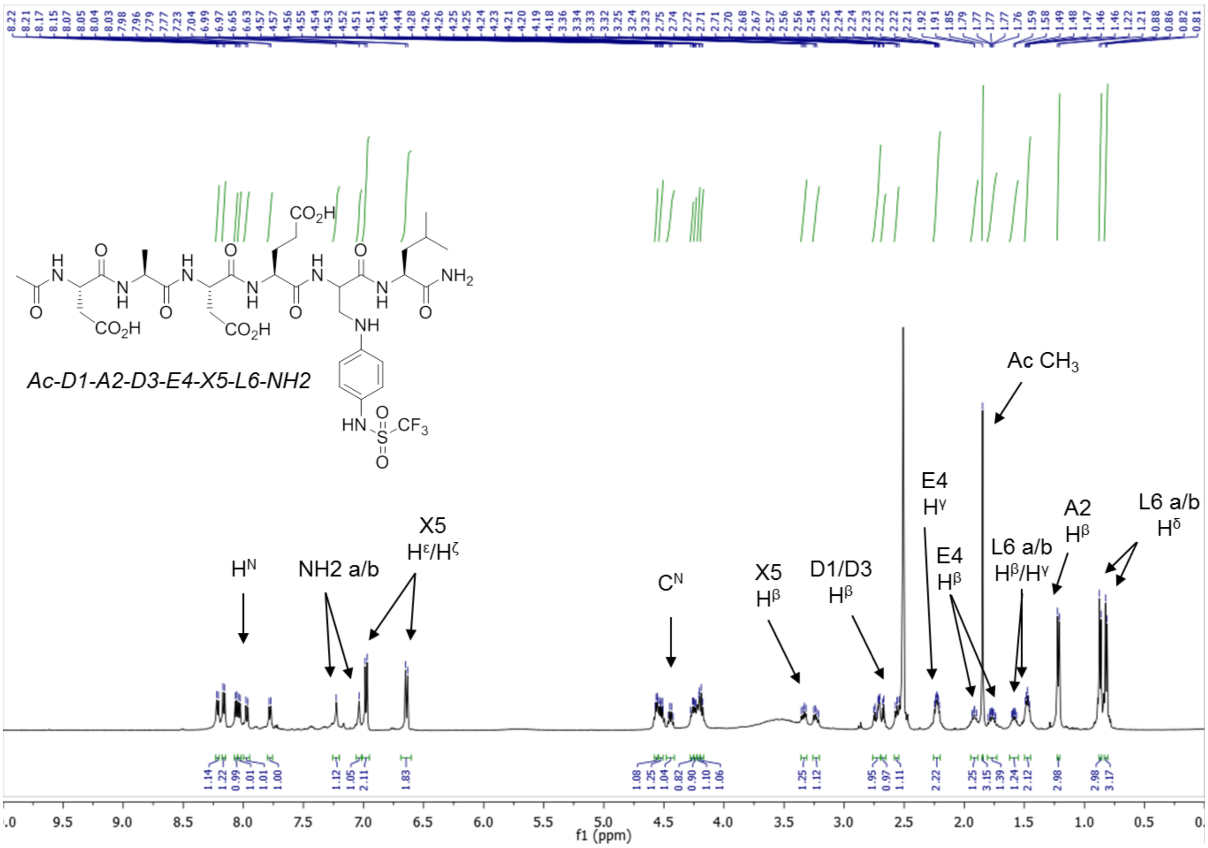

.

Supplementary Figure 38. ^{1}H-Spectra of **18** (500 MHz, DMSO-d6)

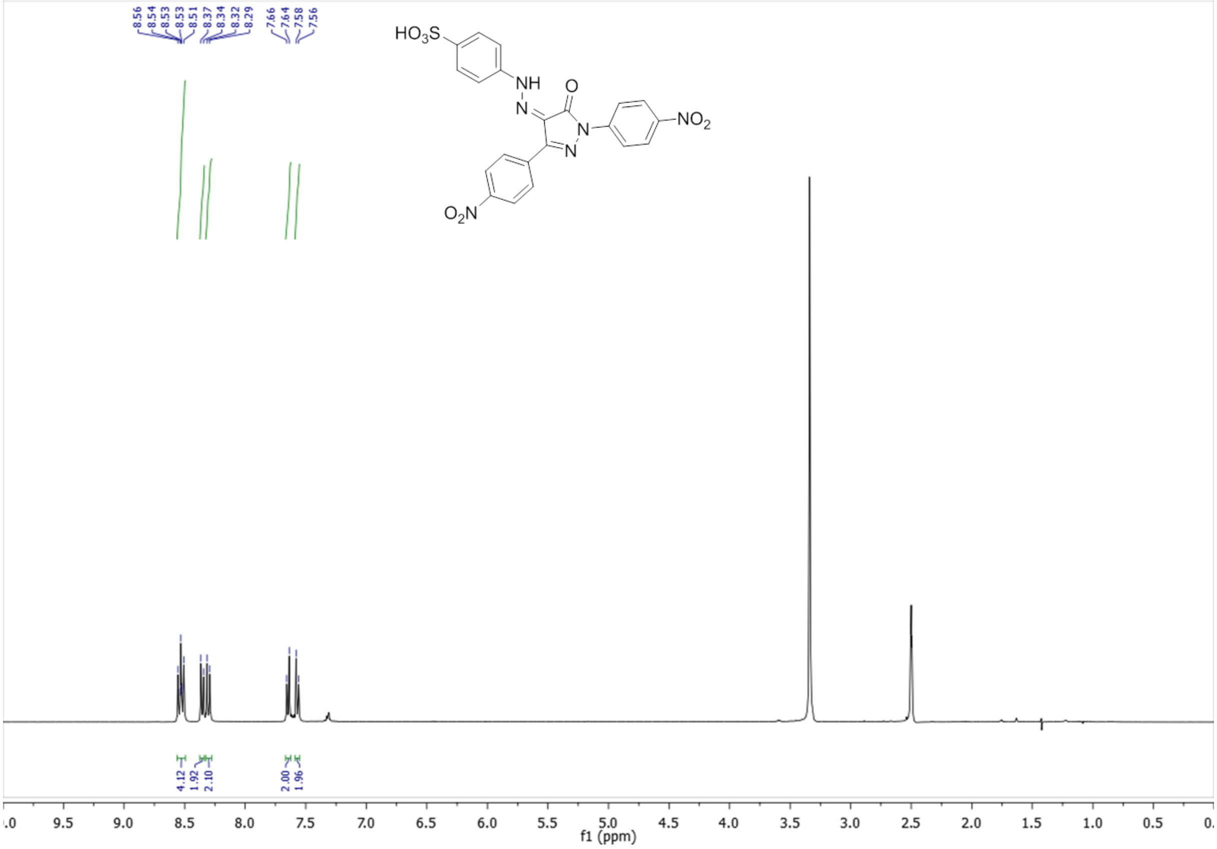

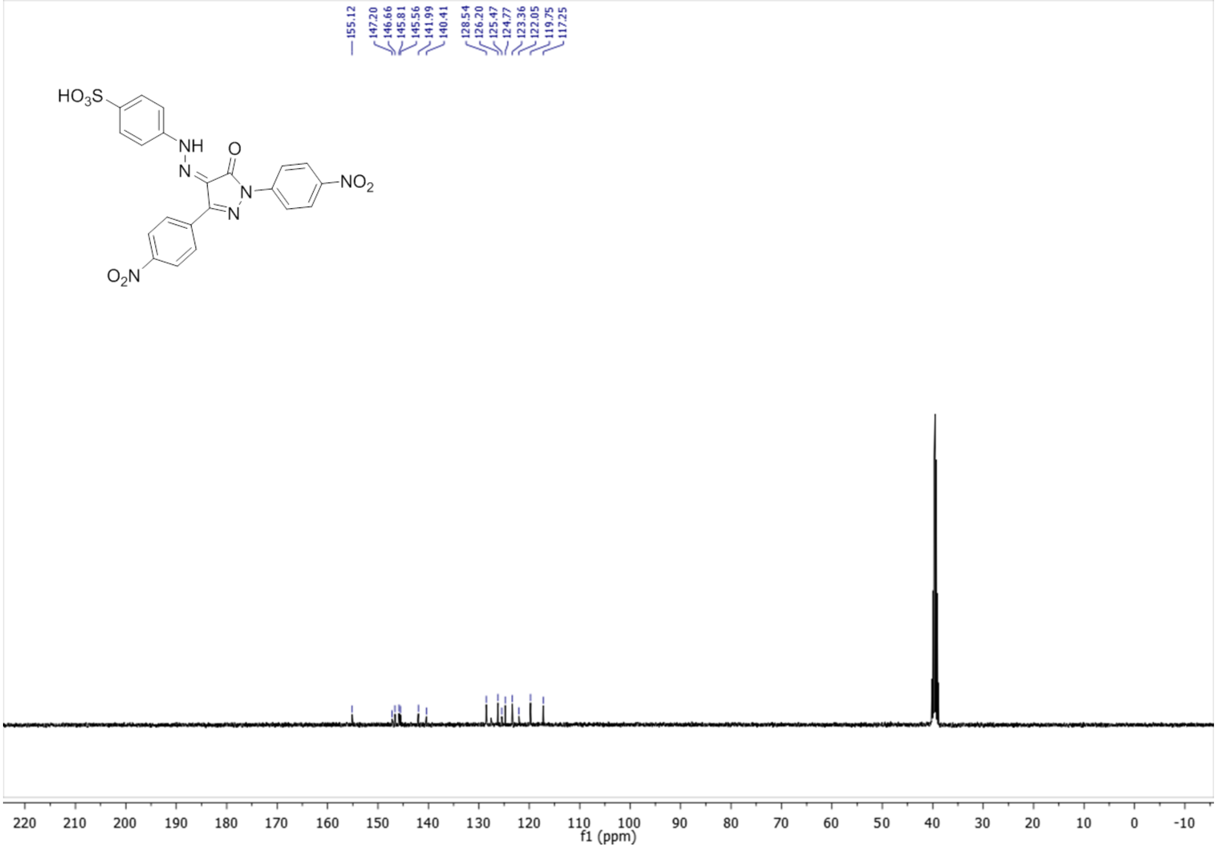

Supplementary Figure 39. ^{1}H- und ^{13}C-Spectra of **GS-493** (500 MHz, DMSO-d_6)

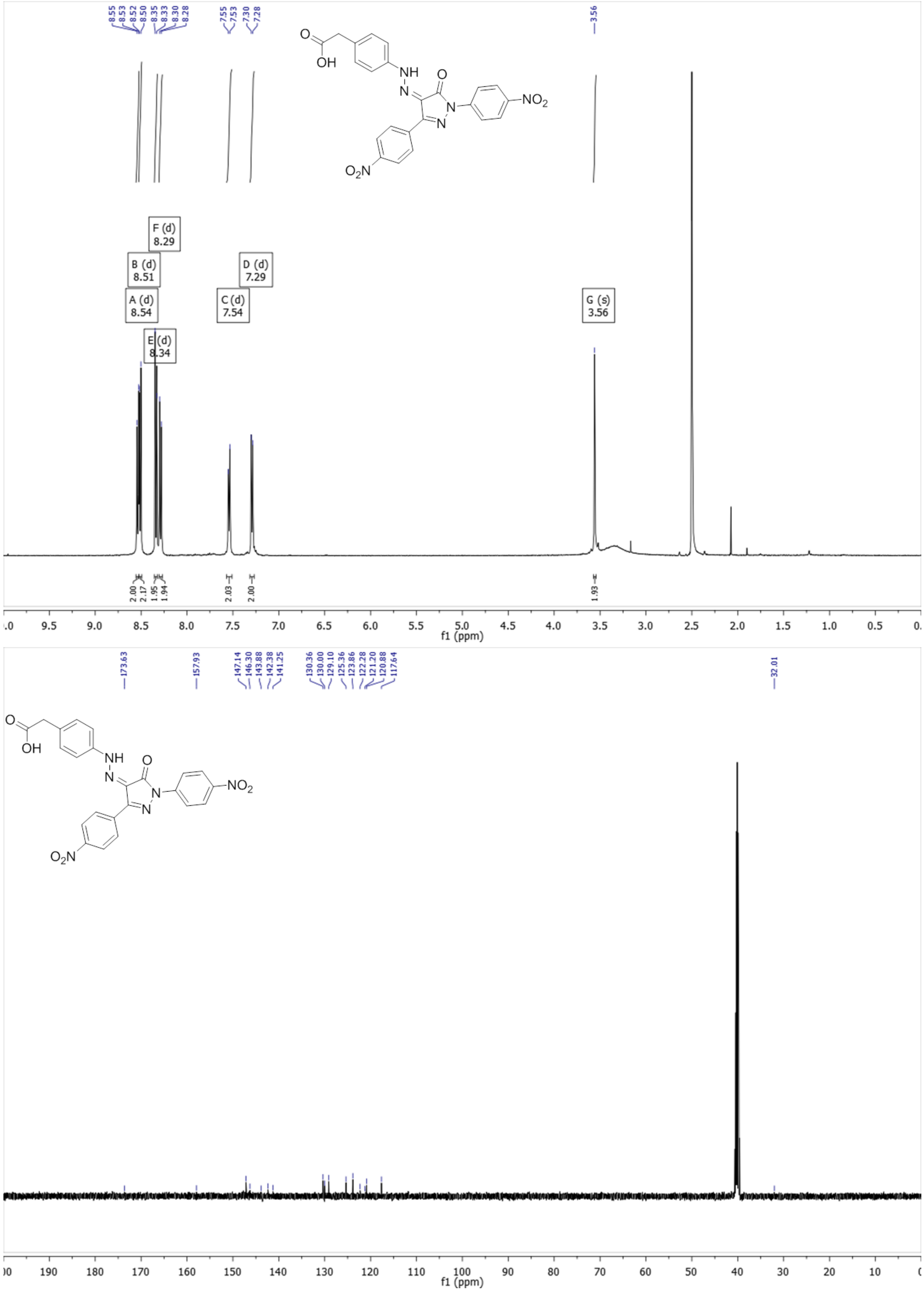

Supplementary Figure 40. ^{1}H- und ^{13}C-Spectra of **21** (500 MHz, DMSO-d_6)

References

[1] Findeisen, M.; Brand, T.; Berger, S. “A 1H-NMR thermometer suitable for cryoprobes” *Magn. Reson. Chem.* (2007) **45**, 175-178.
